# Dual-Wide Multi-Band (DWMB) four-port flexible MIMO antenna for on-body multiple wireless applications including high diversity performance

**DOI:** 10.1371/journal.pone.0309690

**Published:** 2024-11-04

**Authors:** Manish Sharma, Kanhaiya Sharma, Prabhakara Rao Kapula, Anand Nayyar, Muhammad Bilal

**Affiliations:** 1 Chitkara University Institute of Engineering and Technology, Chitkara University, Punjab, India; 2 Department of Computer Science and Engineering, Symbiosis Institute of Technology, Symbiosis International (Deemed University), Pune, India; 3 Department of Electronics & Communication, B V Raju Institute of Technology, Narsapur, Telangana, India; 4 Graduate School, Faculty of Information Technology, Duy Tan University, Da Nang, Viet Nam; 5 School of Computing and Communications, Lancaster University, Lancaster, United Kingdom; Griffith University - GC Campus: Griffith University - Gold Coast Campus, AUSTRALIA

## Abstract

The single-input-single-output technology experiences loss of data in the communication channel due to the receiving antenna undergoing fading of the signal impinged on it. Today’s need is faster data transfer with multiple applications in the single antenna with multiple-identical radiating elements, leading to multiple-input-multiple-output_DWMB_ (MIMO_DWMB_) technology. The MIMODWMB configuration with multi-band capability is the objective of the proposed work with applications ranging between microwave-millimeterWave bands. The four-port Dual-Wide Multi-Band (DWMB) MIMO_DWMB_ antenna radiating electro-magnetic-energy is proposed, which generates measured bandwidths of 7.27GHz-34.32GHz (Band 1) and 46.54GHz-71.52GHz (Band 2) including applications Up-link/Down-link Satellite System, X-Band, Ku-Band, ISM 24.0GHz (24.0GHz-24.25GHz), 24.0GHz UWB Band (21.65GHz-26.65GHz), n258, n257/n261 and n263 V-band. The proposed antenna technology is printed on Rogers’s low permittivity substrate with a hexagon patch etched with dual merged-elliptical slot and three identical circular slots to achieve high impedance matching for Band 1. The partial-ground is etched by a rectangular slot for better impedance matching, and two-thin-etched rectangular slits generate 60.0GHz Band 2. The thin substrate, thickness 0.254mm, is utilized for flexible applications without compromising the operation of dual wide bandwidths. The flexible antenna is also subjected to analysis of Specific-Absorption-Rate (SAR) analysis at key frequencies within both the bands and found to be within the standard limit of 1.60W/Kg for 1g of the human tissue model and corresponds to 1.01W/Kg at 10.0GHz, 0.280W/Kg at 15.0GHz, 0.475W/Kg at 26.0GHz, 0.588W/Kg at 28.0GHz & 0.301W/Kg at 60.0GHz. The high diversity performance with Envelope Correlation Coefficient<0.50, Diversity Gain≈10.0dB, Total Active Reflection Coefficent<0dB, Channel Capacity Loss<0.40b/s/Hz and multi-band capability for mobile users make the proposed work suitable for flexible on-body applications in a wireless environment. The proposed work MIMO_DWMB_ antenna offers advantages such as reduced size (20mm×24mm: 0.61λ_0_×0.74λ_0_ at λ_0_ = 7.27GHz) and a wide range of impedance bandwidths, which are useful for several applications. Also, due to the flexible nature of the design, they can be used for future on-body wearable applications.

## 1. Introduction

In recent decades, the demand for antenna front end has increased tremendously with upcoming wireless technologies such as the implementation of 5G for Internet-of-Things (IoT) applications, which has demanded the transfer of larger data over the wireless communication channel. On the other hand, the space occupied by the antenna on the printed circuit board (PCB) must be very compact to pay the path for several other integrations of Microwave Integrated Circuits (MIC) circuits. In view of the above demands, a literature review is carried out to understand the existing wireless communication technology focusing on the front-end antenna component. The flexible-antenna configuration review on 5G communication was proposed by Gupta et al. [1], discussing the calculation of multiple-input-multiple-output (MIMO)-diversity parameters. The mutual coupling reduction techniques are mentioned [2] which are achieved by using slot, stub, split-ring-resonator, parasitic element, fractal element, Electromagnetic-band-gap structure, etc., A optically-transparent four-port MIMO antenna with optimal size of 480 mm^2^ [3] offers dual-band of operation with Band-1 of 24.10GHz-27.18GHz and Band-2 with bandwidth of 33.0GHz-44.13GHz. The MIMO antenna utilizes a transparent substrate combining AgHT-8 & Plexiglass. A UWB-four-port MIMO antenna with a bandwidth of 3.10GHz-12.0GHz with an octagonal-geometry patch achieves Envelope Correlation Coefficient (ECC)<6×10−^4^ [4] and a sickle-shaped four-port ensures lower bandwidth applicable for 5G communication [5]. MIMO antenna finds applications in numerous wireless systems such as ultra-wideband (UWB), X-band, Ku-band, 24.0GH band, FR2 bands: n257 (26.50GHz-29.50GHz), n258 24.25GHz-27.50GHZ), n263 (57.00GHz-71.00GHz) [6, 7].

Also, it is noted that the large bandwidth ratio MIMO antenna maintains diversity parameters within the permissible limits. The wider-impedance bandwidth is achieved by using radiating geometry such as a 12-sided polygon, vase-shaped patch, or simple hexagon patch. The application of Dielectric-Resonator-antenna (DRA) placed over the patch offers a narrow bandwidth of 5.52GHz-6.20GHz with a percentage bandwidth ratio of 11.60% and the MIMO version achieves ECC<0.042 [8]. A multiband antenna [9] producing resonances that are centered at 28.30GHz, 38.10GHz, 46.60GHz, and 60.0GHz offering peak directivity (broadside) of 7.80dB, 8.80dB, 7.30dB, and 7.10dB is developed by applying binary-coded genetic algorithm. The millimeter wave 5G-MIMO antenna operating in FR2 bands (28.0GHz and 38.0GHz) are reported [10–15] and occupies very little space on PCB in MIC applications. The four-port MIMO array with etched slots in the ground offers an operating bandwidth of 25.50GHz-29.60GHz and maintains isolation of more than 17.0dB [10]. Also, a circular-slotted patch resonating 28.0GHz [11] offers stable-2D radiation patterns with a peak gain of 4.49dB with a corresponding efficiency of 89%. A rectangular-ring antenna with embedded T-shaped stubs and rectangular etched slots in the ground is designed for a four-element array configuration and offers a gain of 11.50 dBi at 28.0 GHz [12]. Low profile dual-band single-port antenna with resonance centered at 28.0GHZ/38.0GHz utilizes a meta-material structure with a closed-ring and split-ring-resonator [15]. The higher 5G-millimeter wave band at 60.0GHz resonating antennas are reported in [16–28] with I-type patch offering resonance at 60.0GHz and 90.0GHz [16].

Circular-patch antenna etched with rectangular-slot and partial are arranged in an orthogonal configuration with four pairs and achieves isolation by using isolated rectangular strips in the ground [19]. Conformal antennae with bending capabilities was proposed by Semkin et al. [20] which was dependent on the substrate used such as Taconic-5 with permittivity of 2.20 and thickness of 0.127mm. The antenna array at 60.0GHz provides gain variation between 12.0dBi-16.0dBi with bandwidth 56.50GHz-65.20GHz [21]. The conformal antenna [23] was reported with dimension 50×43 mm2 and generates resonance of 7.20GHz and 9.20GHz. Also, a 60.0GHz wideband antenna with a bandwidth of 57.24GHz-65.88GHz achieves a high directive gain of 18.55dB [24–28]. Liquid Crystal Polymer (LCP) substrate with a four-port MIMO antenna of low profile of dimension 65×65×0.1 mm3 offers impedance-bandwidth of 2.90GHz-10.86GHz with ECC less than 0.01 [29, 30]. A Ultra-wideband (UWB)-capability with high isolation comprising of four ports offers impedance-bandwidth of 25.0GHz-50.0GHz with high diversity parameter [31] and specific-absorption-rate (SAR) reduction technique was proposed by Kumkhet et al. [32] with the use of electromagnetic-band-gap and constructed square loop plate reflector [33, 34]. The truncated E-shaped patch and defected-ground sows the capability of generating Ku-band [35] and the link budget for 5G communication was proposed by Juneja et al. [36]. The breast-cancer models was proposed by Zerrad et al. [38] which was applied by UWB-bandwidth. The breast-phantom models are subjected to microwave-breast-imaging and the methodology to prepare the model was also discussed. Two four-port MIMO antenna [39–41] maintains good diversity characteristics by placing the radiating elements in orthogonal-sequence. Flexible and MIMO-antenna for W-BAN was discussed by Jhunjhunwala et al. [42] which focused on bandwidth in Ultra-Wideband applications [43, 44]. A dual-band 4-port MIMO antenna is studied for flexible applications with bending maintaining the non-deviation of the dual-band [45]. The bending materials such as the Rogers 3003 substrate with a thickness of 0.13mm [46], and the PET substrate with a thickness of 0.10mm [47–51] are used for flexible applications with SAR analysis. A Mickey-shaped Super-wideband antenna achieves bandwidth of 1.22GHz-47.50GHz [52] which finds its applications in Microwave & millimeter wave bands applications.

5G-MIMO antenna with eight-port configuration fabricated on FR-4 substrate shows the capability of dual-polarization with 5G-bandwidth of 3.30GHz-4.10GHz [53]. A wideband MIMO antenna [54] offers bandwidth of 25.0GHz-38.0GHz achieves isolation of more than 18.0dB which is achieved by placing the radiating-patch in orthogonal-sequence. Cactus-shaped patch with reconfigurable configuration [55, 56, 63] produces bandwidth suitable for Wireless Interoperability for Microwave Access (WiMAX), Wireless-Fidelity (Wi-Fi), Bluetooth, Long Term Evolution (LTE) and satellite X-band communication. V-sshaped with staircase ground achieves UWB-bandwidth and includes several wireless-applications such as ISM-band, WiMAX, 5G-sub-6.0GHz. A Square-framed patch with rectangular-slotted ground [57] operates at 28.0GHz mmWave band with 94% efficiency. In [58], tolerance-analysis was studied for Petal-Reflector-Antenna (PRA) utilizing Random-surface and errors due to deployment. The use of Sensors for intelligent tactile-sensing was reported with equipped Soft-Gripperes [59], and a detailed systematically review was studied for commercially available Wearable-Activity-Trackers [60]. The Internet-of-Things (IoT) application antenna was developed which can reconfigure for both beam and pattern operates within the bandwidth of 4.95GHz-7.60GHz [61]. The a-novel de-coupling structure was used on the same plane of UWB-antenna with a bandwidth of 3.10GHz-11.80GHz, achieving isolation of more than 20.0dB [62–64]. A tri-band antenna with CPW-feed is achieved by modifying Square-patch to tuning-fork-shaped, offering applications in 5G-6G wireless communication [65].

The objective of the paper is to introduce MIMODWMB functionality and to cover numerous wireless application bands. This is achieved by designing the antenna for wider-impedance bandwidth with integrated two bands, Band1 = 7.27GHz-34.32GHz and Band2 = 46.54GHz-71.52GHz. These two bands ensure applications such as uplink/downlink satellite systems in X-band with a downlink band of 7.25GHz-7.75GHz and an uplink band of 7.75GHz-8.30GHz. The other application bands include applications in Ku-band, 24.0GHz ISM/UWB band, n258, n257/261 and n263 bands.

### 1.1. Organization of paper

The rest of the paper is organized as: Section II centers on the design of single-port multi-band antennas with bending capability and their applications on the human body. In Section III, the focus shifts to the conversion of single-port antennas into two-port and four-port configurations, accompanied by an in-depth analysis including far-field results, bending angles S11 characteristics, SAR calculation within application bands, and diversity parameters. Finally, Section IV concludes the paper with future scope.

## 2. Design of single-port antenna analysis on thin substrate with bending and on-body applications

As discussed, the demand for compact size antenna needs to be developed with additional features such as wider impedance bandwidth. This designed antenna will cover several wireless communication bands including satellite Up/Downlink, X-Band, Ku band, Ka band, 24.0GHz band, n257, n258, and n261. The additional capability of flexibility will add an advantage for on-body wireless applications. The design methodology with evolution and relevant analysis is discussed below

### 2.1. Single-port antenna configuration

**Fig 1:**
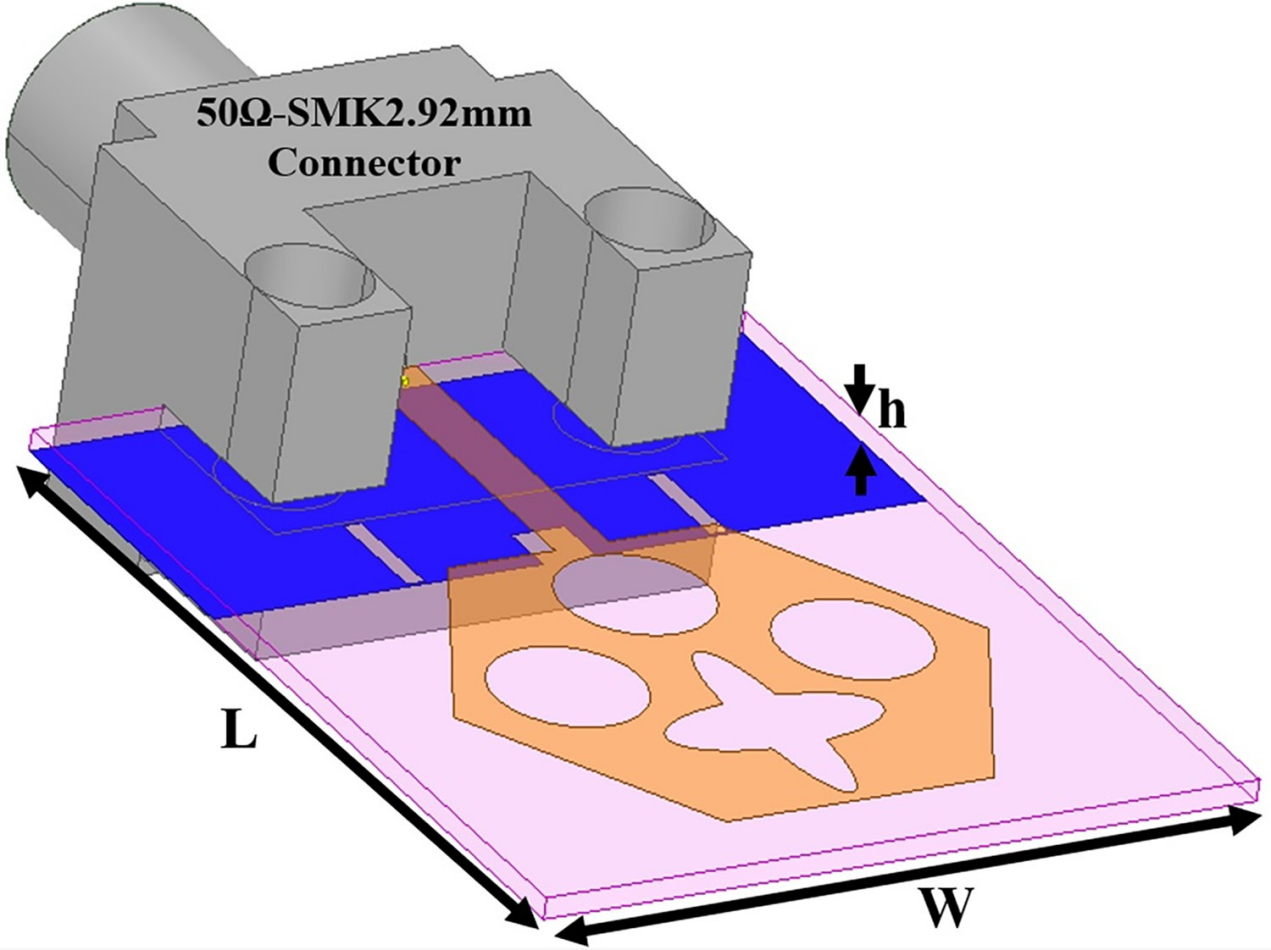
Dual-band antenna configuration with oriented view including compact dimensions.

**Fig 2:**
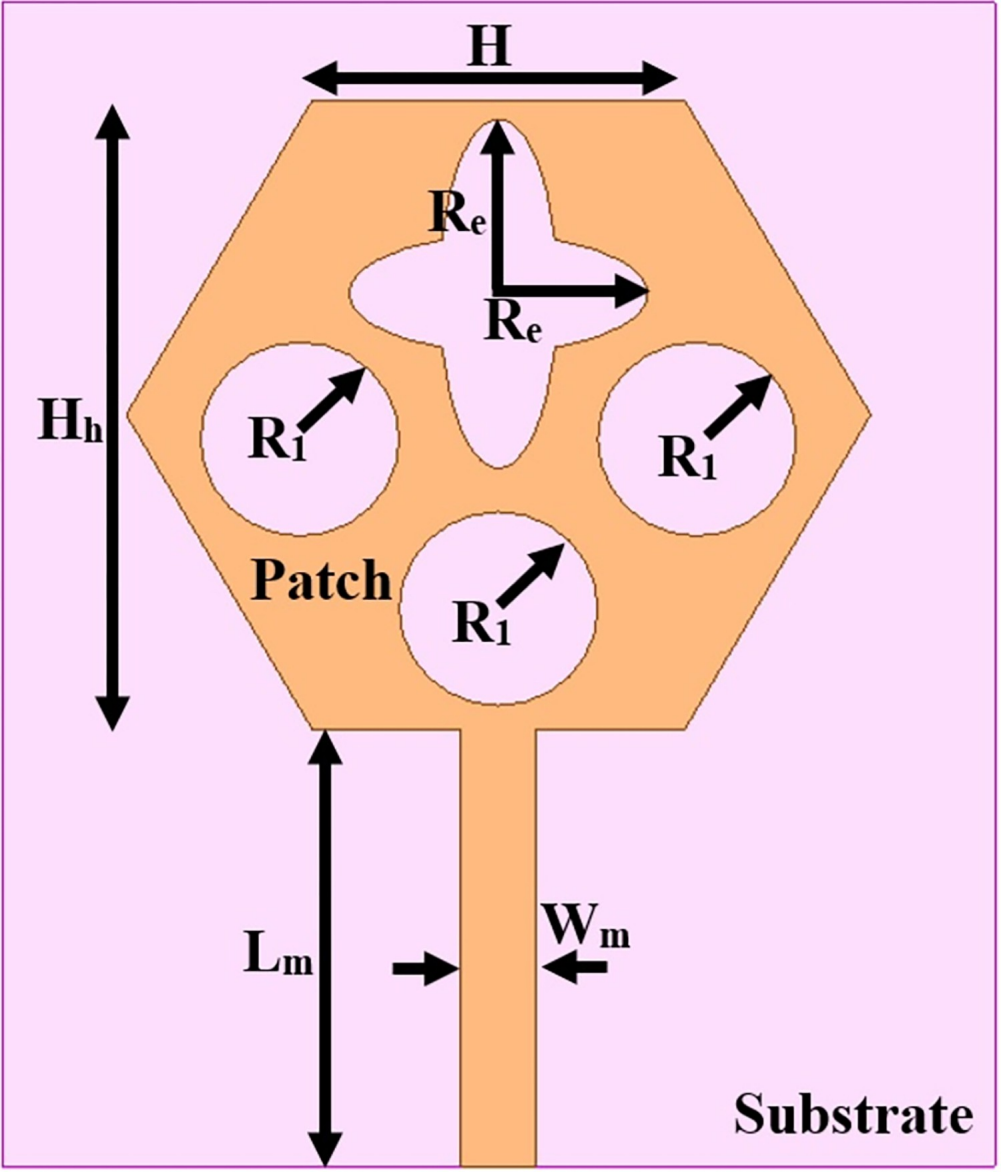
Radiating patch printed on top-plane.

**Fig 3:**
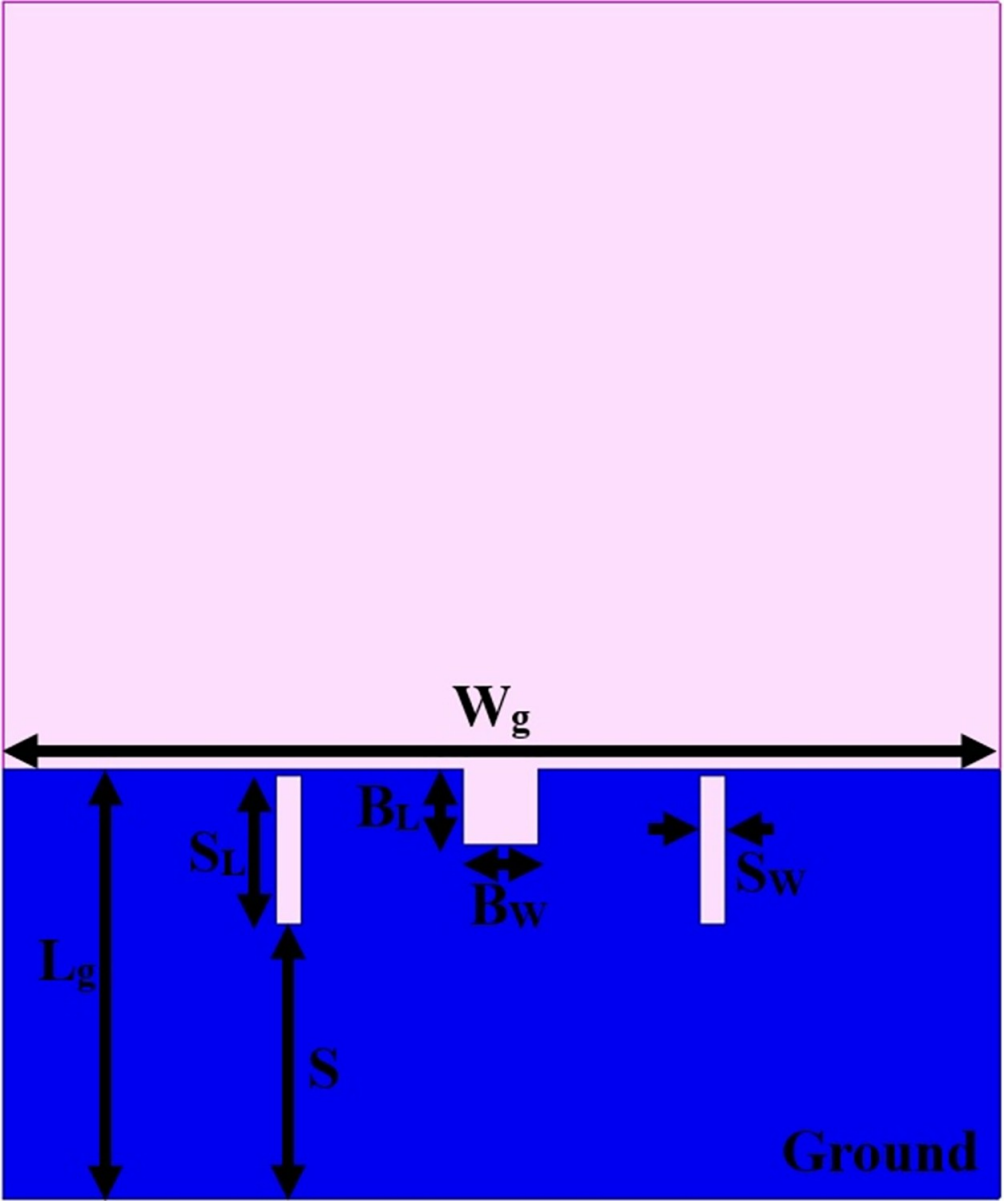
Ground dimensions.

The antenna’s wider-impedance bandwidth with multiband capability including flexible characteristics is needed in today’s scenario of wireless communication and the designed antenna is shown in Figs 1–3. This designed antenna will cover several wireless communication bands and can replace multiple which are integrated with a printed circuit board (PCB). The antenna is designed to fulfil the above objective and Figs 1–3 shows the detailed structure with optimal dimensions.

Fig 1 represents the printing of a radiating patch on the top plane and ground on the opposite plane with an antenna dimension of L×W mm^2^. The radiating patch is fed by 50Ω modeled SMK1.85mm connector for input signal from RF-source. The height of the substrate is **h** mm and is very thin in thickness which makes it a suitable candidate for conformal-on body applications. Fig 2 shows the radiating patch on the top surface of the Rogers RTDuroid5880 substrate. The patch is of the geometry with side **H** mm with an integrated etched flower-type slot and three circular slots of radius R_1_.

Fig 3 shows the printing of partial ground on a surface opposite to the printed patch. The ground is beveled by a rectangular slot of area B_L_×B_W_ mm^2^ and two slits placed at a distance **S** from the bottom edge of the substrate. All the dimensions shown in Figs 1–3 are optimized which is implemented in EM-simulator Ansys HFSSv15 with dimensions recorded in Table 1 as shown

**Table 1:** Optimized parameters.

Par.	mm	Par.	mm	Par.	mm
L	12.00	W = W_g_	10.00	h	0.254
L_m_	4.75	W_m_ = B_W_	0.80	H	3.75
H_h_	6.50	R_e_	2.00	R_1_	0.80
W_g_	10.00	L_g_	4.60	B_L_	0.80
S	3.00	S_L_	1.50	S_w_	0.20

### 2.2. Evolution of the proposed antenna

**Fig 4:**
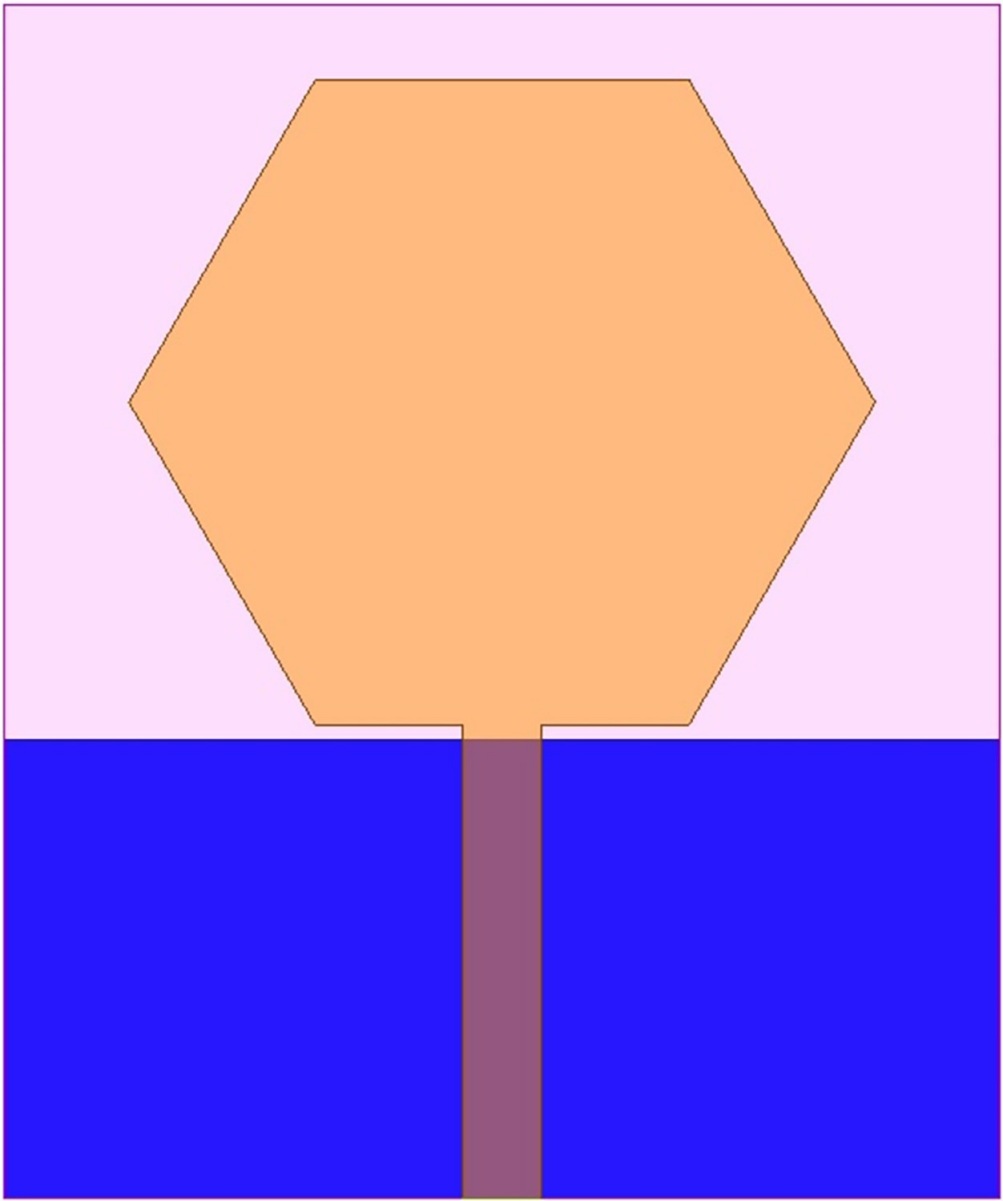
Evolution A_a_.

**Fig 5:**
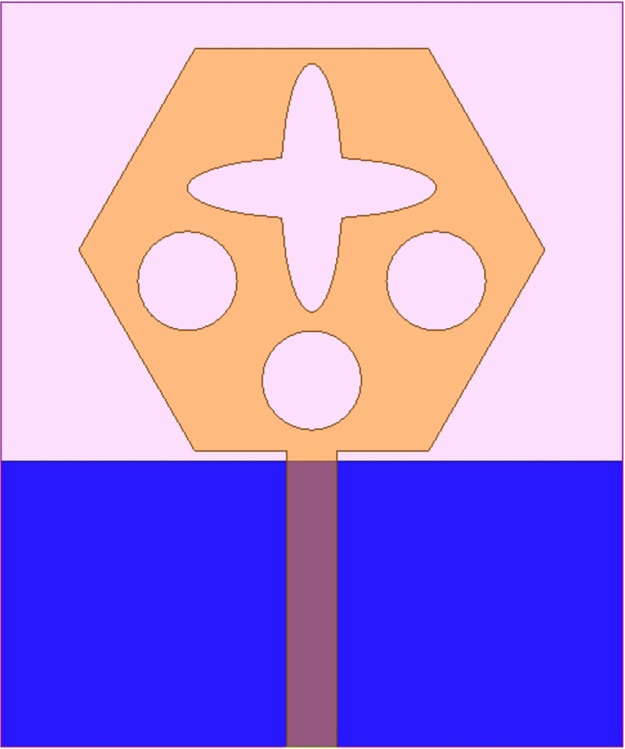
Evolution A_b_.

**Fig 6:**
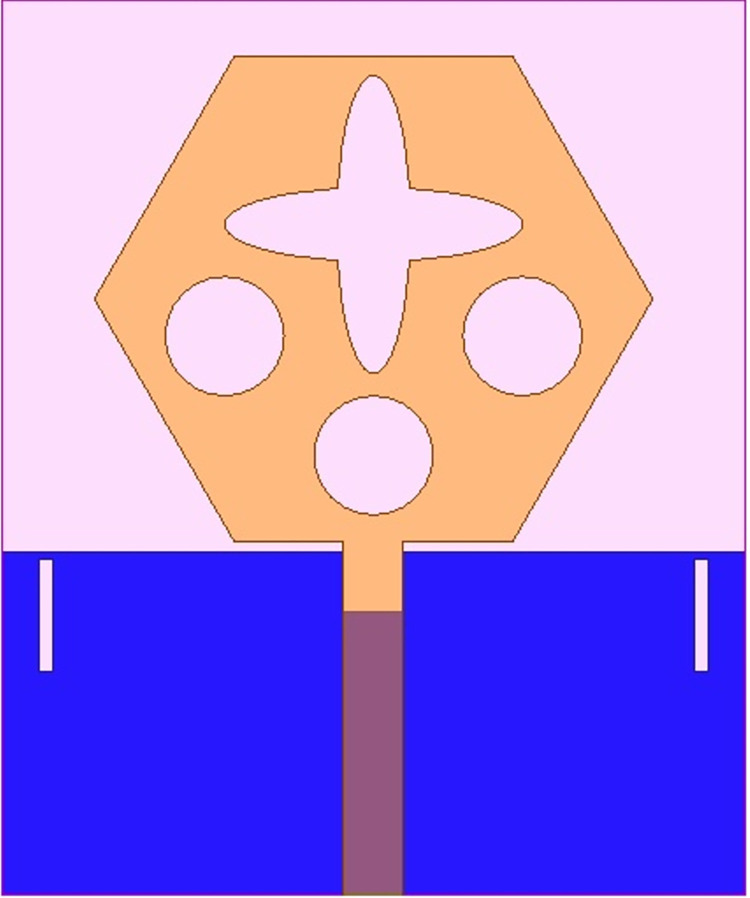
Evolution A_c_.

**Fig 7:**
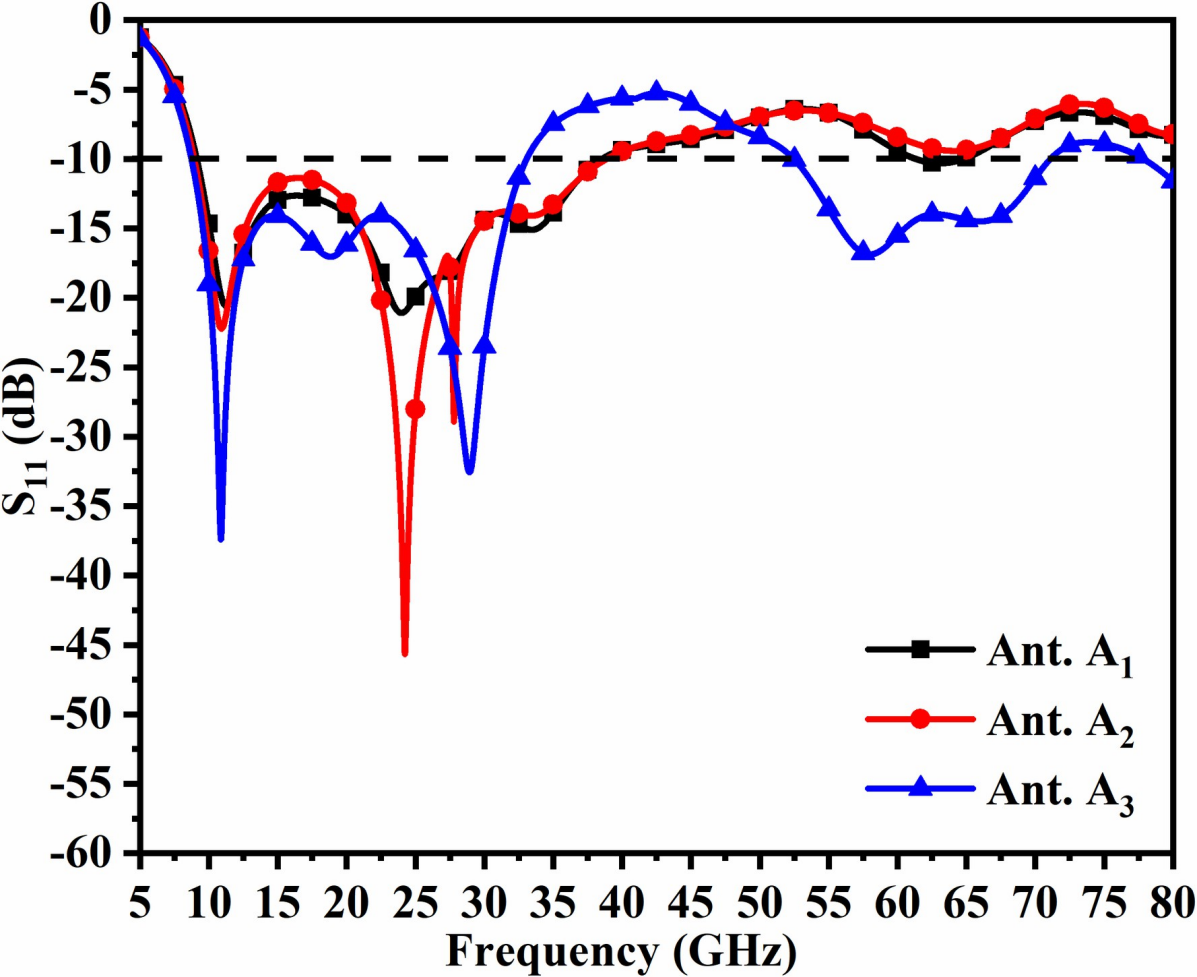
S_11_ of Antenna A_a_-Antenna A_c_.

Dual-band operational antenna is evolved by subjecting several iterations which is discussed in detail represented in Figs 4–7. The design of the proposed work is initiated by using a hexagon patch printed on the top plane and rectangular ground on the opposite plane with a total area of 120 mm^2^. The optimized hexagon side, **H** mm is calculated by using the following formula [37]

$$H=\frac{k}{4\times f_{t}}\sqrt{1/(1+\varepsilon_{p})}$$ (1)

Where k (vacuum medium for speed of light = 3×10^8^ m/s), ft corresponding to resonant frequency (f_t_ = GHz), pseudo-permittivity (ε_p_) and is obtained by Eq 2 [37]

$$\varepsilon_{p}=\frac{\varepsilon_{r}+1}{2}+\frac{\varepsilon_{r}-1}{2}{\left[1+\frac{12h}{W_{m}}\right]}^{\frac{1}{2}}$$ (2)

The antenna shown in Fig 4 corresponding to Ant. A_1_ offers -10.0dB matched impedance bandwidth of 9.12GHz-38.53GHz as noted in Fig 7. This bandwidth is useful for applications in X-band, Ku-band, K-band, n257, and n258 5G bands. However, the etching of the radiating patch by dual-orthogonal placed elliptical slot in addition to three circular slots of radius R_1_ shown in Fig 5 improves the matching of impedance corresponding to Ant. A_2_ with bandwidth 8.93GHz-38.87GHz. The improved matching of impedance offers three resonances centered at 10.91GHz with S_11_ = -22.22dB, 24.23GHz with S_11_ = -44.67dB, and 27.79GHz with S_11_ = -28.92dB. The third iteration in Fig 6 represents Ant. A_3_ is achieved by etching a rectangular slot of size B_L_×B_W_ mm^2^ and two rectangular slits of area S_L_×S_W_ mm^2^. This modification results in an extra 5G-millimeter bandwidth of 8.73GHz-33.0GHz and 52.46GHz-71.09GHz. The proposed antenna covers dual bands with applications in X-band, Ku-band, K-band, n258, n257, and n263 bands.

### 2.3. Surface_current density_dwmb_ (SCD_DWMB_) study, parametric study of key-parameters (H, BL, SL) and bending capability

**Fig 8:**
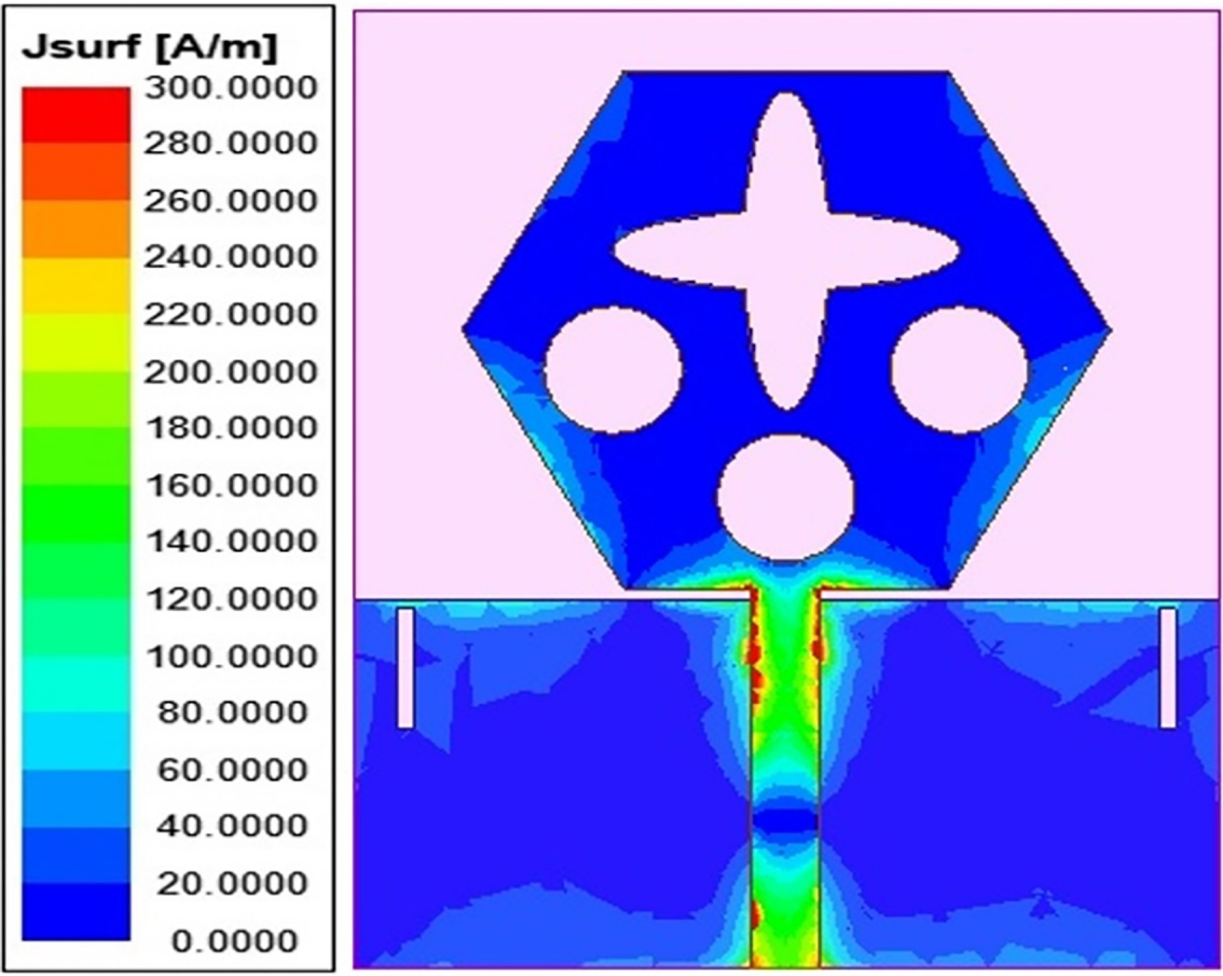
SFD_MB_ at 10.90GHz.

**Fig 9:**
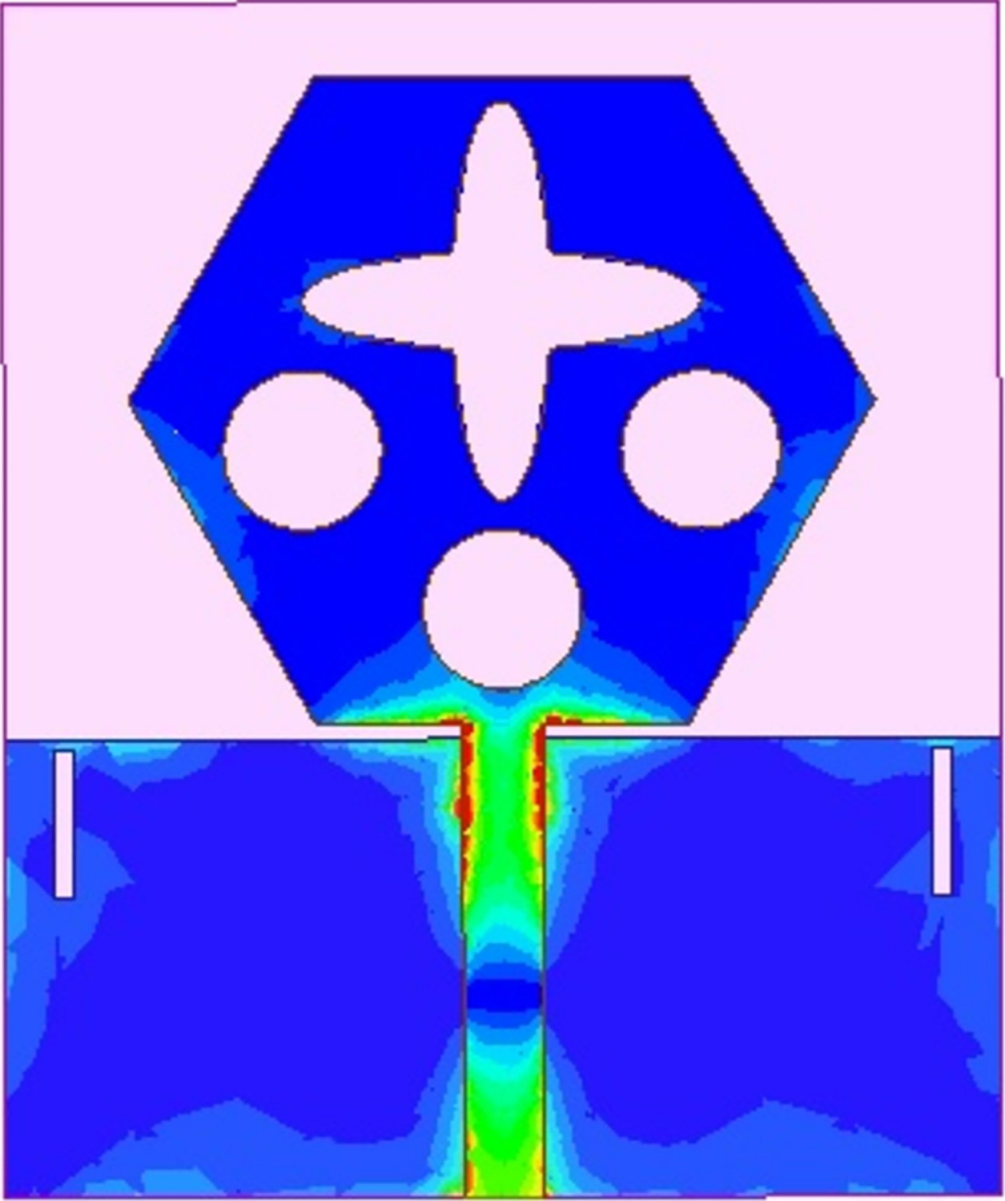
SFD_MB_ at 15.0GHz.

**Fig 10:**
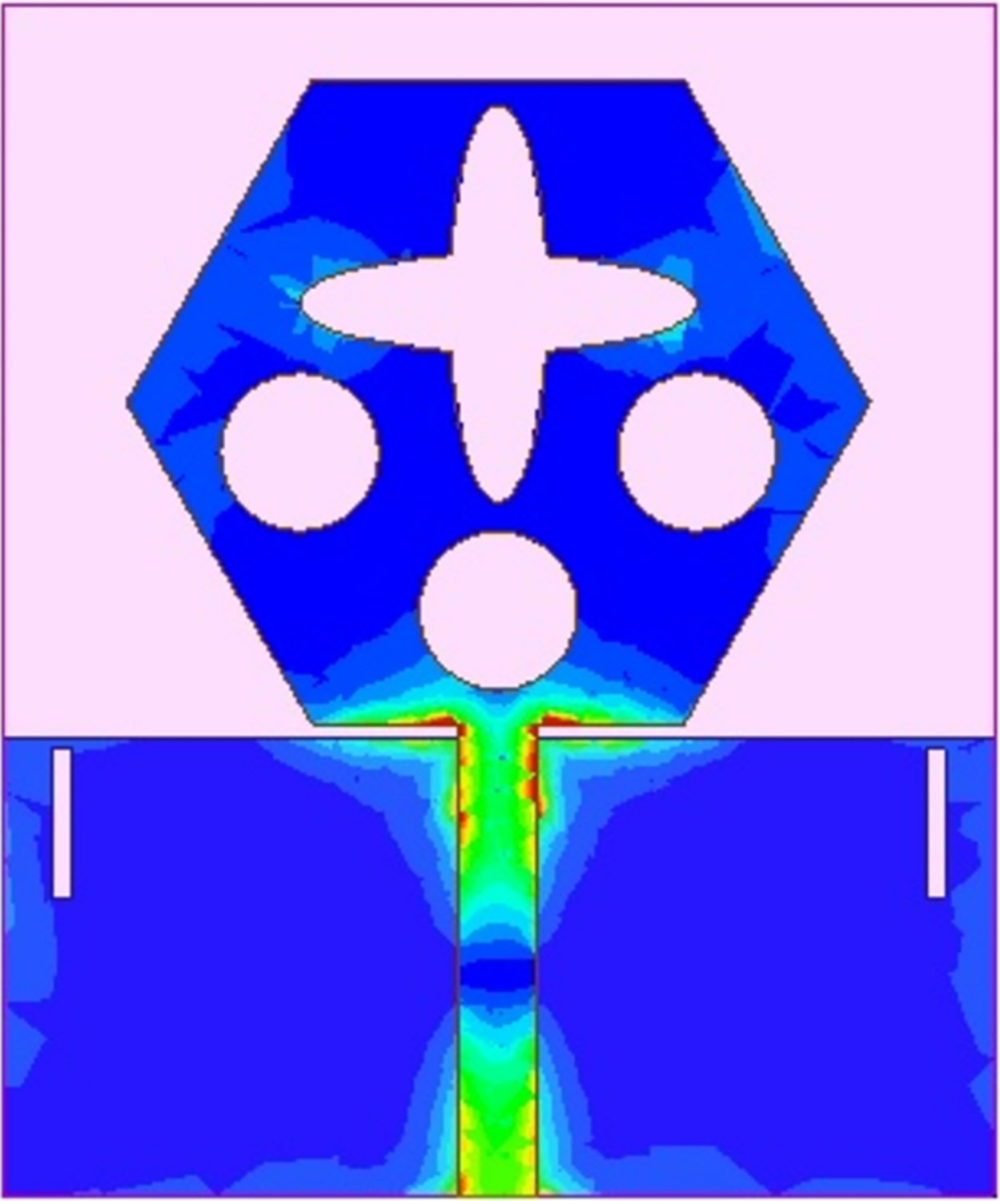
SFD_MB_ at 24.0GHz.

**Fig 11:**
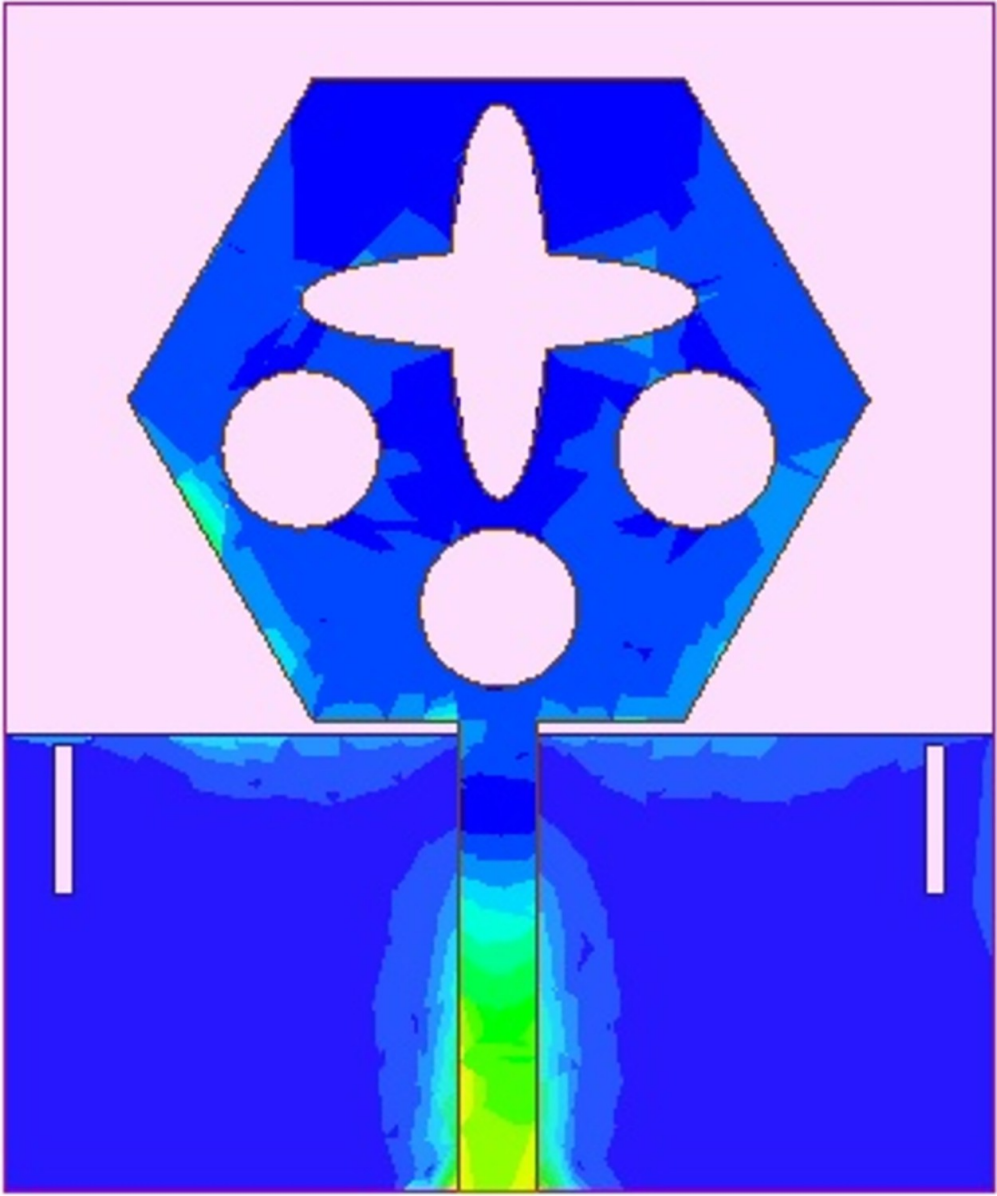
SFD_MB_ at 26.0GHz.

**Fig 12:**
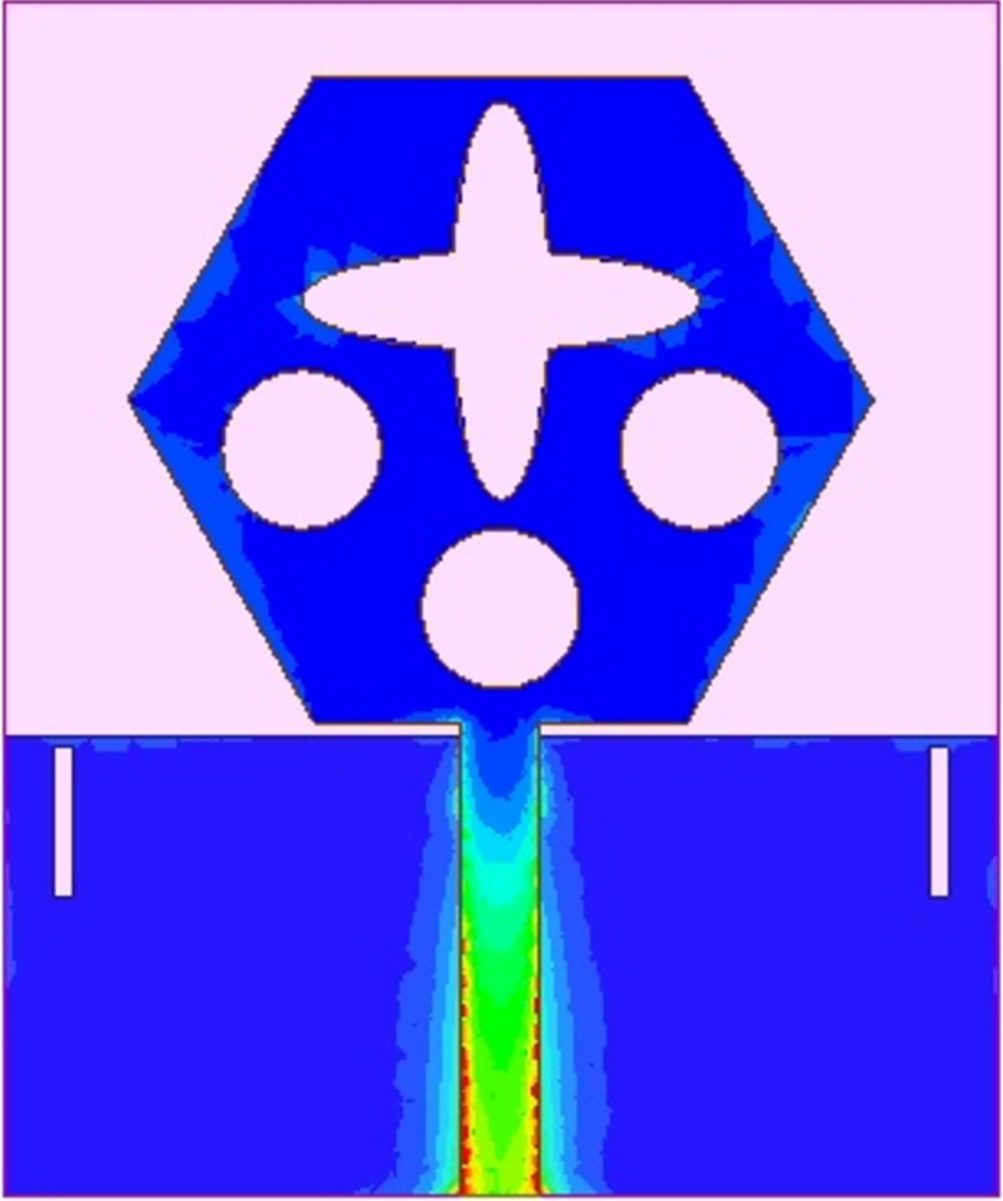
SFD_MB_ at 28.0GHz.

**Fig 13:**
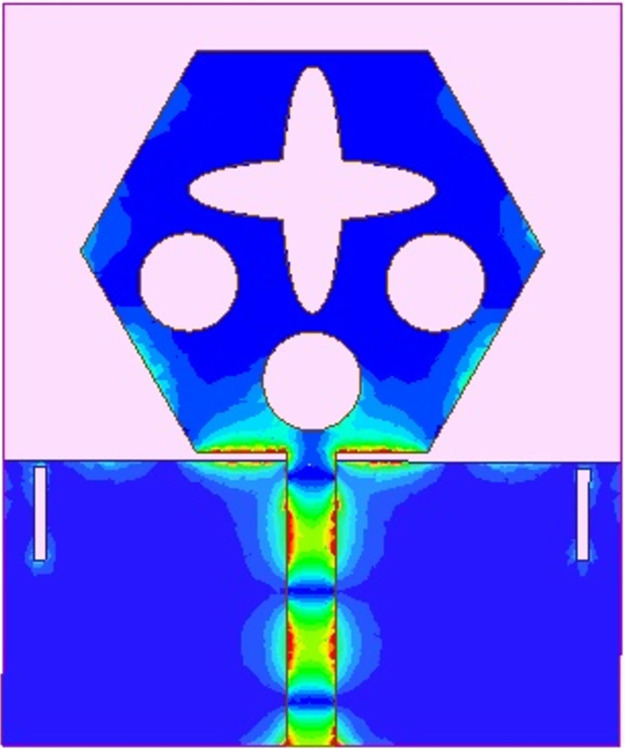
SFD_MB_ at 60.0GHz.

The spreading of surface-current-density_DWMB_ both on patch & ground are observed in Figs 8–13 over selected frequency points corresponding to 10.90GHz (X-Band), 15.0GHz (Ku-Band), 24.0GHz (K-band), 26.0GHz (n258), 28.0GHz (n257) and 60.0GHz (n263).

For all the six frequency points shown in Figs 8–13, surface-current-density is accumulated within the 50Ωmicrostrip feed because the feed line carries the input signal to the patch.

Moreover, at these 6-frequency points, the surface current density is evenly distributed over the patch indicating good radiation characteristics. The parameters marked in Figs 1–3 and tabulated in Table 1 correspond to optimized values.

**Fig 14:**
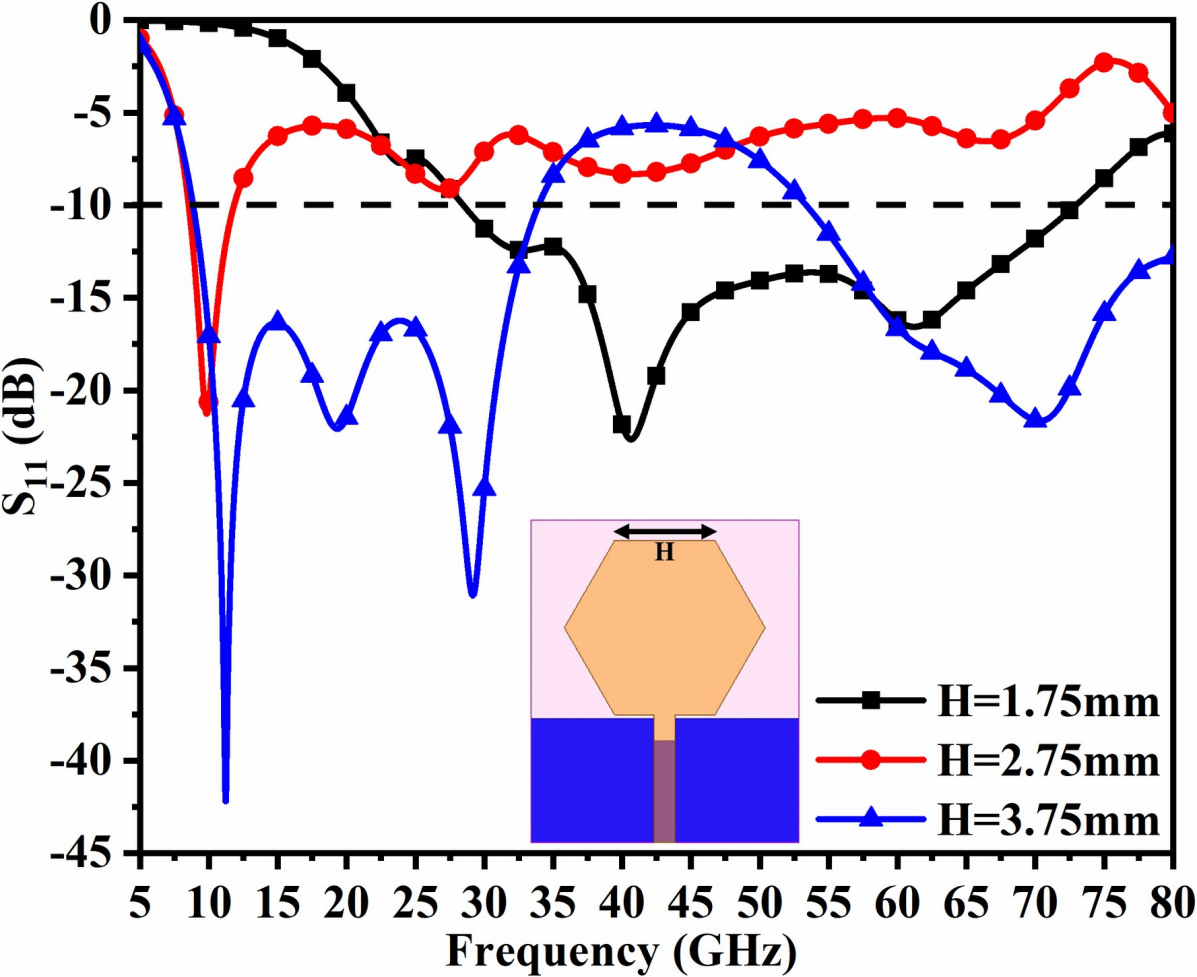
Parametric study of H.

**Fig 15:**
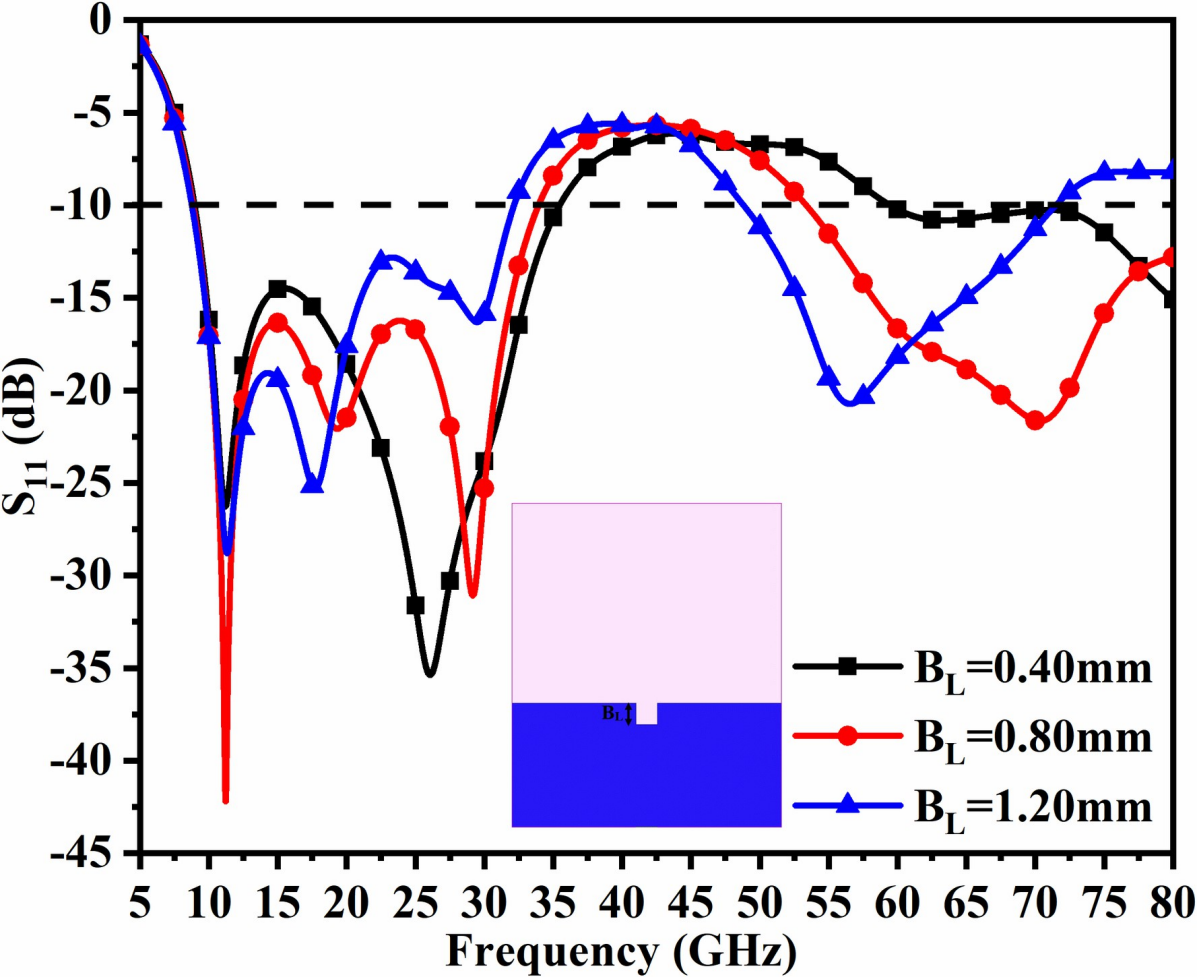
Parametric study of B_L_.

**Fig 16:**
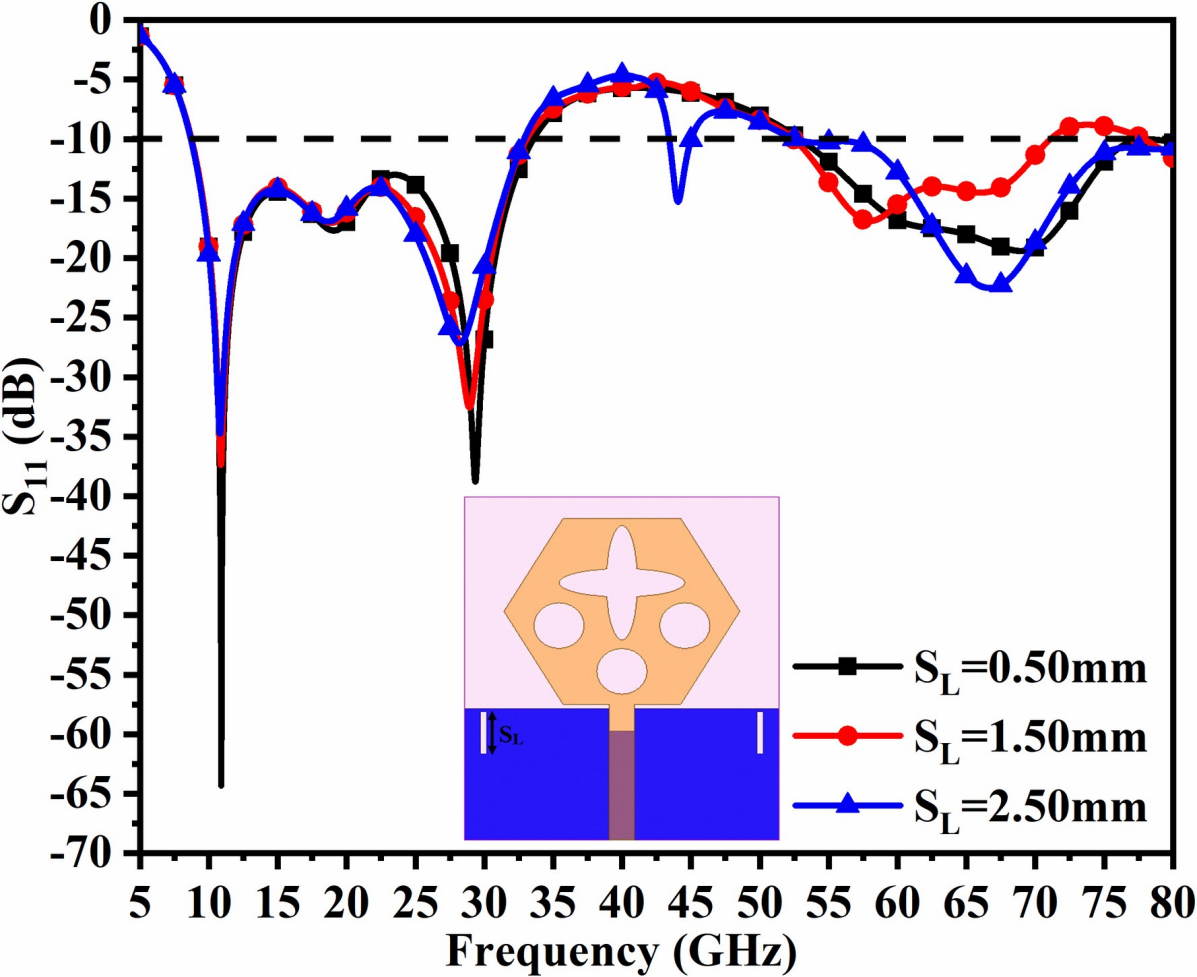
Parametric study of S_L_.

**Fig 17:**
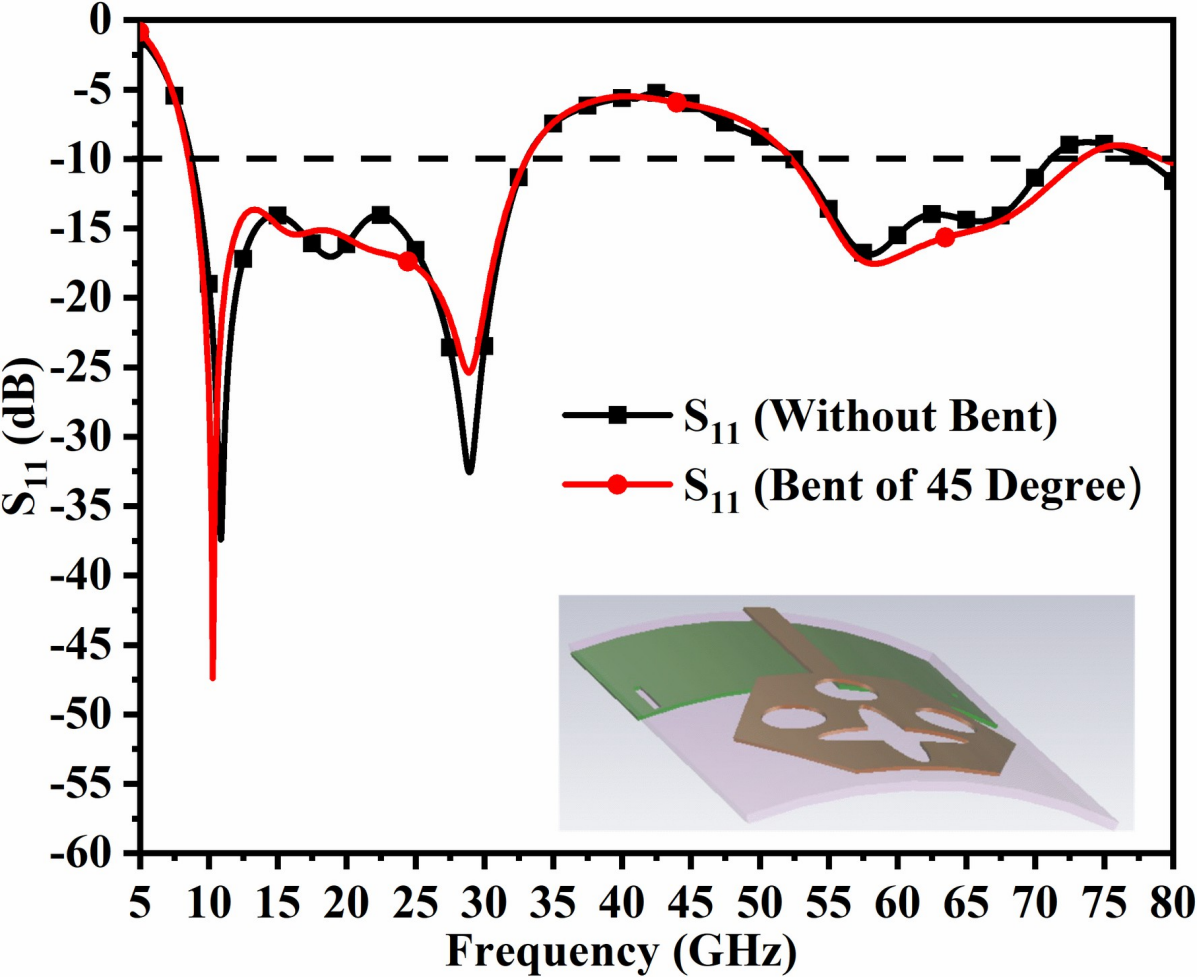
S_11_ comparison of bending of antenna.

However, in designing and coming out with the final version, the parameters are subjected to variations that do affect the matching of impedance, and is discussed in Figs 1, 15 and 16. The key parameters include **H** (side length of the polygon patch), B_L_ (length of the etched-rectangular slot in the ground), and S_L_ (length of the etch slit in the ground). For the side length of the polygon, **H**, the change in length from 1.75mm to 3.75mm observes a change in the matching of impedance or variation in S_11_ as shown in Fig 14. For **H** = 1.75mm, the higher frequency bandwidth corresponds to 28.47 GHzs-72.94GHz and for **H** = 2.75mm, the antenna offers a bandwidth of 8.53GHz-11.82GHz. This shows the bandwidth for **H** = 2.75mm becomes narrow compared with **H** = 1.75mm. The desired dual bandwidth is obtained with the first band corresponding to 8.85GHz-33.92GHz and the second band 53.37GHz-80.0GHz for **H** = 3.75mm. The second parameter that needs to be optimized is B_L_ which is the depth of the rectangular slot etched in the ground for better impedance matching shown in Fig 15. The variations from 0.40mm to 1.20mm observe the improvement of matching of impedance at second-band with the first band is unchanged (8.85GHz-33.92GHz). The value of B_L_ = 0.40mm is incapable of generating the second band while the value of B_l_ = 0.80mm generates a bandwidth of 48.82GHz-71.49GHz. The Two slits etched in the ground observed from Fig 16 with length S_L_ also affect the cut-off frequency at Band-2 with no change in Band-1 bandwidth. The values of S_L_ = 0.50mm have higher off frequency at 70.0GHz band and for S_L_ = 1.50mm, the two bands, with Band-1 impedance bandwidth corresponds to 8.73GHz-33.06GHz and Band-2 with 52.46GHz-71.09GHz. The value of S_L_ is useful for multi-band applications for X-band RADAR, and higher millimeter FR-2 bands. Fig 17 shows the capability of the proposed single-port antenna which can be converted to conformal configuration, and the bent at 45° offers the S_11_ (-10.0dB bandwidth) overlapping with the bandwidth (non-conformal) 8.73GHz-33.05GHz (Band-1) and 52.46GHz-71.09GHz (Band-2).

**Fig 18:**
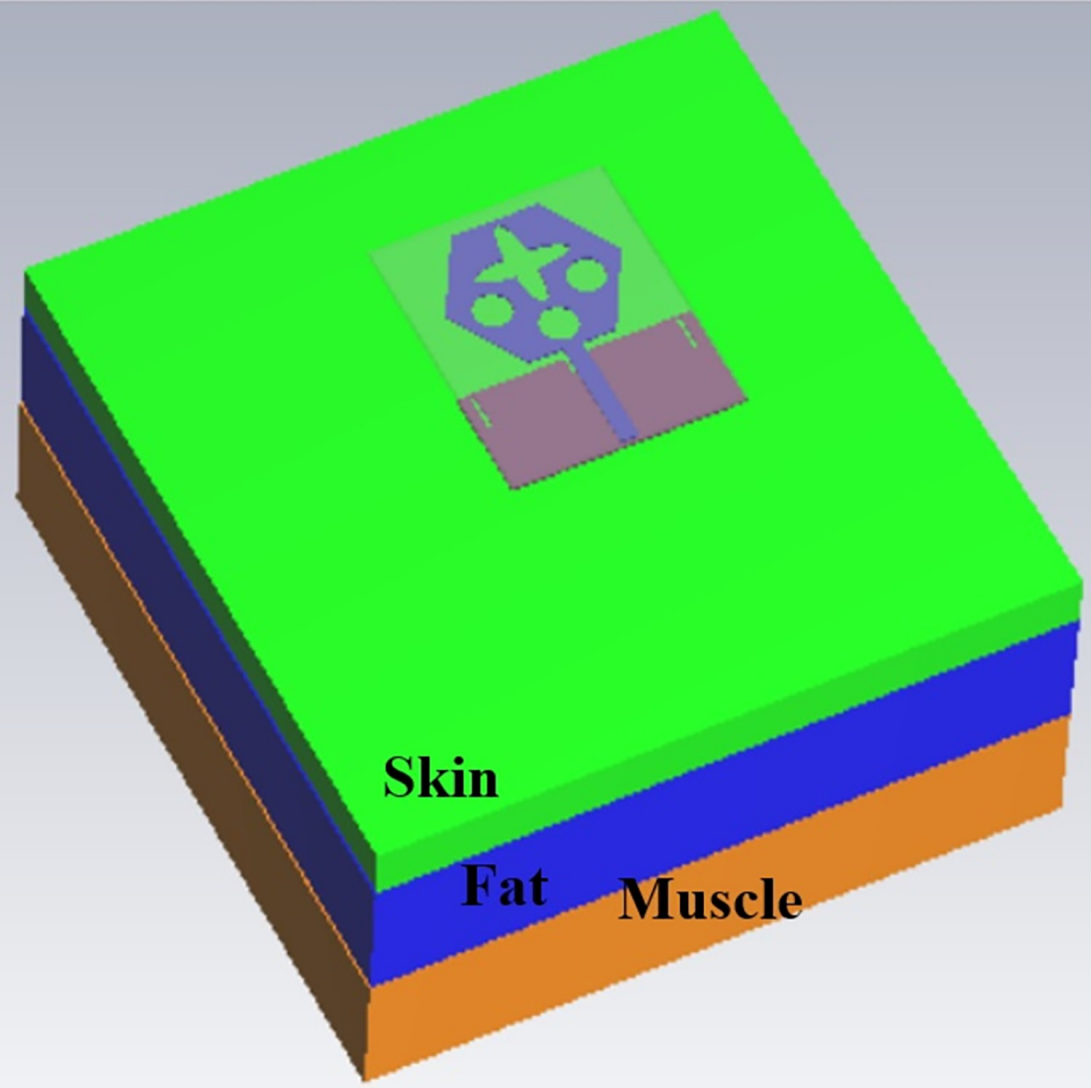
Isometric view of the proposed antenna placed above the Phantom tissue.

**Fig 19:**
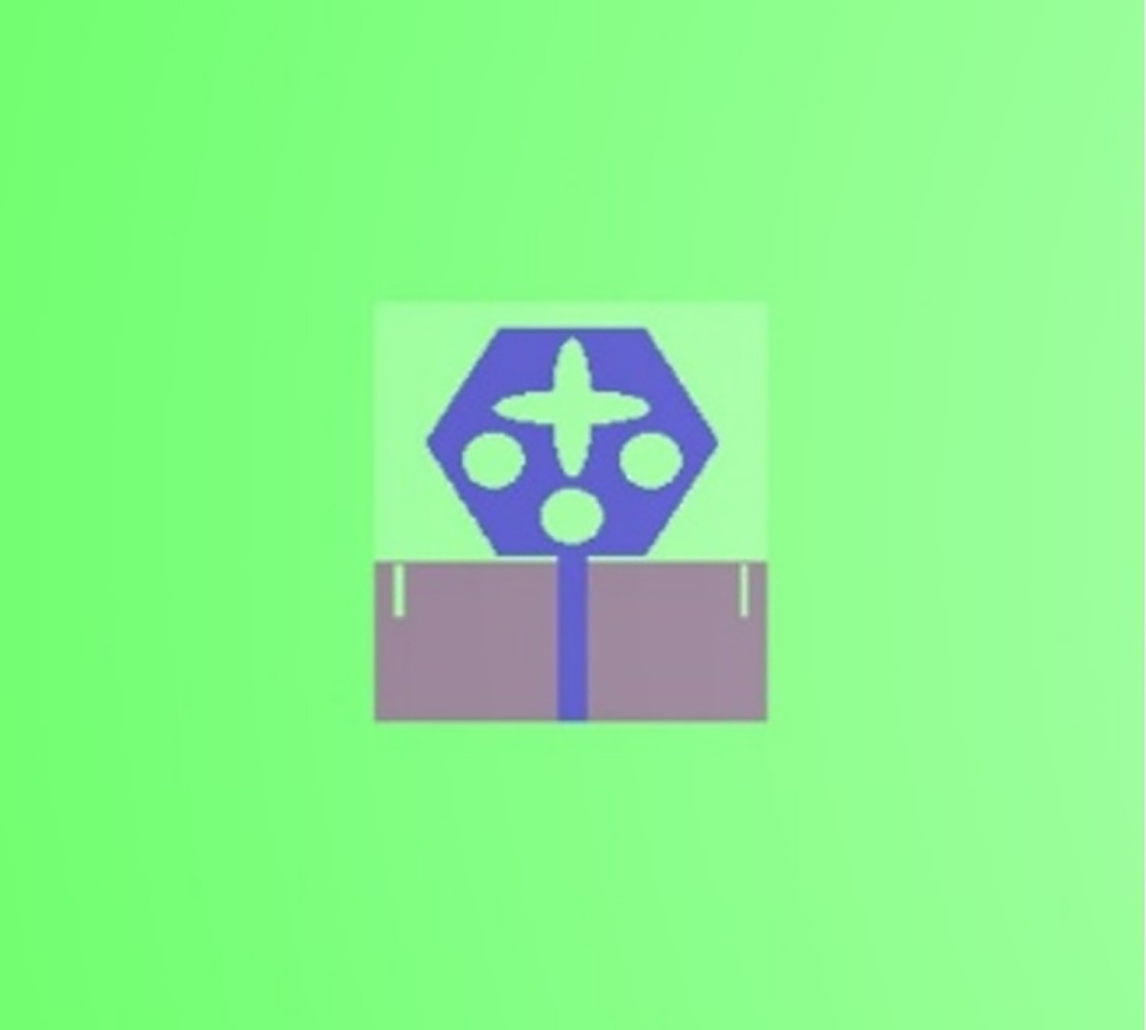
Front-view of the antenna with tissue model.

**Fig 20:**
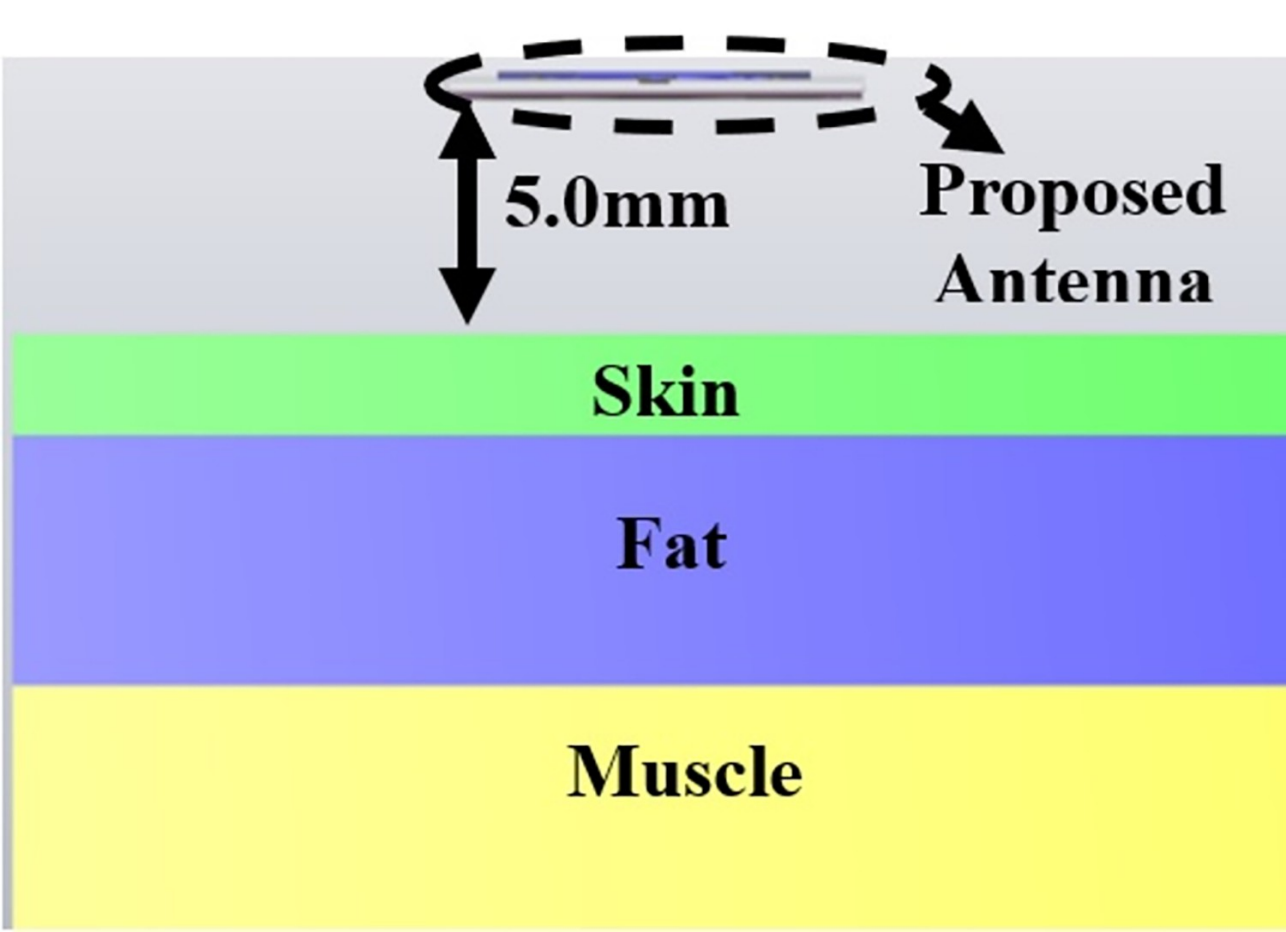
Placement of the antenna at 5.00mm distance abive tissue model.

**Fig 21:**
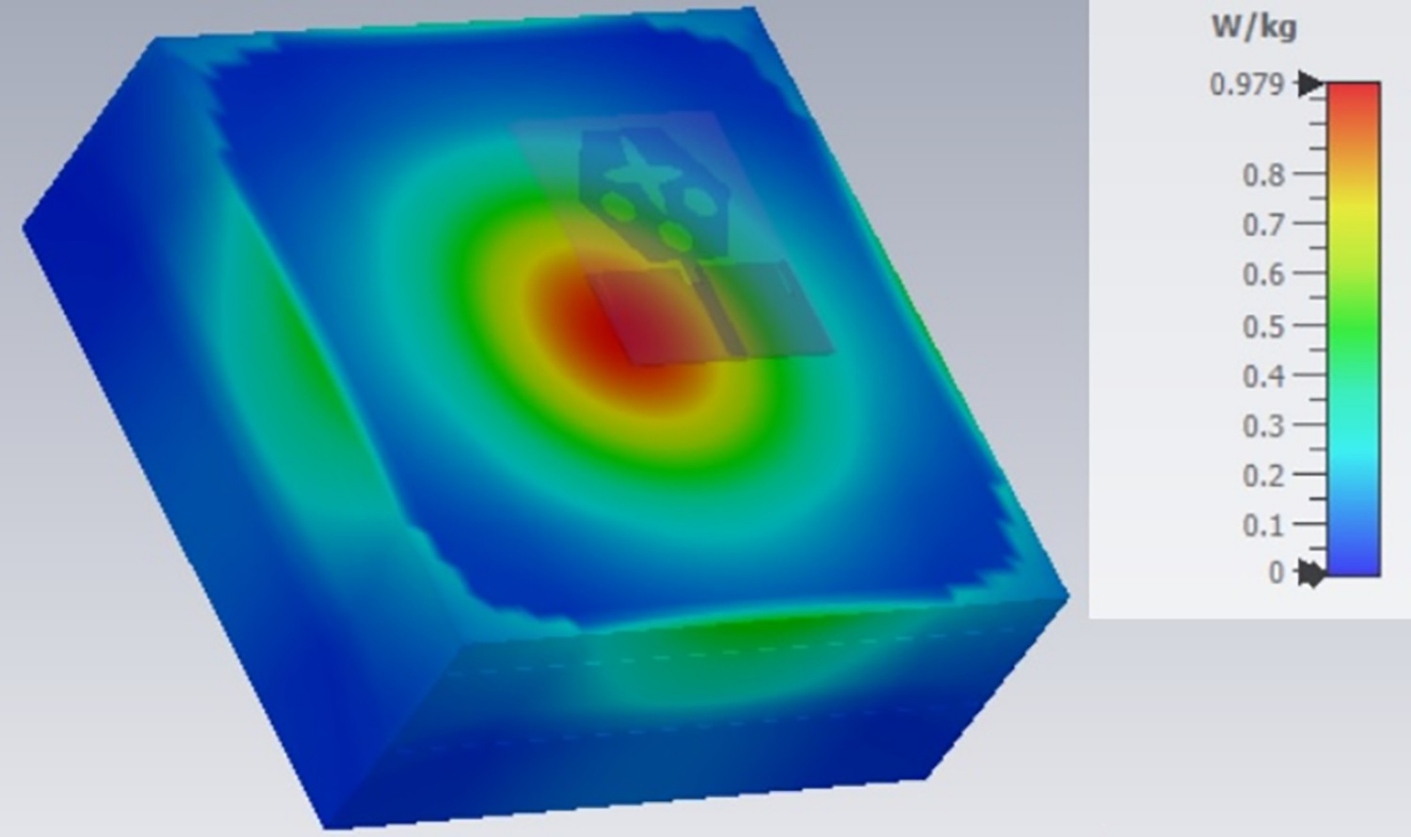
SAR analysis at 10.90GHz.

**Fig 22:**
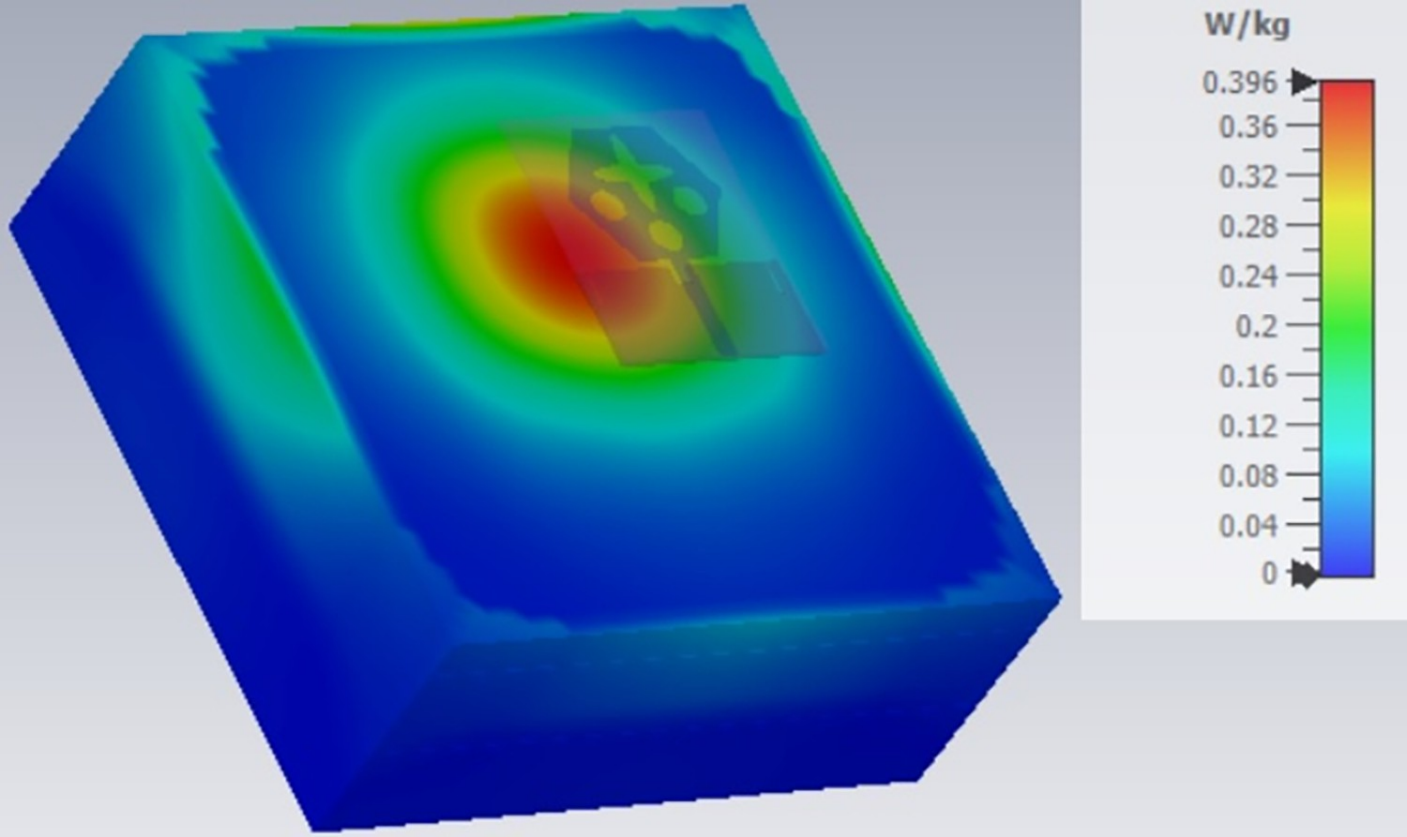
SAR analysis at 15.0GHz.

**Fig 23:**
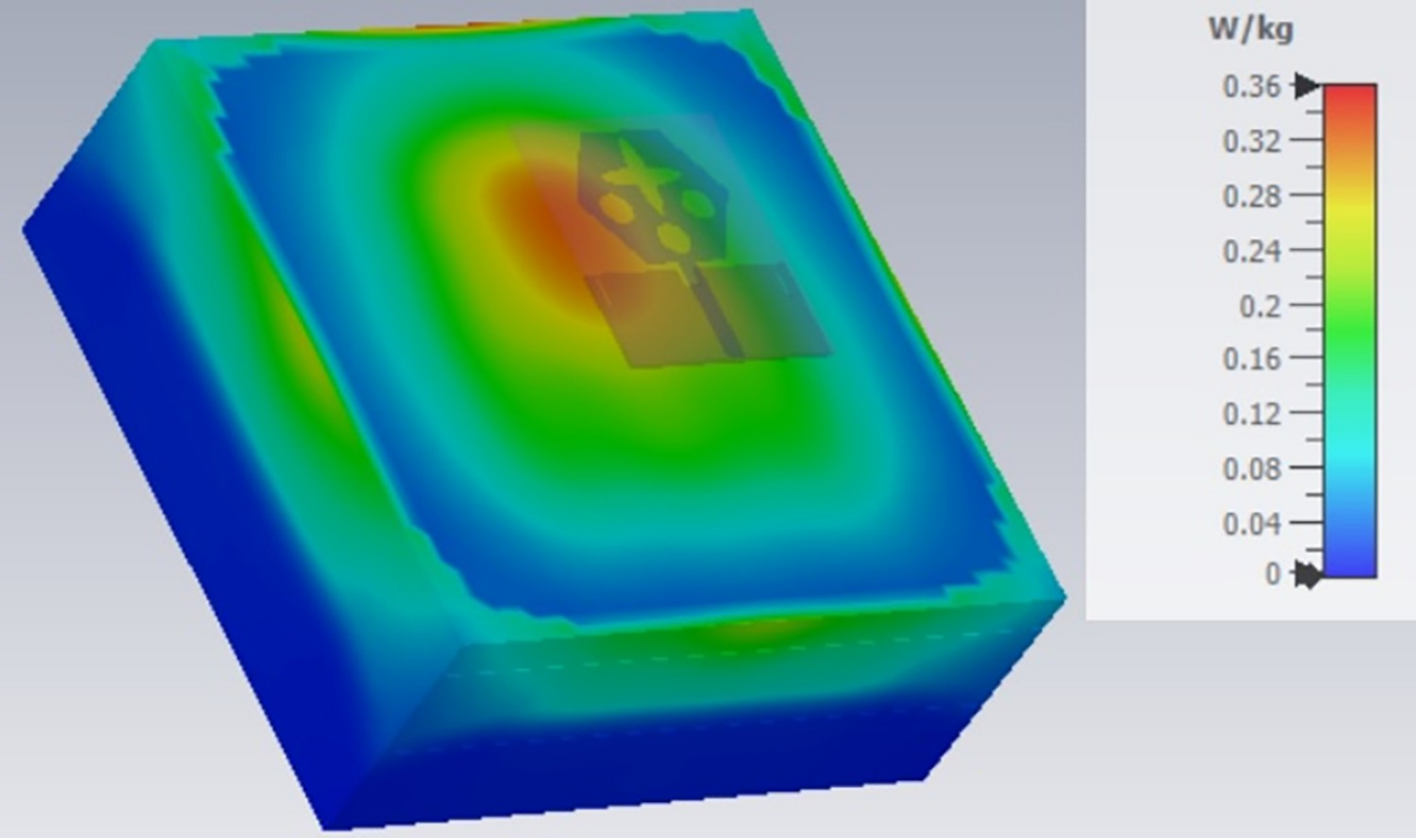
SAR analysis at 24.0GHz.

**Fig 24:**
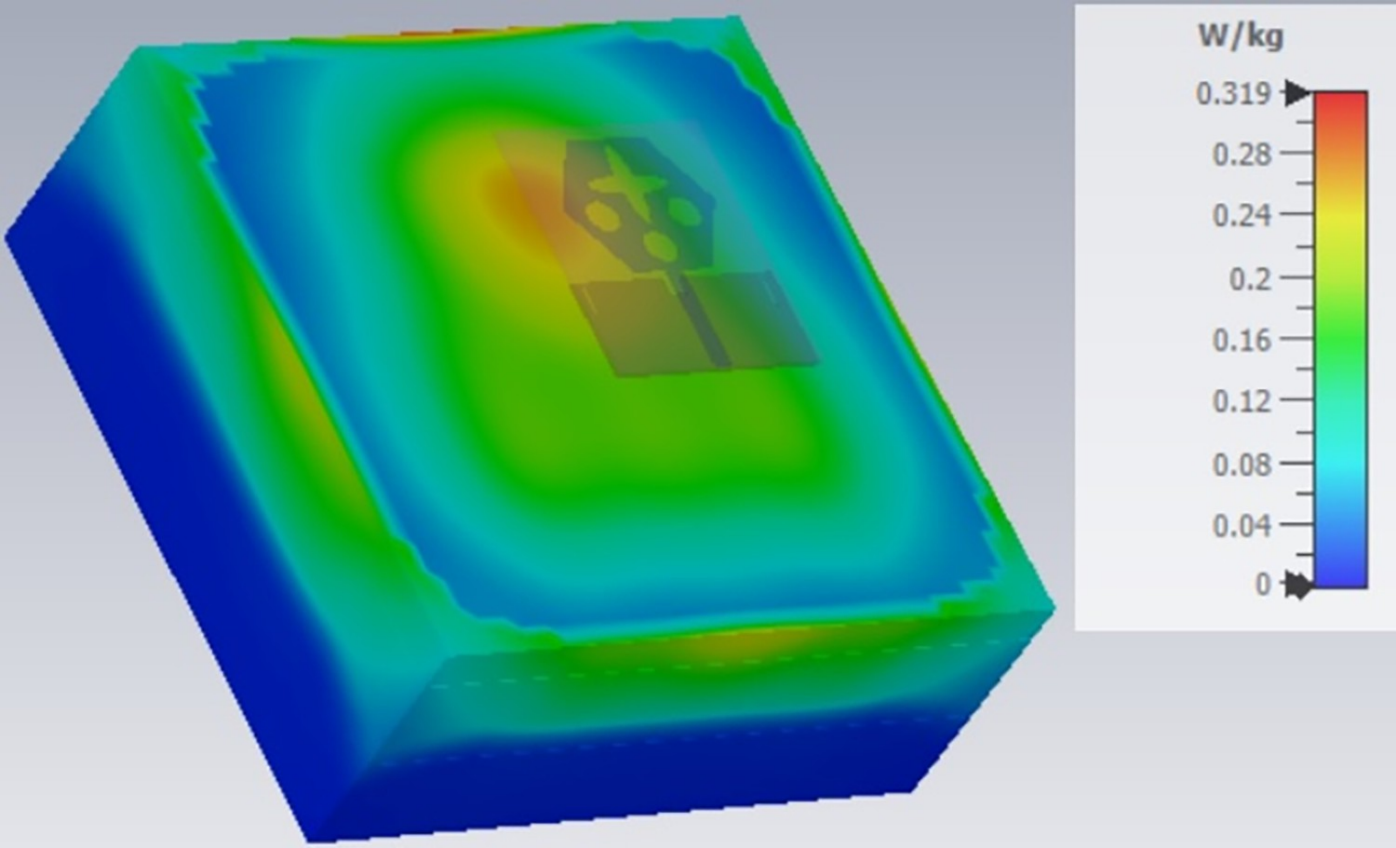
SAR analysis at 26.0GHz.

**Fig 25:**
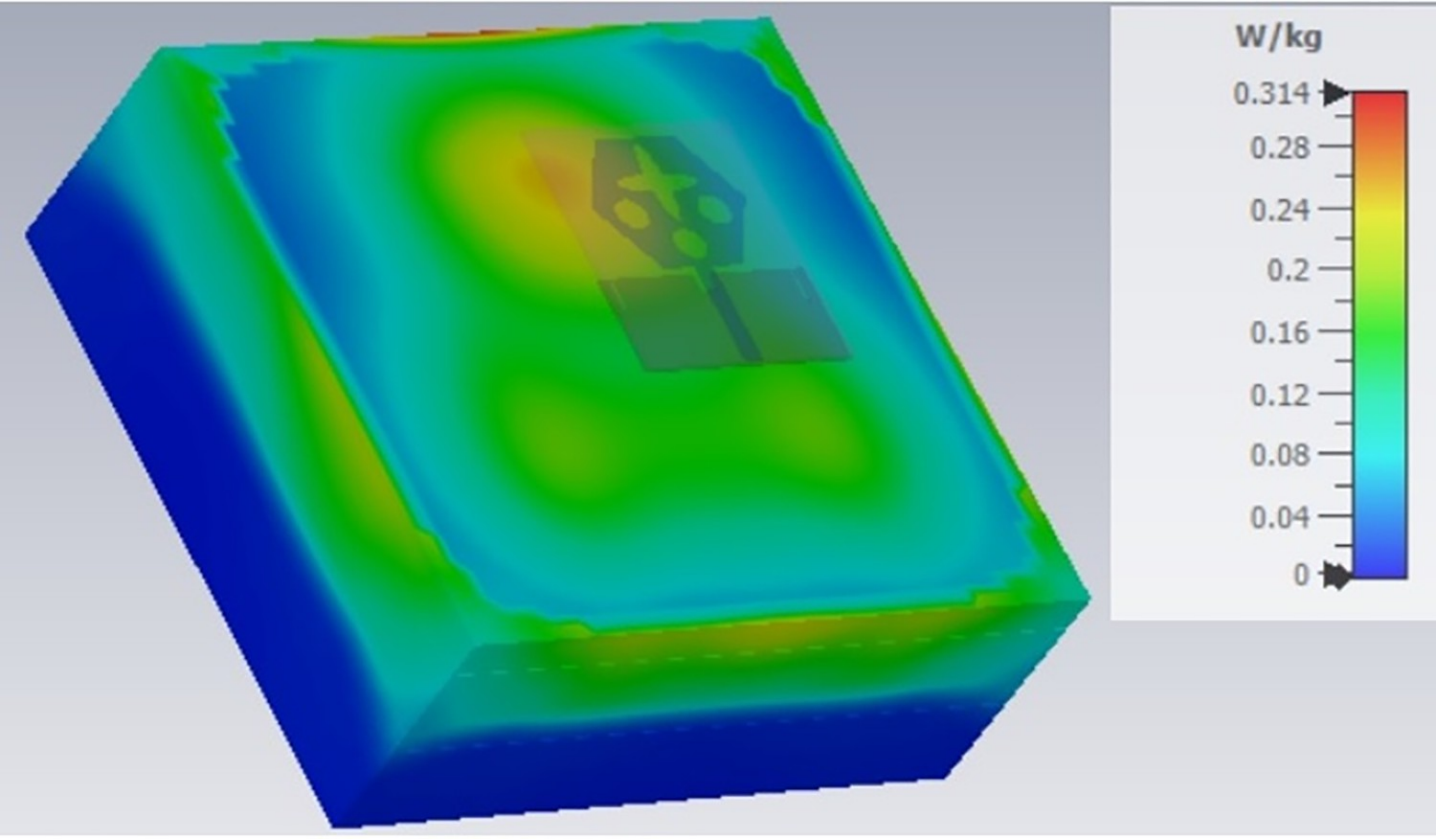
SAR analysis at 28.0GHz.

**Fig 26:**
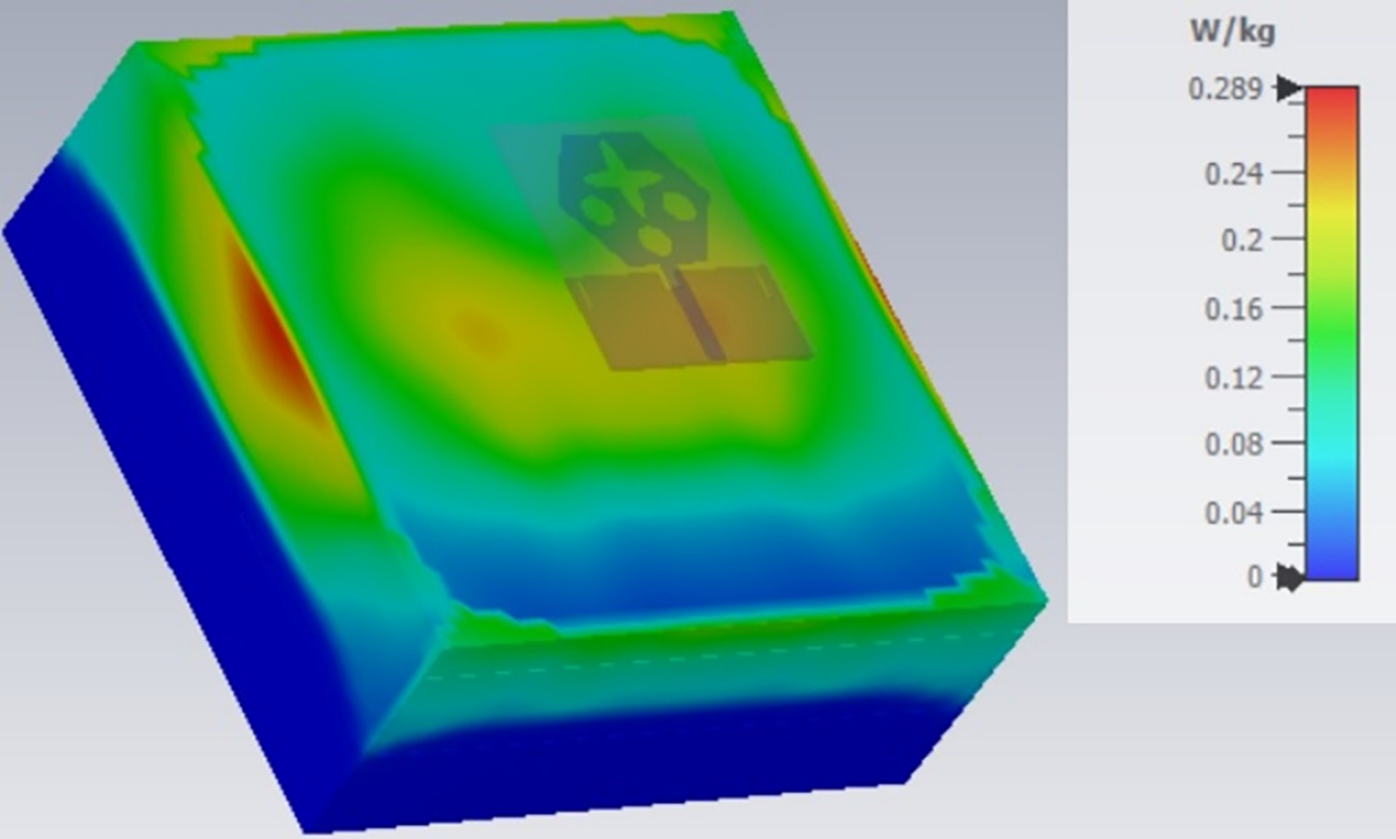
SAR analysis at 60.0GHz.

The single-port antenna with conformal configuration is also subjected to the analysis of Specific-Absorption-rate_DWMB_ (SAR_DWMB_) for on-off body applications as shown in Figs 18–26. The SAR_DWMB_ analysis is carried out by using the tissue model including skin, fat, and muscle corresponding to the thickness of 2.00mm, 5.00mm, and 5.00mm as shown in Figs 18 and 19 and is calculated by following Eq [23]

$${SAR}_{DWMB}=\int \frac{\sigma (r){\left|E(r)\right|}^{2}}{\rho (r)}dr$$ (3)

Where ***σ***- conductivity of tissue (S/m), E corresponds to electric-field-intensity_DWMB_ (V/m) and ***ρ*** is tissue-mass-density_DWMB_ (Kg/m^3^). The average Specific-Absorption-Rate(DWMB) should be less than 1.60 W/Kg for the entire operational bandwidth of 1g of the phantom-human tissue. Fig 20 shows the side-view with muscle, fat, and skin-forming tissue placed on one another and the proposed single-port antenna placed above the tissue model with a distance of 5.00mm between them. Figs 21–26 shows the plot of SAR_DWMB_ at operating frequencies of 10.90GHz, 15.0GHz, 24.0GHz, 28.0GHz and 60.0GHz respectively. The conformal applications can be achieved only when the substrate used is very thin and, in the proposed work, the Rogers RTDuroid 5880 substrate with a thin thickness of 0.254mm is used. This advantage of with very thin substrate and low profile is useful for on-body applications. The human tissue which is modeled in Figs 18 and 19 is used to understand the impact of electromagnetic waves when the proposed antenna is fed by the input signal and the SAR_DWMB_ values are noted at different multi-frequency. The electrical property of the tissue phantom shown in Fig 18 inherits the property such as permittivity, conductivity, and loss tangent which are reflected in Table 2.

**Table 2:** RF properties of the phantom-human-tissue.

Tissue_MB_	Permittivity_MB_ (ε_r(MB)_)	Conductivity_MB_ (S/m)	Loss Tangent_MB_ (tanδ)
**Skin**_**MB**_	**38.75**	**1.19**	**0.31**
**Fat**_**MB**_	**5.35**	**0.072**	**0.15**
**Muscle**_**MB**_	**53.55**	**1.35**	**0.26**

The SAR_DWMB_ analysis at different frequency points shown in Figs 21–26 is tabulated in Table 3 given below

**Table 3:** SAR_DWMB_ values.

Frequency_DWMB_ (GHz)	Maximum	Max. SAR_DWMB_	Operational Bands (GHz)
	SAR_DWMB_ Value(s)	(W/Kg)	
	(W/Kg)		
**10.90**	**0.979**	**<1.60**	**X-band**_**DB**_
**15.0**	**0.396**		**Ku band**_**DB**_
**24.0**	**0.360**		**K-band**_**DB**_
**28.0**	**0.319**		**5G-mmWave**
			**FR2**_**DB**_
			**K-band**
**60.0**	**0.314**		**5G-mmWave**
			**FR2**_**DB**_

Table 3 shows the SAR_DWMB_ values obtained by subjecting the tissue model beneath the antenna and the values vary between 0.314W/Kg to 0.979W/Kg within the operating bandwidth. The values are also well within the permissible limit of 1.60W/Kg.

### 2.4. Equivalent circuit model analysis

**Fig 27:**
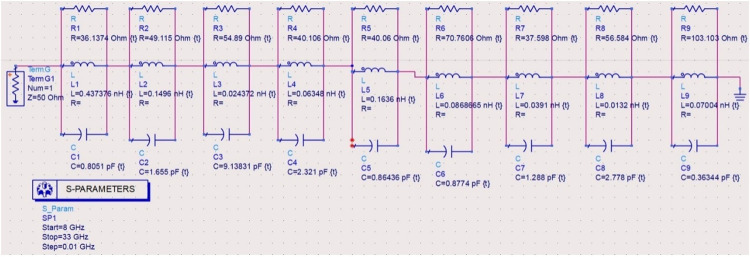
Equivalent circuit model for Band1 (8.73GHz-33.05GHz).

**Fig 28:**
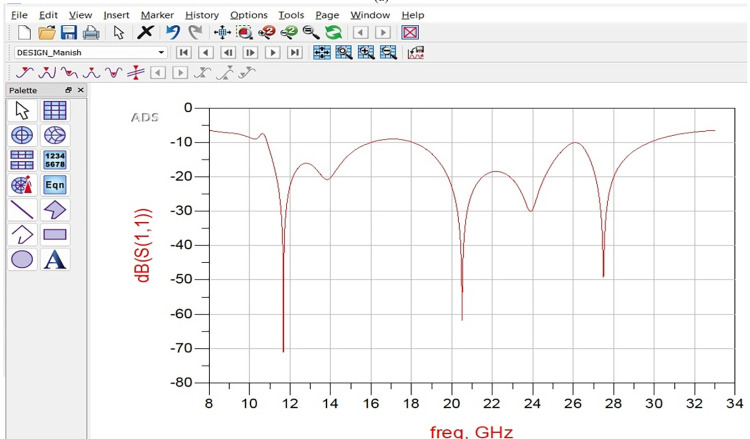
S_11_ (Band1).

**Fig 29:**
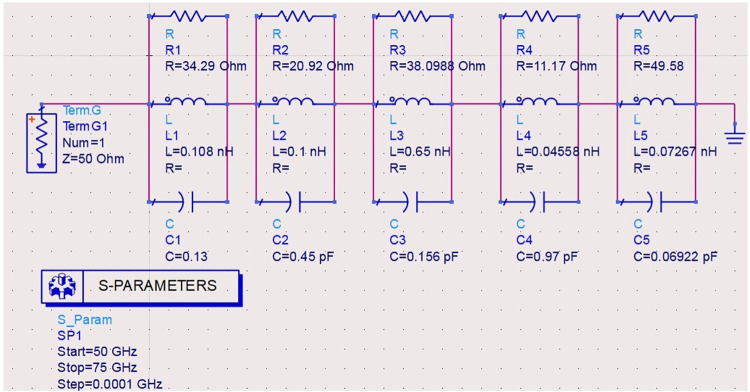
Equivalent circuit model for Band2 (52.46GHz-71.09GHz).

**Fig 30:**
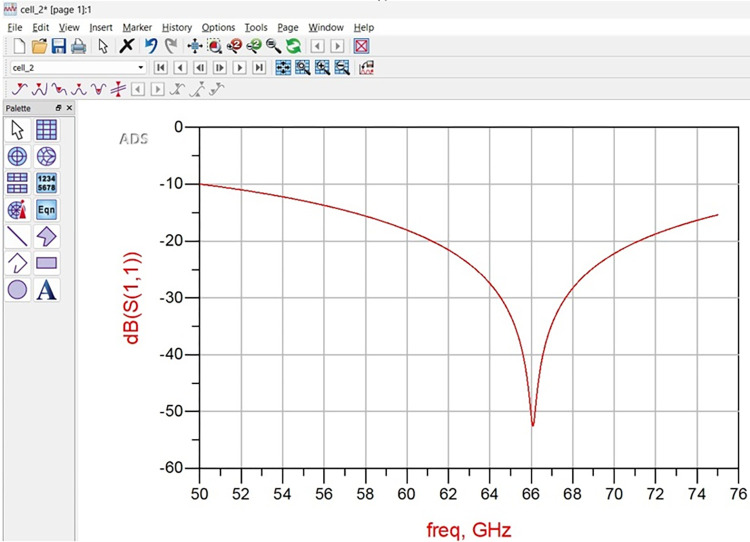
S_11_ (Band2).

**Fig 31:**
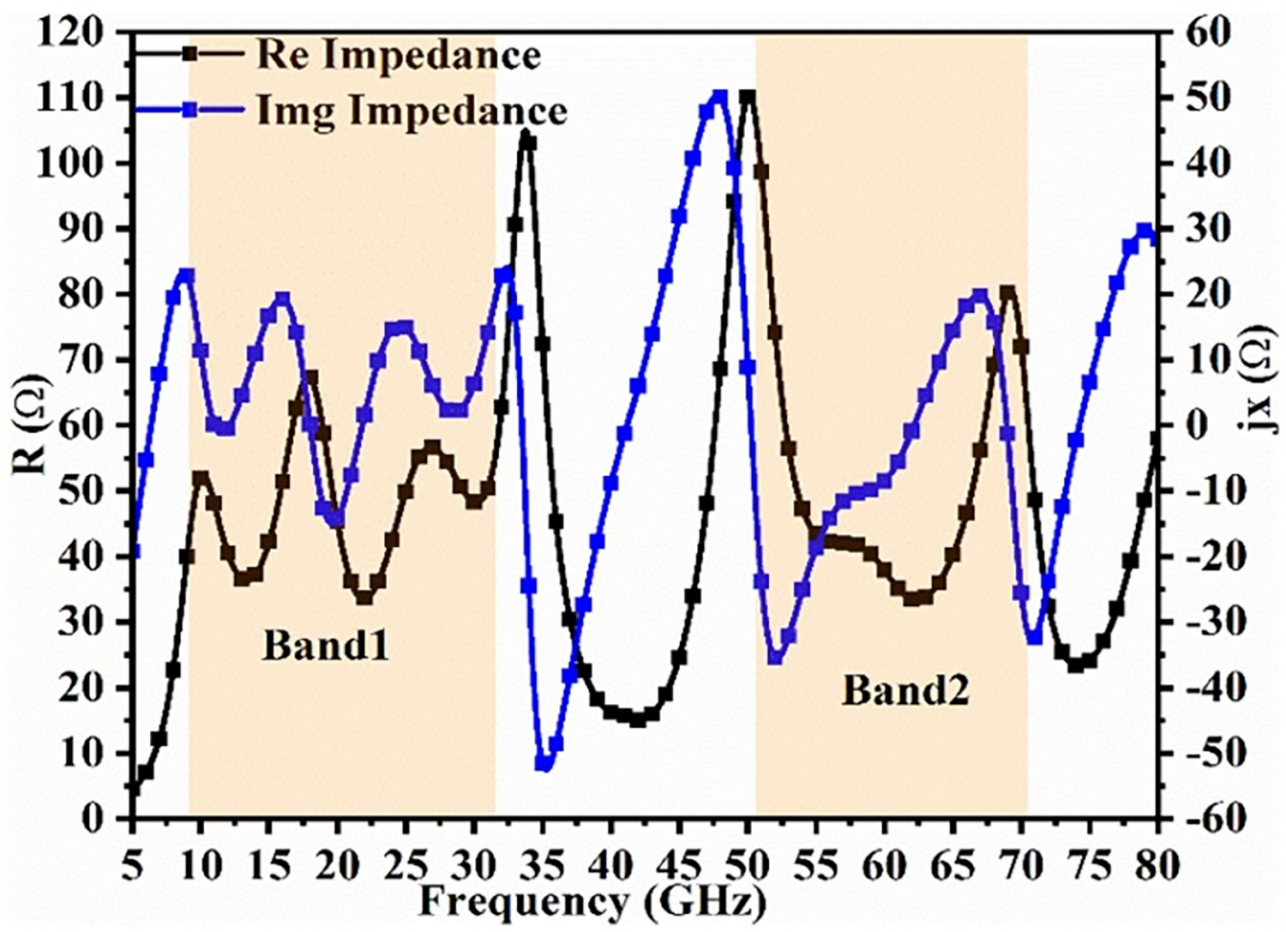
Re-Img. graph.

Figs 27–31 illustrates the analysis of the proposed single-port antenna by an equivalent circuit model. The two bands correspond to 8.73GHz-33.05GHz (Band-1) and 52.46GHz-71.09GHz (Band-2). The equivalent circuit model is developed for each band separately, as shown in Figs 27–31. Figs 27 and 28 corresponds to ECM for Band-1 with the corresponding S-parameter plot in Fig 28. The ECM for Band-1 and Band-2 can be considered as [52] the parallel RLC circuit connected in series. For the complete Band-1 (8.73GHz-33.05GHz), an infinite number of frequency points can be considered, and each frequency value will correspond to a single RLC circuit. However, for simplicity, nine frequency values corresponding to 8.73GHz, 10.12GHz, 10.76GHz, 13.12GHz, 14.67GHz, 18.69GHz, 22.44GHz, 28.80GHz, and 33.05GHz are chosen. For each frequency value, a parallel connected RLC circuit is developed in the ADS circuit simulator shown in Fig 27, and the simulated S_11_ plot is represented in Fig 28. The Band-1 occupied from Fig 28 corresponds to 11.11GHz-29.82GHz. The RLC components shown in Figs 27 and 29 are calculated from Es (4)–(6) [52] given below

$$L=\frac{img(Z_{11})}{2\pi f}$$ (4)

$$C=\frac{1}{{\left(2\pi f\right)}^{2}L}$$ (5)

$$f=\frac{1}{2\pi \sqrt{LC}}$$ (6)

The active Z-parameters are obtained from simulator and the component values are tabulated in Table 4 given below

**Table 4:** RLC values of equivalent-circuit-model.

Frequency (GHz)	R (Ω)	L (nH)	C (pF)	Bands
8.73	36.14	0.44	0.80	Band-1
10.12	49.12	0.15	1.66	
10.76	54.89	0.02	9.14	
13.12	40.11	0.06	2.32	
14.67	40.06	0.16	0.86	
18.69	70.76	0.09	0.88	
22.44	37.59	0.04	1.29	
28.80	56.84	0.01	2.78	
33.05	103.10	0.07	0.36	
52.46	34.29	0.11	0.13	Band-2
57.88	20.92	0.10	0.45	
62.44	38.10	0.65	0.156	
67.36	11.17	0.05	0.97	
71.09	49.58	0.07	0.07	

## 3. Design of dual-port_DWMB_ and four-port MIMO_DWMB_ antenna

Section II focus on single-port antenna configuration with optimal dimensions producing dual-wide bands for multiband applications. The proposed uni-port_DWMB_ antenna undergoes multiple-path fading of the received signals from different impinged angles which results in non-efficient decoding of the signals effectively. This also impacts the operating bandwidth by using multiple radiating elements at both. Transmitter and receiver, the above-said issues can overcome which can be explained by the following Shannon-Hartley Channel-Capacity Equation given below [37]

$${Ch.Cap.}_{S\times S(DWMB)}=M_{S\times S}{\Delta B}_{DWMB}\;{log}_{2}(1+\frac{Sig.}{Noi.})$$ (7)

where ***Ch*. *Cap*.**_***S***×***S***(***DWMB***)_ is the Capacity of the Channel irrespective of the MIMO_**DWMB**_ antenna (CC) given by bits/second/Hz, ***ΔB***_***DWMB***_ are the two integrated bandwidths corresponding to Band 1 & Band 2, ***M***_***S*×*S***_ is the integer-multiplication number depending on the number of radiating elements and Sig.Noi. corresponds to the ratio of signal-to-noise. For single-port, ***M***_***S*×*S***_ = 1 and for two or four-port MIMO_DWMB_ the values are 2 and 4. Thus multiple-radiating elements in the receiver ensure reception of the transmitted signals all the time and reduce the fading effects with an increase in efficiency. Further, the Shannon-capacity theorem represented by Eq (7) is modified to [37]

$${Ch.Cap.}_{S\times S(DWMB)}=M_{S\times S}\Delta B\;{log}_{2}(1+\frac{M_{S\times S}}{N_{S\times S}}\frac{Sig.}{Noi.})$$ (8)

Where ***M***_***S*×*S***_ (numerator) is the number of radiating elements at the transmitter side and ***M***_***S*×*S***_ (denominator) is the number of antenna elements on the receiver side. Eq (8) correlates the MIMO configuration deployed at the receiver and the transmitter side with assumptions that multiple-path channels are un-correlated or assumed ideal environment. This also concludes that designing of MIMO antenna with a very low correlation between them becomes more vital and de-coupling element/structure or orientation of the radiating elements becomes more critical to achieve higher data rates of transmission and reception.

**Fig 32:**
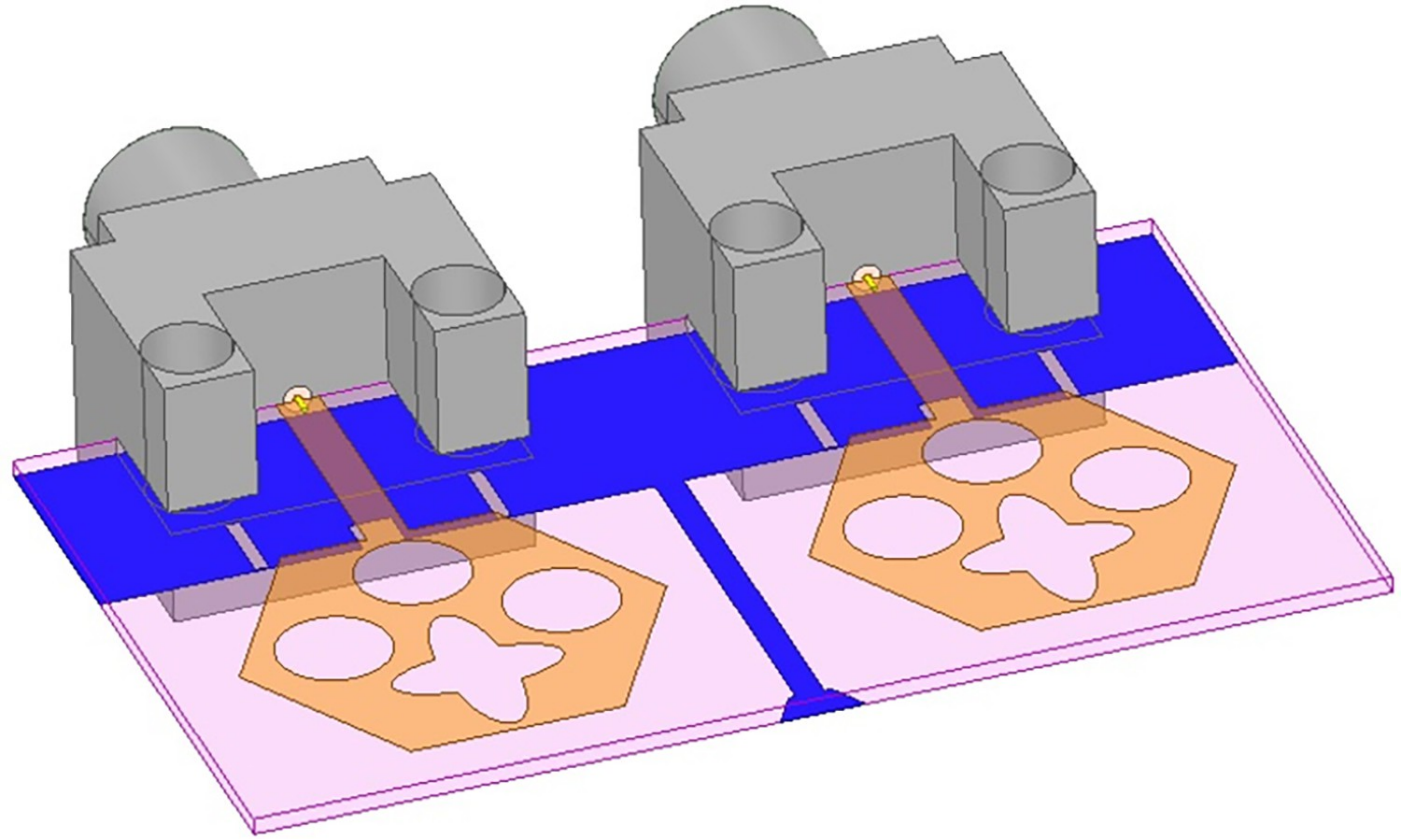
Dual-port MIMO_DWMB_ antenna configuration Isometric view.

**Fig 33:**
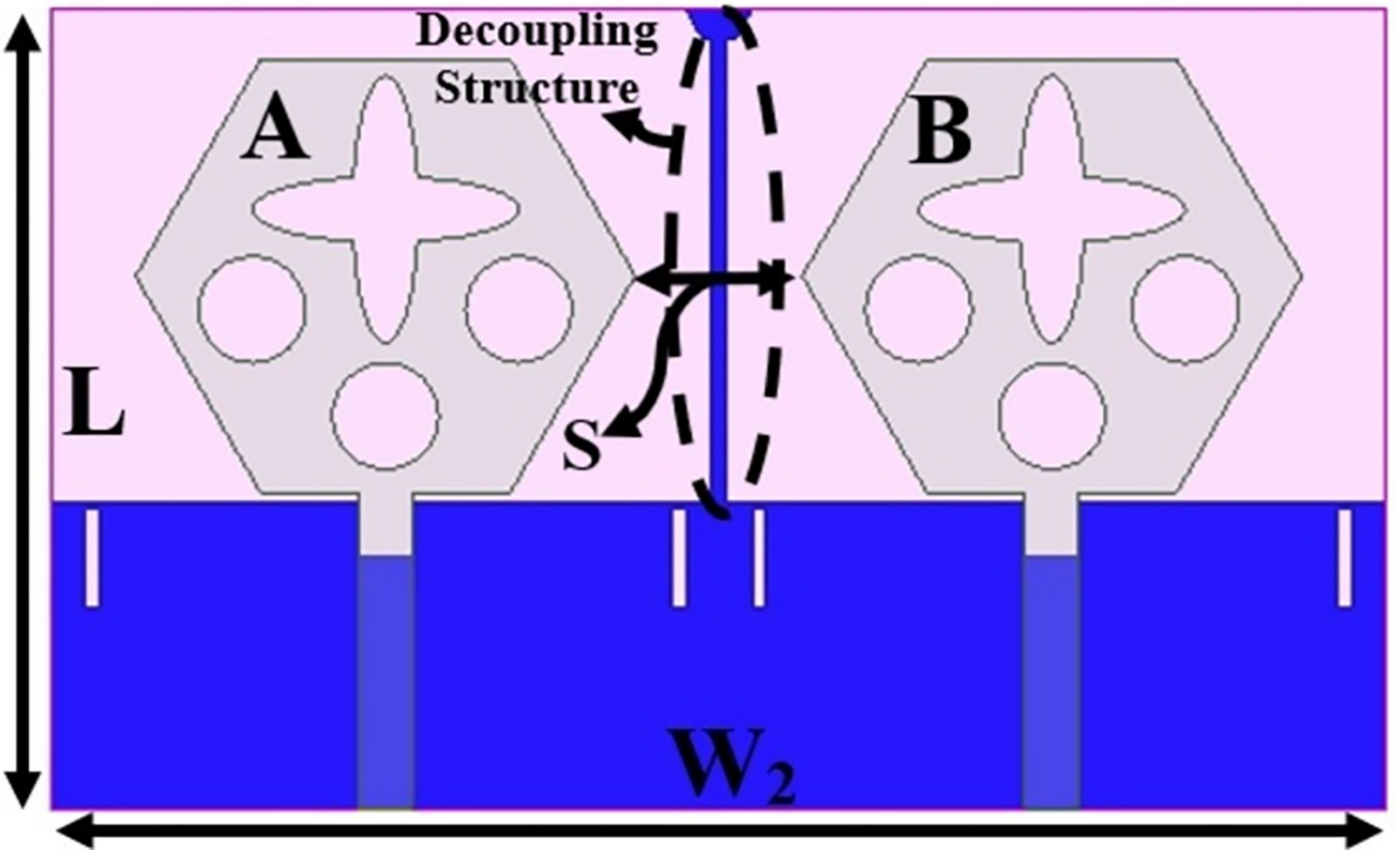
Dual-port MIMO_DWMB_ antenna configuration front-view.

**Fig 34:**
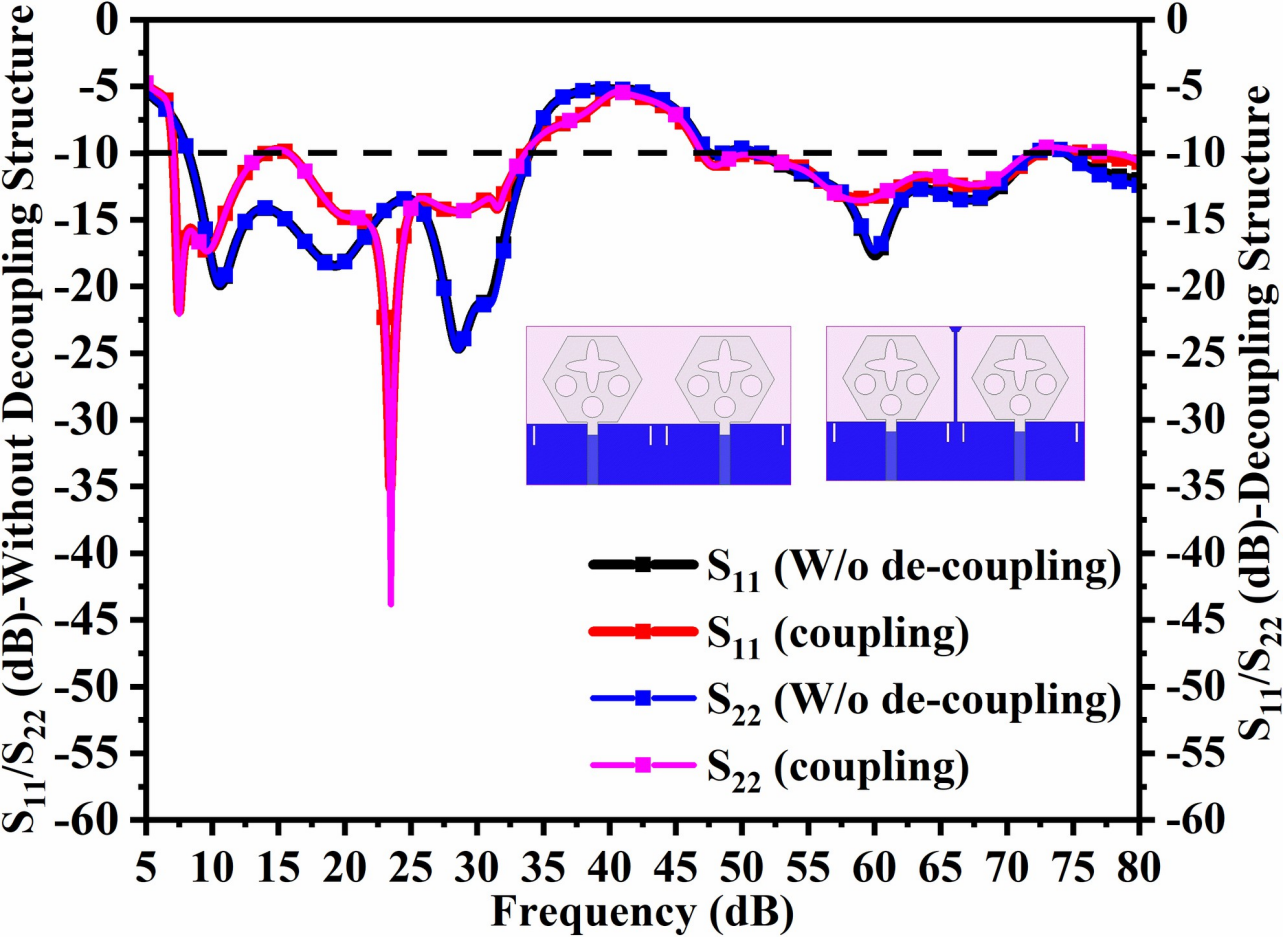
S_11_/S_22_ (without and with de-coupling structure).

**Fig 35:**
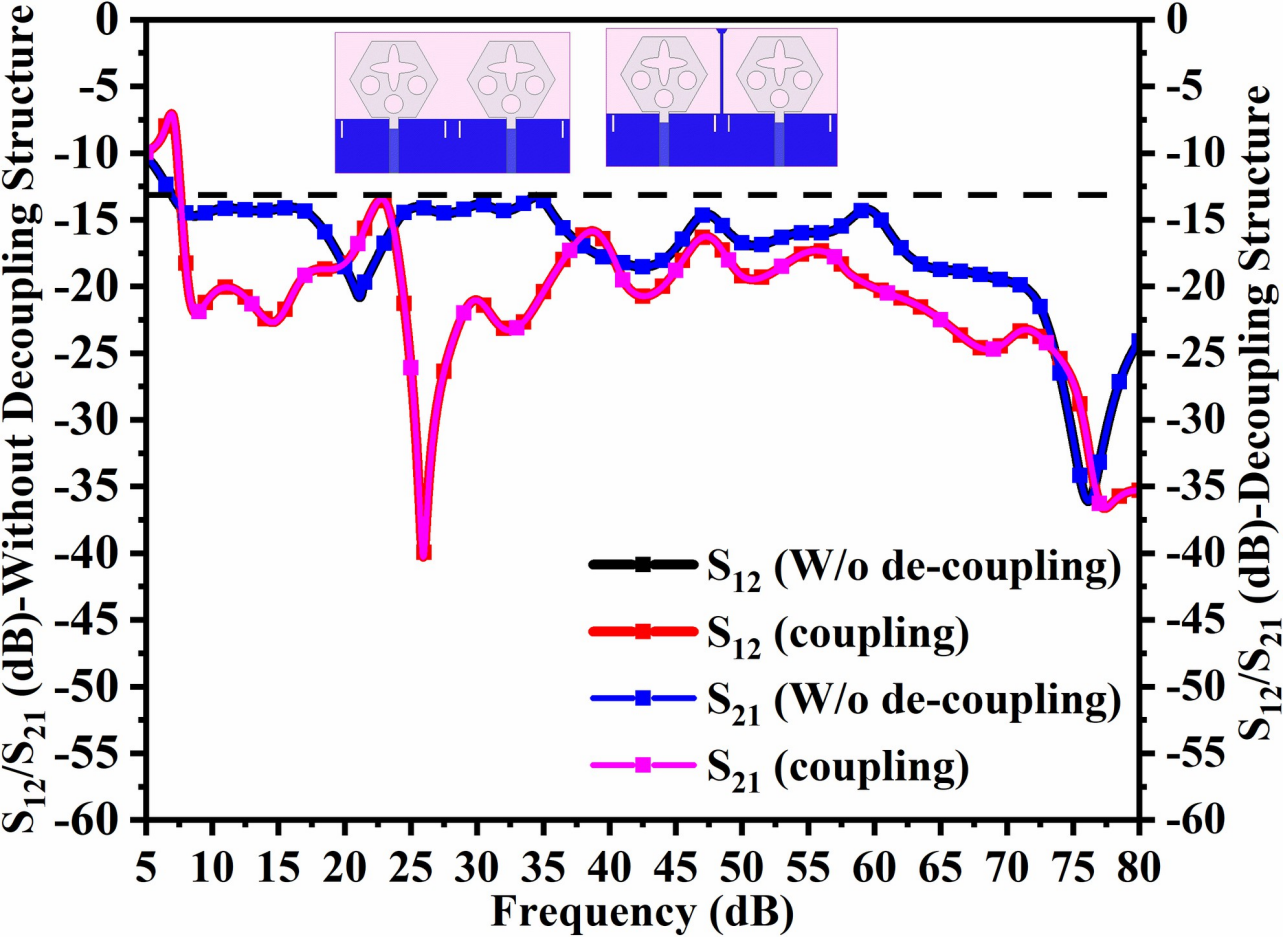
S_12_/S_21_ (without and with de-coupling structure).

**Fig 36:**
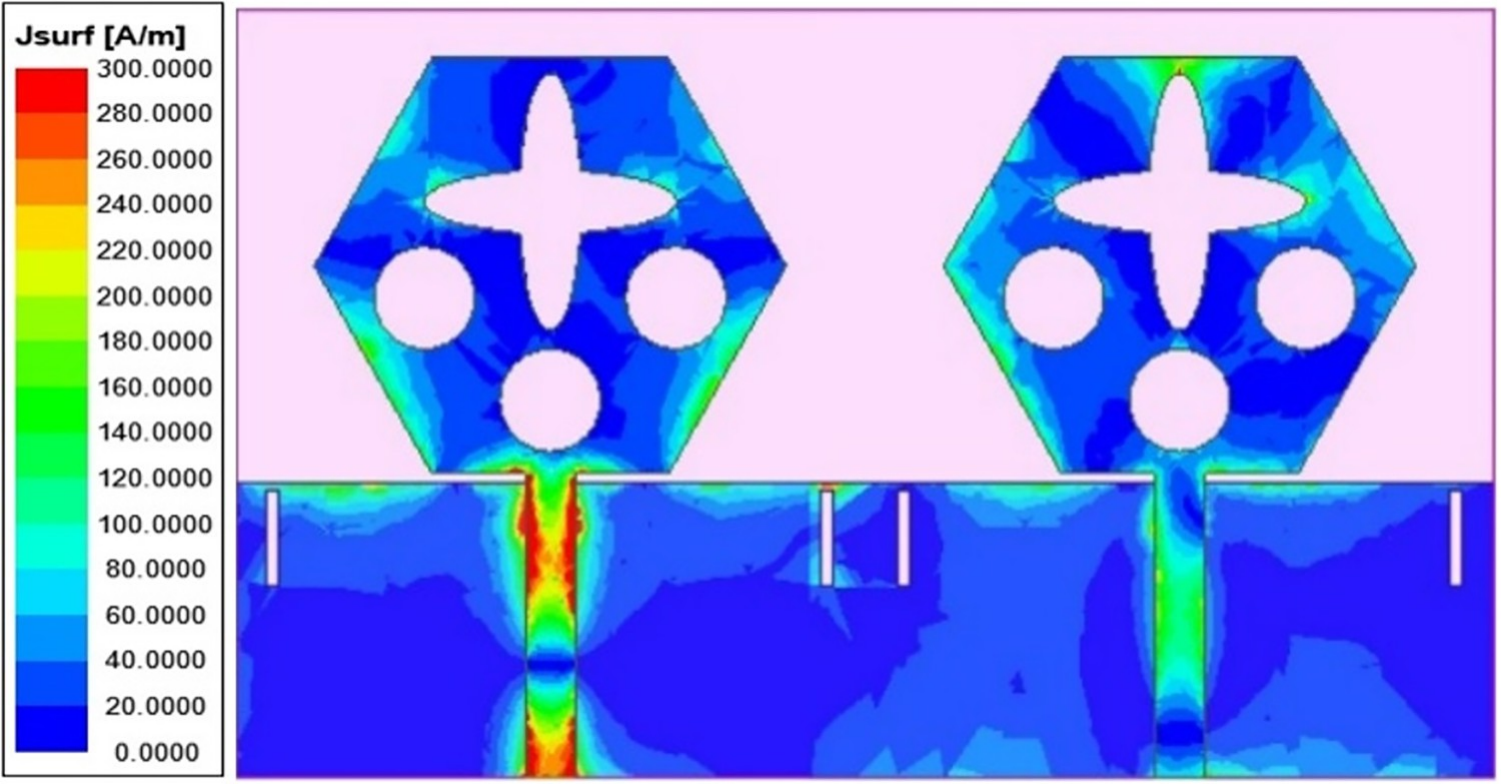
SFD_DWMB_ without de-coupling structure.

**Fig 37:**
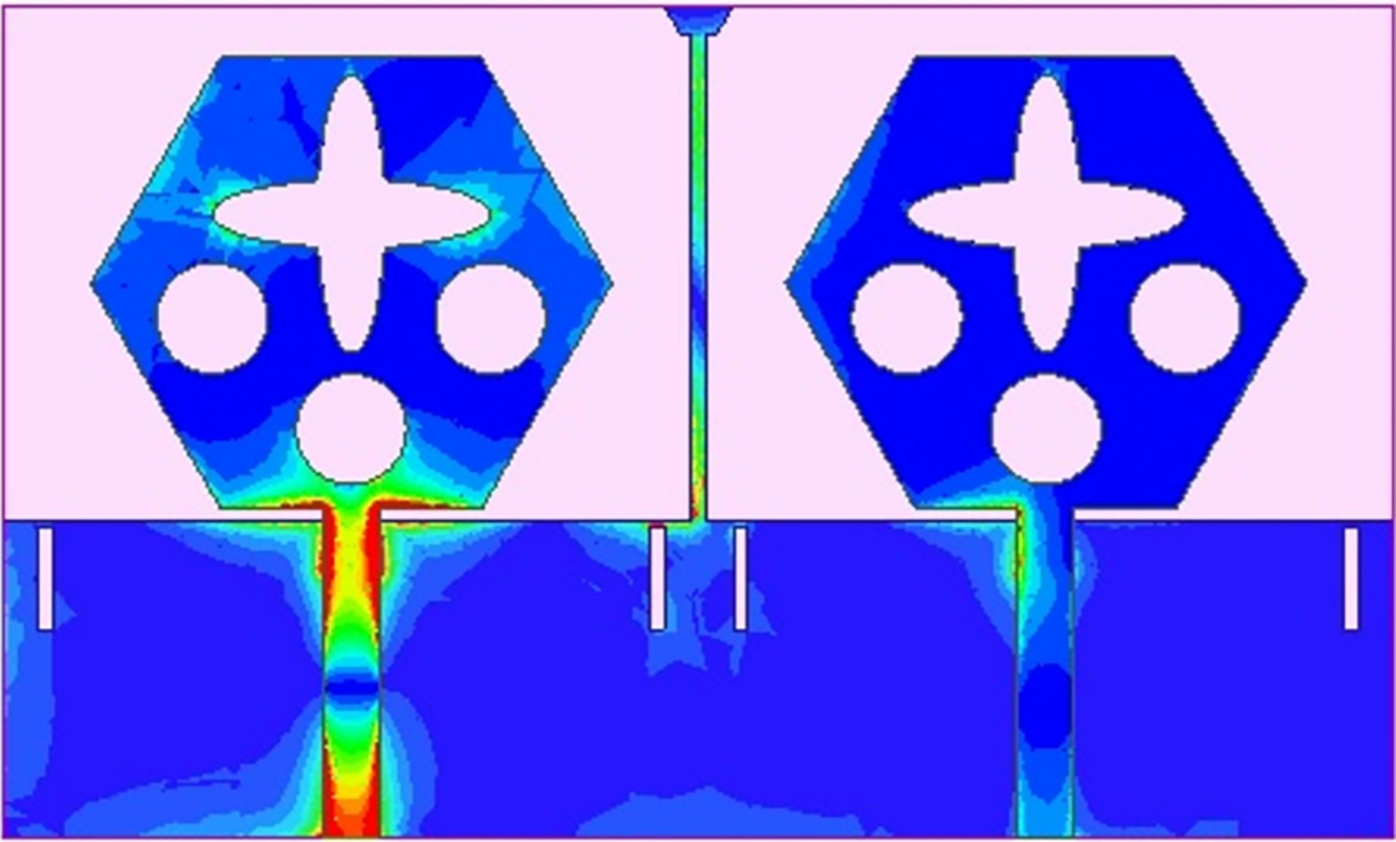
SFD_DWMB_ with de-coupling structure.

**Fig 38:**
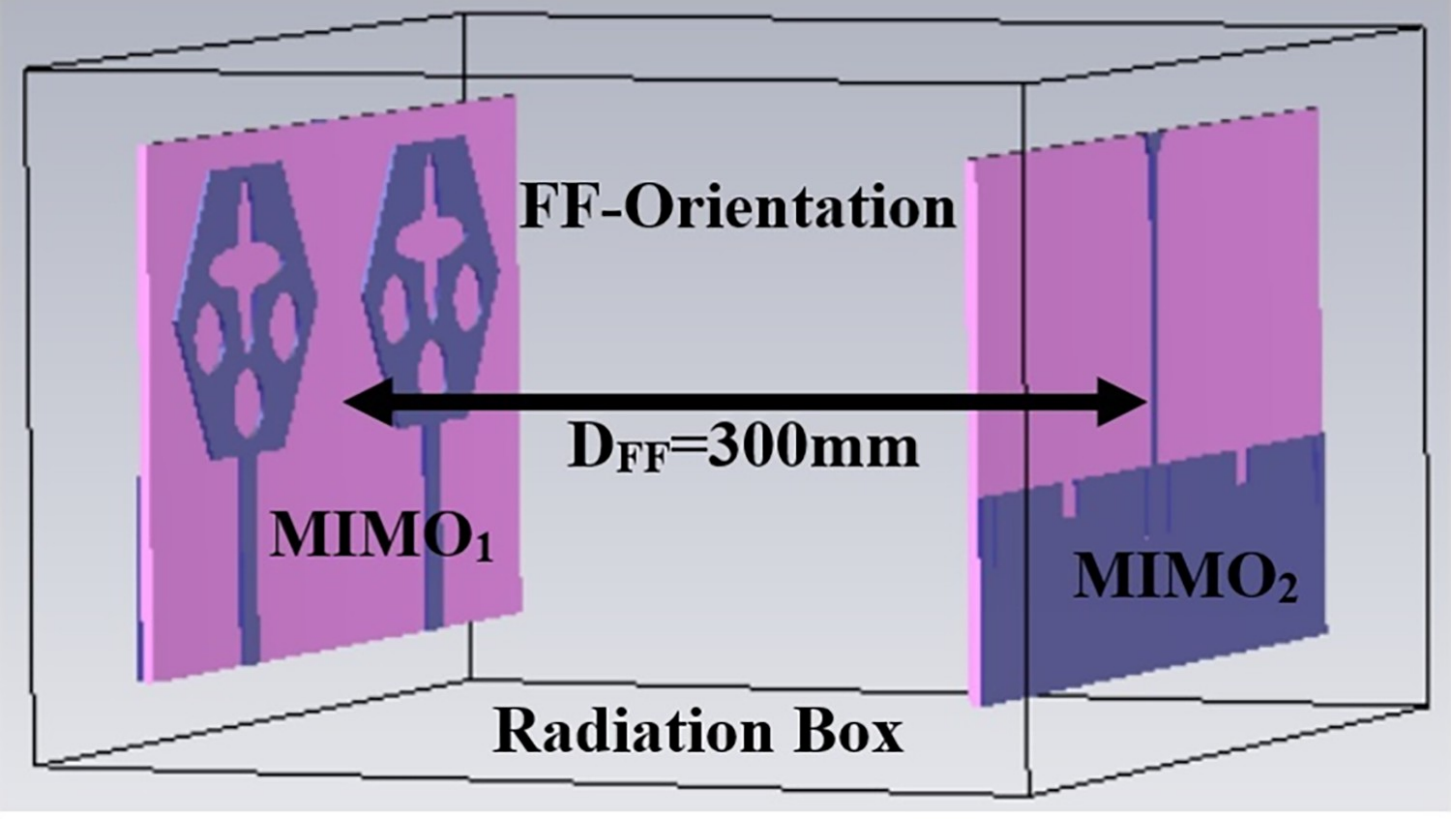
Time-domain analysis in face-to-face orientation.

**Fig 39:**
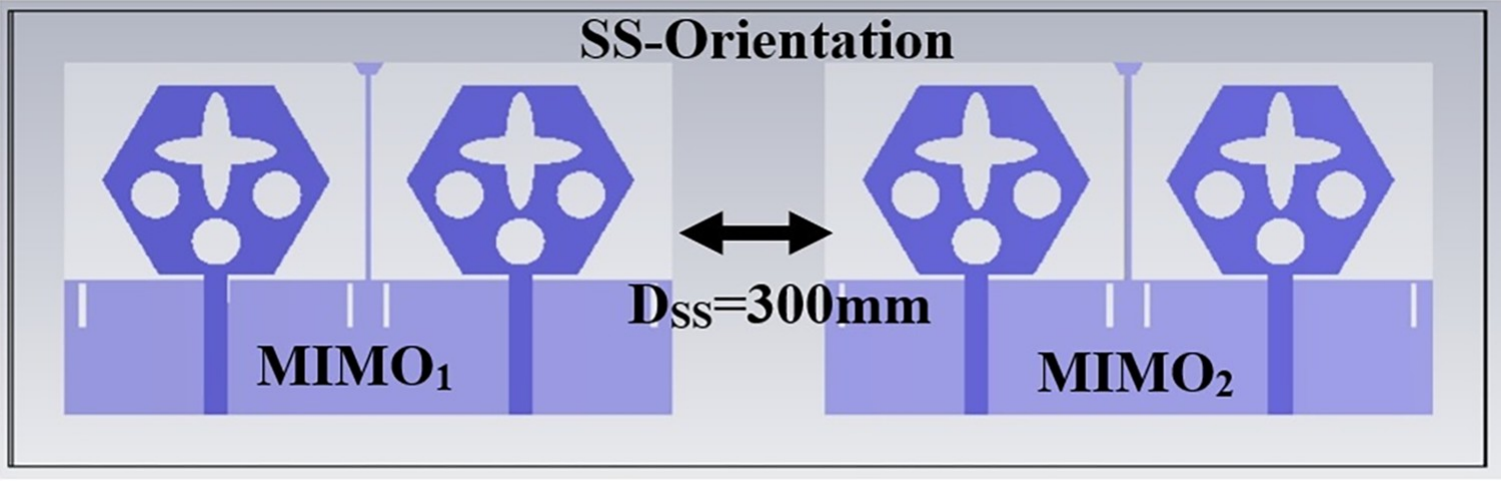
Time-domain analysis in side-to-side orientation.

Figs 32 and 33 shows the configuration of the proposed two-port MIMO_DWMB_ antenna which corresponds to representing the perspective and front views. The two-identical radiating elements are placed adjacent to each other and share a common ground. The feed line is connected with a Jhonson SMK1.85mm Screw-on type plug connector for input signal transmission. The proposed MIMO_DWMB_ antenna occupies a total area of L×W_2_ mm^2^ and the spacing of **S** mm is maintained. The de-coupling structure is designed with the integration of a rectangular stub and half-hexagon attached to the ground. Fig 34 shows the S_11_/S_22_ result for both, without (W/o) de-coupling and with de-coupling structure. In the former case, the proposed MIMO_DWMB_ antenna offers S_11_/S_22_ impedance-bandwidth of 8.18GHz-33.93GHz for Band-1 & 47.50GHz-72.37GHz for Band-2 and with embedded de-coupling structure, the impedance bandwidth corresponds to 7.05GHz-33.67GHz & 46.95GHz-72.47GHz. Fig 35 shows the comparison of the isolation achieved without and with de-coupling structure which shows the improvement of isolation with decoupling structure as it provides the additional current path entering the neighboring radiating element. Figs 36 and 37 represents the simulation of surface-current density over the surface of the patch and ground at the designed frequency. The absence of de-coupling structure in Fig 36 shows that the surface-current density with feeding of port-1 effects the other patch and hence reduces the isolation. However, the de-coupling structure attached to the ground as observed from Fig 37, acts as the current path and thereby, blocks the flow of current density to the adjacent radiator. This effect can be understood from Fig 35 which shows the plot of transmission coefficients S_12_/S_21._ The absence of a de-coupling structure records the isolation between 13.0dB to 20.0dB but, improves with the addition of a de-coupling structure and averages around 25.0dB in both the operating bands. Further, due to the wider impedance bandwidth offered in both the bands of the proposed antenna, it becomes essential to understand the shape of the pulse received by the receiver in the far-field region concerning the transmitted pulse. Figs 38 and 39 shows the two orientations used to study the impulse response and plot of group delay concerning frequency. Fig 38 shows the orientation of Face-to-face (FF) with a distance of D_FF_ = 300mm between both the two-port MIMO antenna configurations. Fig 39 corresponds to Side-to-Side (SS) orientation with edge spacing of Dss = 300mm. These two orientations ensure the reception of the signal in all the possible orientations. The far-field condition is achieved by following the equation given below [37]

$$Far\;Field\;region>\frac{2\times w_{2}^{2}}{\lambda_{designed\;frequency}}$$ (9)

where ***W***_**2**_ = 20 mm is the length (larger dimension) of the antenna and ***λ***_***designed frequency***_ is the center frequency.


$$\phi_{DWMB}=-\frac{d\theta (\omega )}{d\omega }$$ (10)

$$\rho_{DWMB}={max}_{\phi }\left[\frac{\int S_{Tx}(t)S_{Tx}(t-\phi )dt}{\sqrt{S_{Tx}^{2}(t)dt}\sqrt{S_{Rx}^{2}(t)dt}}\right]$$ (11)

**Fig 40:**
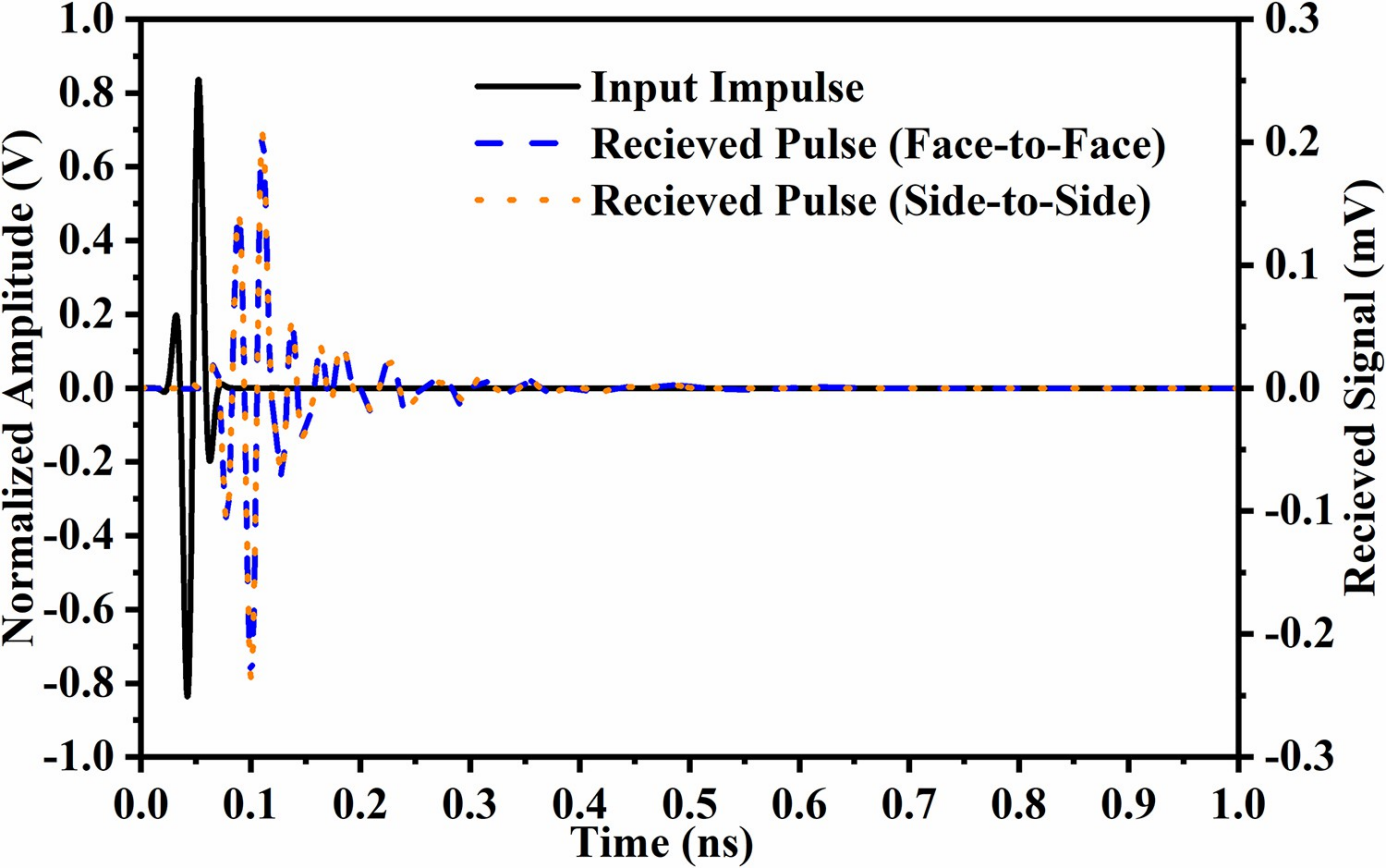
Impulse-response_DWMB_.

The time-domain analysis for wide-band antennas becomes vital so that the transmitted pulse can be analyzed when received by the receiver. This is an important analysis as it concludes the transmission of the modulated signal will have low or high distortion at the receiver. This analysis is understood by subjecting the proposed two-port_DWMB_ antenna to time-domain analysis and studying impulse response and group delay evaluated by Eqs (10)–(11). Fig 40 corresponds to the impulse response where the short-sinusoidal pulse is fed to the transmitter. The two orientations are used, one when Tx-Rx is placed face-to-face (FF) and the other side-to-side (SS). The short-sinusoidal transmitted pulse is effectively received in both the said orientations with 180° phase difference, reduced amplitude, and ripples. However, the amplitude or strength of the received signal is enhanced by using an amplifier and the ripples are filtered.

**Fig 41:**
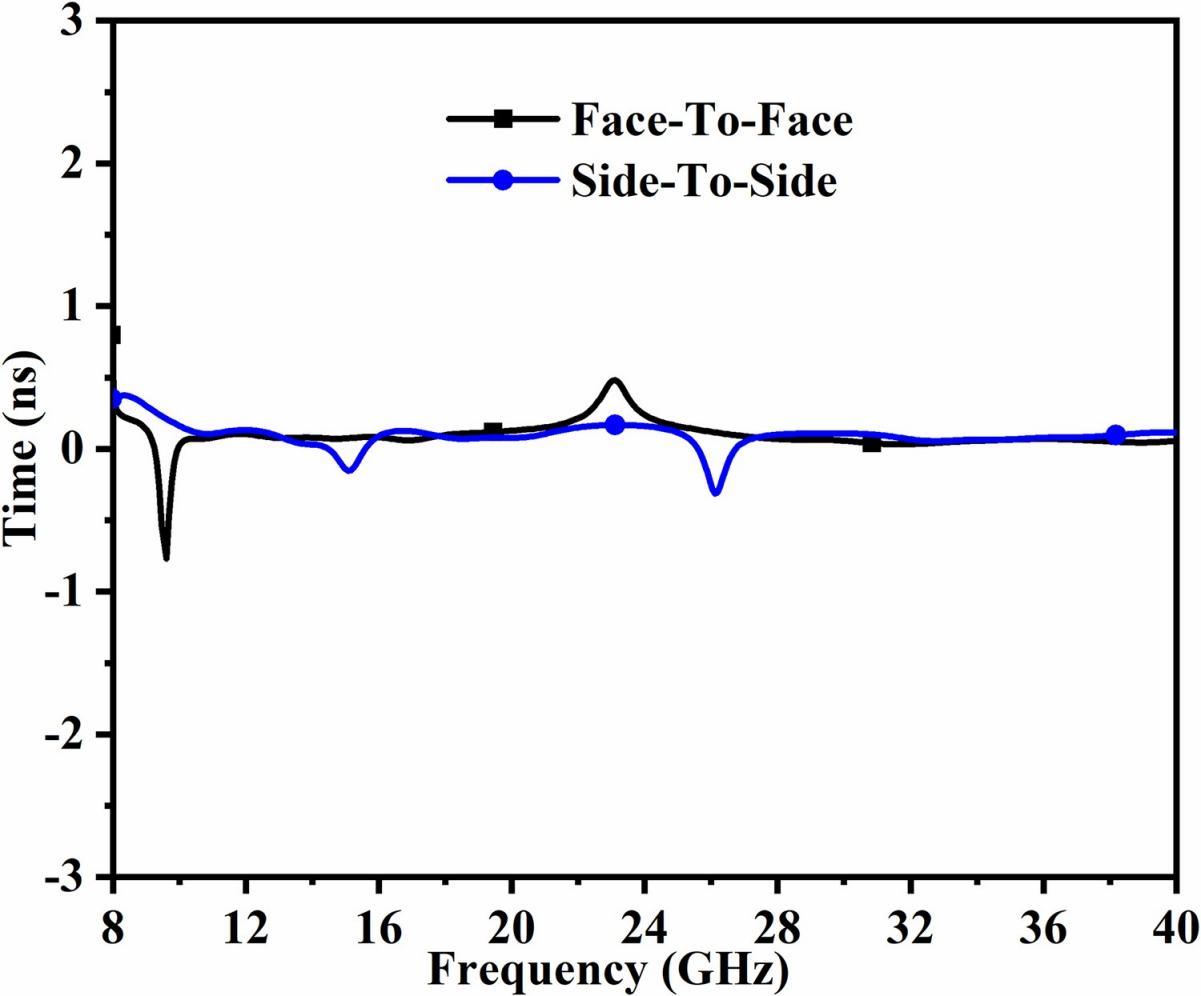
Group-Delay_DWMB_ (FF and SS).

The group delay corresponds to a delay when the group-signal travels within the linear-system of network. The group-delay is sometimes also referred as envelope-delay due to the propagation time of the transmitted envelope. Fig 41 corresponds to calculated group-delay for FF/SS orientations with maximum time-variation between ±0.3ns. This value of the delay do not produce much distortion and the proposed 2-port antenna network can be used effectively.

## 4. Four-port MIMODWMB antenna with commonly connected ground

**Fig 42:**
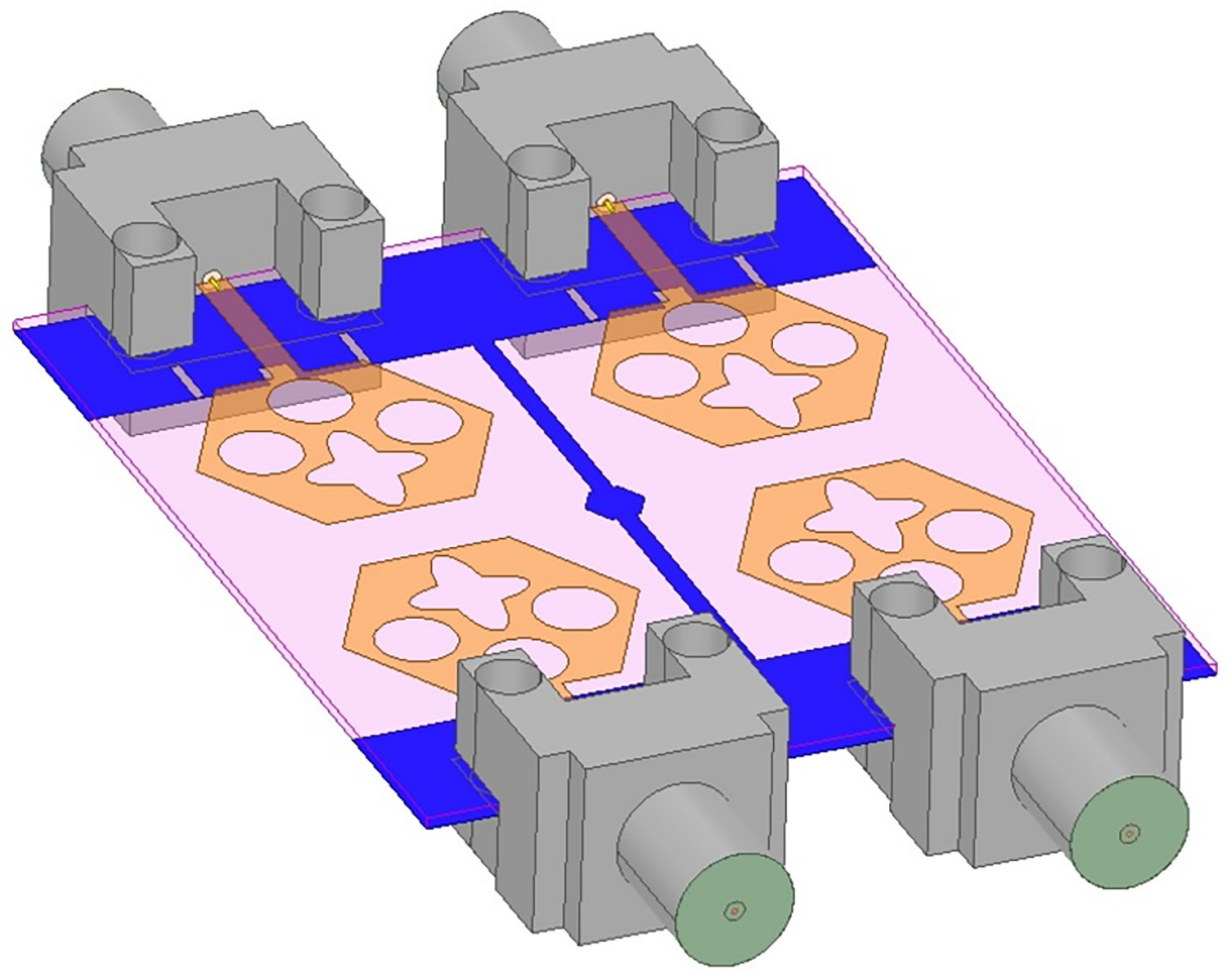
Four-Port MIMO_DWMB_ antenna with perspective view.

**Fig 43:**
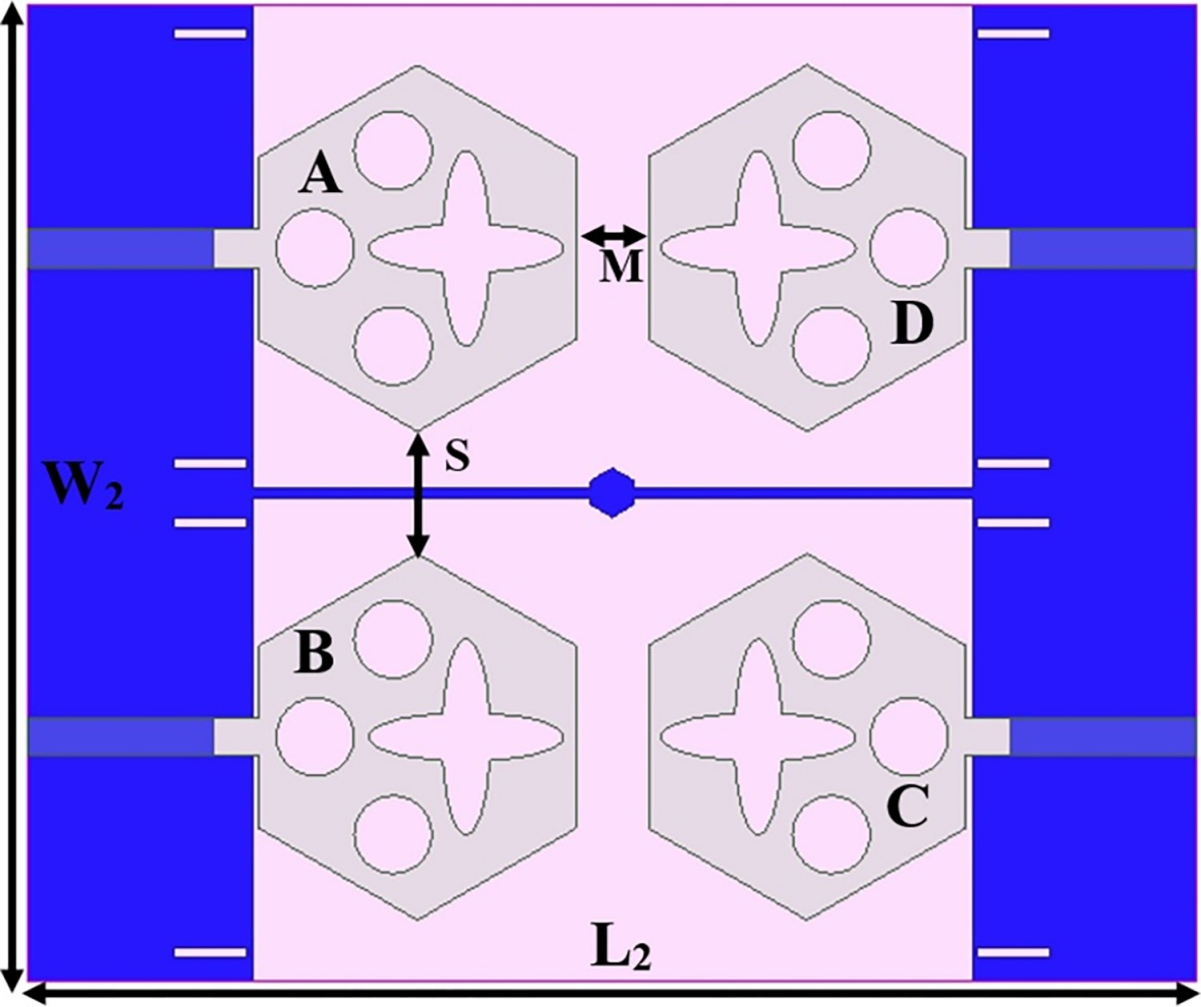
Four-Port MIMO_DWMB_ antenna with front-ground view.

**Fig 44:**
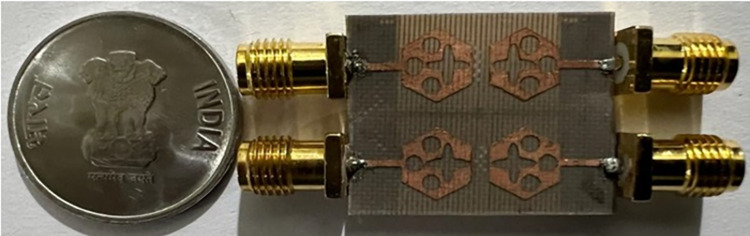
Four-Port MIMO_DWMB_ antenna prototype front-view.

**Fig 45:**
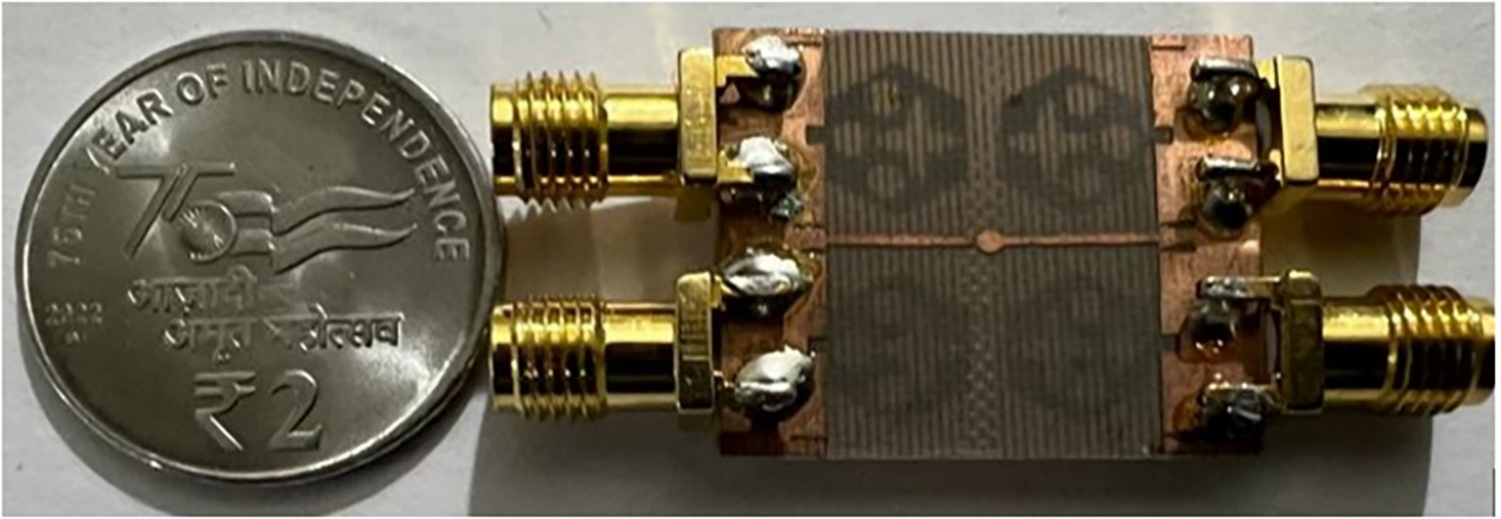
Four-Port MIMO_DWMB_ antenna prototype ground-view.

The not-connected grounds or separate ground is a very common mistake and needs to be rectified. The separate ground for each radiating-element in the MIMO_DWMB_ antenna configuration provides direct port isolation and this is due to the no-direct coupling through the ground as it is not continuous. The split in the ground in real-system applications is not practically accepted and hence, the common ground becomes essential in multi-port MIMO_DWMB_ antenna configuration. In keeping view of the above problems and providing common ground, the four-port MIMO_DWMB_ antenna is developed which is an enhanced version of the proposed two-port MIMO_DWMB_ antenna discussed in Section 3. Figs 42–45 illustrates the designed four-port MIMO_DWMB_ antenna with more capable of transferring data at higher rates with a lesser bit error rate. The two-port MIMO_DWMB_ antenna shown in Figs 32–35 is converted to a four-port by placing identical two-port antenna pairs at 180° orientation and the respective partial-ground connected by de-coupling by de-coupling structure and the four connectors are used to excite the radiating patch. The four radiating antennas are marked as A, B, C, and D with Antenna A and Antenna B placed adjacent to each other with common-shared ground. Also, Antenna C and Antenna D are replicas of Antenna A & Antenna B but placed 180° orientation as shown in Figs 42 and 43. The distance of **S** mm is maintained between adjacent radiating elements and spacing of **M** mm between the 180° oriented antenna. Figs 44 and 45 shows the fabricated prototype-front-ground photograph with high accuracy achieved by the photolithographic method. The prototype is used to calculate measured S-parameters, gain, and the 2-D radiation patterns.

**Fig 46:**
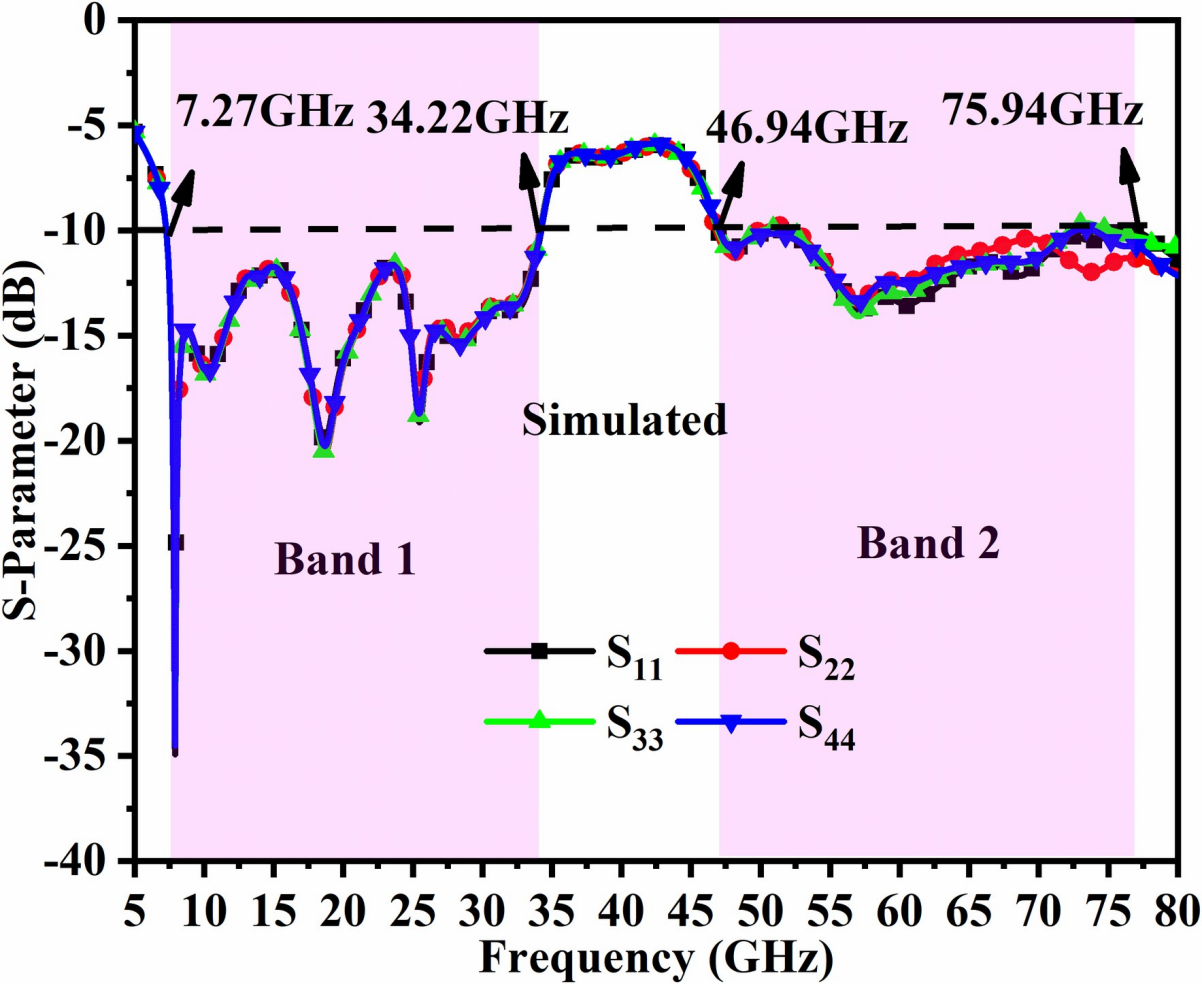
Simulated reflection-coefficients S-parameters.

**Fig 47:**
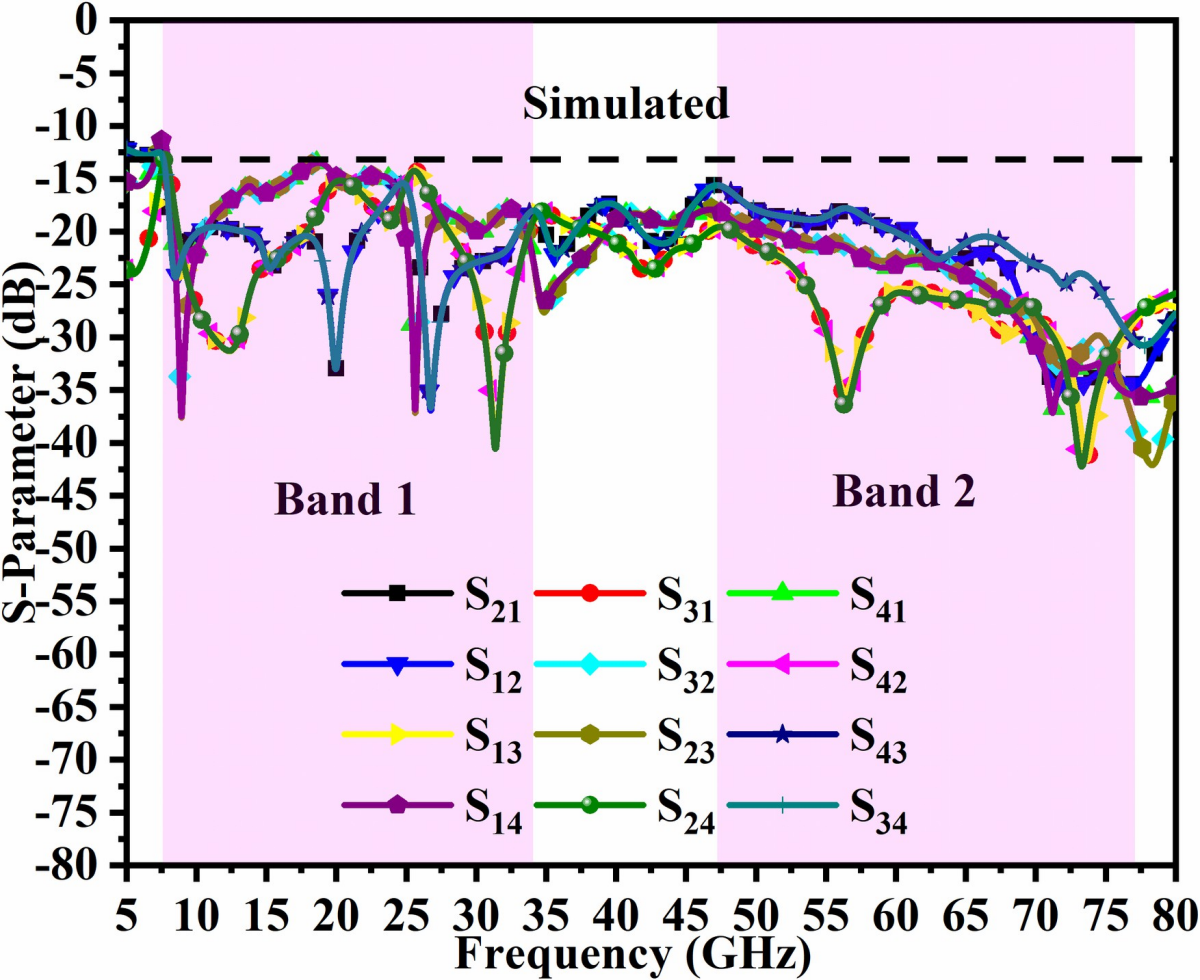
Simulated transmission-coefficients S-parameters.

**Fig 48:**
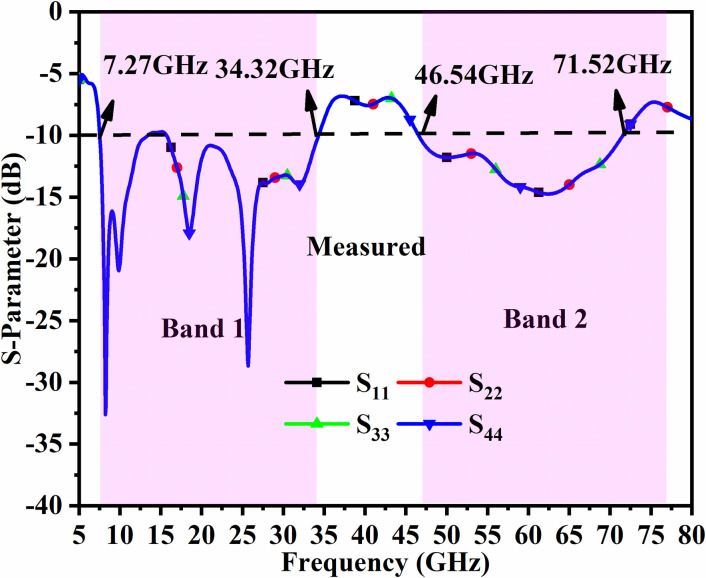
Measured reflection-coefficients S-parameters.

**Fig 49:**
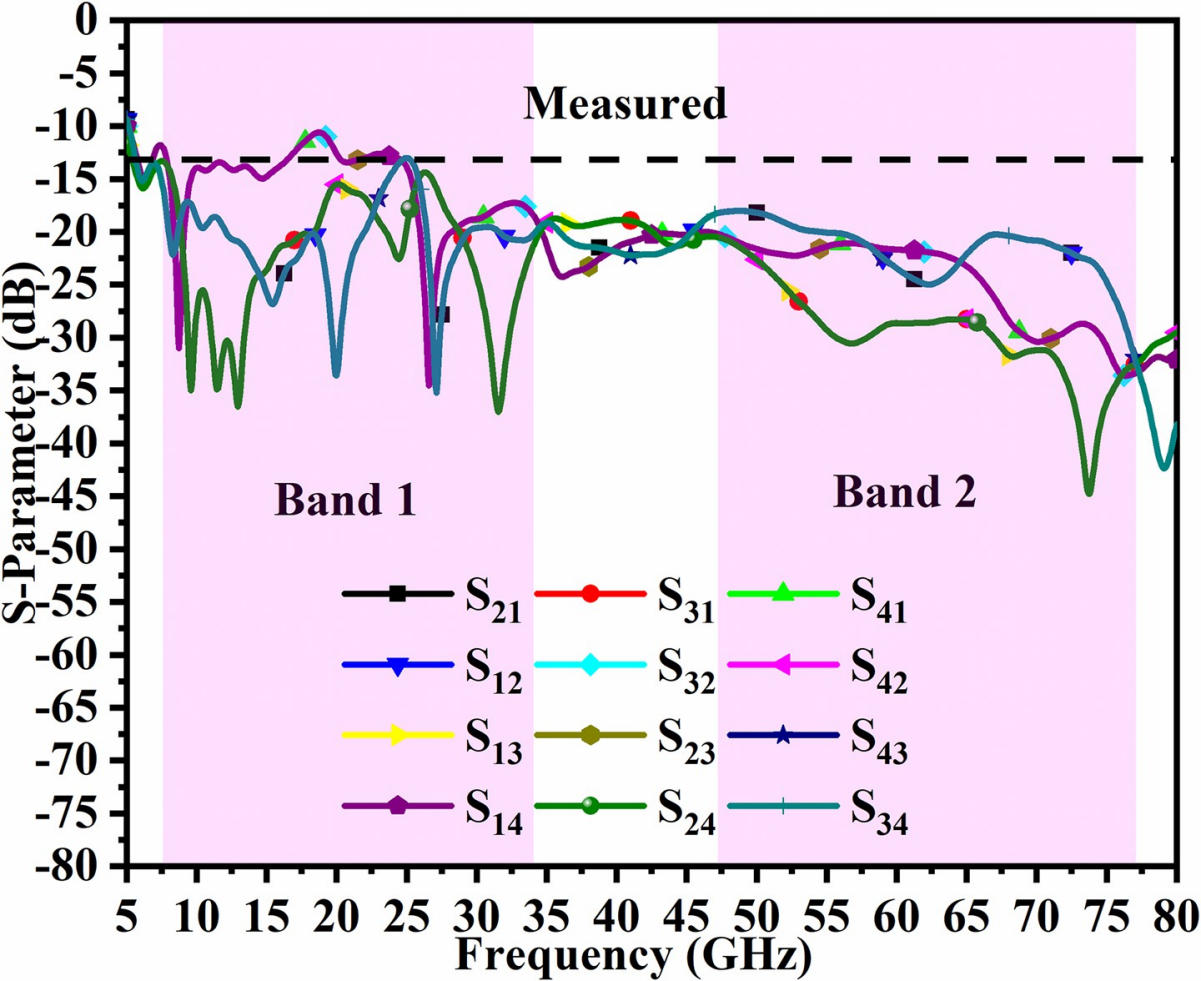
Measured transmission-coefficients S-parameters.

Figs 46–49 demonstrate the simulated and measured S-parameters (reflection and transmission coefficients). The four-port MIMO antenna with Port A_DWMB_, Port B_DWMB_, Port C_DWMB,_ and Port D_DWMB_ results in 16 S-parameters given by S-matrix (A_DWMB_ = 1, B_DWMB_ = 2, C_DWMB_ = 3, D_DWMB_ = 4)

$$\left[S_{ABCD(DWMB)}]=[\begin{array}{c} S_{(DWMB)AA}\;S_{(DWMB)AB}\;S_{(DWMB)AC}\;S_{(DWMB)AD}\\ S_{(DWMB)BA}\;S_{(DWMB)BB}\;S_{(DWMB)BC}\;S_{(DWMB)BD}\\ S_{(DWMB)CA}\;S_{(DWMB)CB}\;S_{(DWMB)CC}\;S_{(DWMB)CD}\\ S_{(DWMB)DA}\;S_{(DWMB)DB}\;S_{(DWMB)DC}\;S_{(DWMB)DD} \end{array}\right]$$ (12)

The reflection coefficients correspond to S_(DWMB)AA_ = S_11_, S_(DWMB)BB_ = S_22_, S_(DWMB)CC_ = S_33,_ and S_(DWMB)DD_ = S_44_. The transmission-coefficients include S_(DWMB)AB_ = S_12_, S_(DWMB)AC_ = S_13_, S_(DWMB)AD_ = S_14_, S_(DWMB)BA_ = S_21_, S_(DWMB)BC_ = S_23_, S_(DWMB)BD_ = S_24_, S_(DWMB)CA_ = S_31_, S_(DWMB)CB_ = S_32_, S_(DWMB)CD_ = S_34_, S_(DWMB)DA_ = S_41_, S_(DWMB)DB_ = S_42_ and S_(DWMB)DC_ = S_43_. The simulated -10.0dB bandwidth corresponds to 7.27GHz-34.22GHz for Band 1 and 46.94GHz-75.94GHz for Band 2. The measured -10.0dB values are 7.27GHz-34.32GHz and 60.0GHz bandwidth of 46.54GHz-71.52GHz corresponding to Band 1 and Band 2 as noted from Figs 46 and 47. Figs 47 and 49 corresponds to isolation for transmission coefficients S_(DWMB)21_, S_(DWMB)31_, S_(DWMB)41_, S_(DWMB)12_, S_(DWMB)32_, S_(DWMB)42_, S_(DWMB)31_, S_(DWMB)23_, S_(DWMB)43_, S_(DWMB)14_, S_(DWMB)24_ and S_(DWMB)34_ in simulation and measured conditions. The band 7.25GHz-8.30GHz is used for satellite Downlink/Uplink communication, the applications (8.175GHz-8.215GHz) in X-band corresponds to satellites for the earth exploration, meteorological satellites for monitoring weather conditions (8.00GHz-12.0GHz), The Ku-band (12.0GHz-18.0GHz) is used in very-small-aperture-terminal (VSAT) communication, K-band (18GHz-27.0GHz) are used in radar and satellite applications with infrared domain is useful in astronomical study and the K-band (26.50GHz-40.0GHz) is used in applications such as close-rang targeting RADARS, military aircraft, space telescopes, wireless point-to-point microwave communication and vehicle speed detection systems. All the above said bands falls in Band1 (7.27GHz-34.32GHz). The Band2 bandwidth corresponding to 46.54GHz-71.52GHz find application in millimeter-Wave bands corresponding to n262 (47.20GHz-48.20GHz) used in uplink 5G (47.20GHz-48.20GHz) and downlink (47.20GHz-48.20GHz) with channel bandwidths of 50MHz, 100MHz, 200MHz and 400MHz. The 60.0GHz (n263: 47.0GHz-71.0GHz) is used in 5G New-Radio (5G-NR) applications. Due to the ease of design, the microstrip feed MIMO_DWMB_ is easily integrated with PCB by placing the antenna on the edge or the side-frame.


$$\gamma_{c}=\frac{\int_{0}^{2\pi }\int_{0}^{\pi }(\left({XPRE}_{\theta .m}(\theta ,\phi )E_{\theta ,s}^{*}(\theta ,\phi )P_{\theta }(\theta ,\phi )+E_{\phi .m}(\theta ,\phi )E_{\phi ,s}^{*}(\theta ,\phi )P_{\phi }(\theta ,\phi \right))sin\theta \;d\theta \;d\phi }{\sqrt{\gamma_{d}^{2}}\sqrt{\gamma_{s}^{2}}}$$ (13)

**Fig 50:**
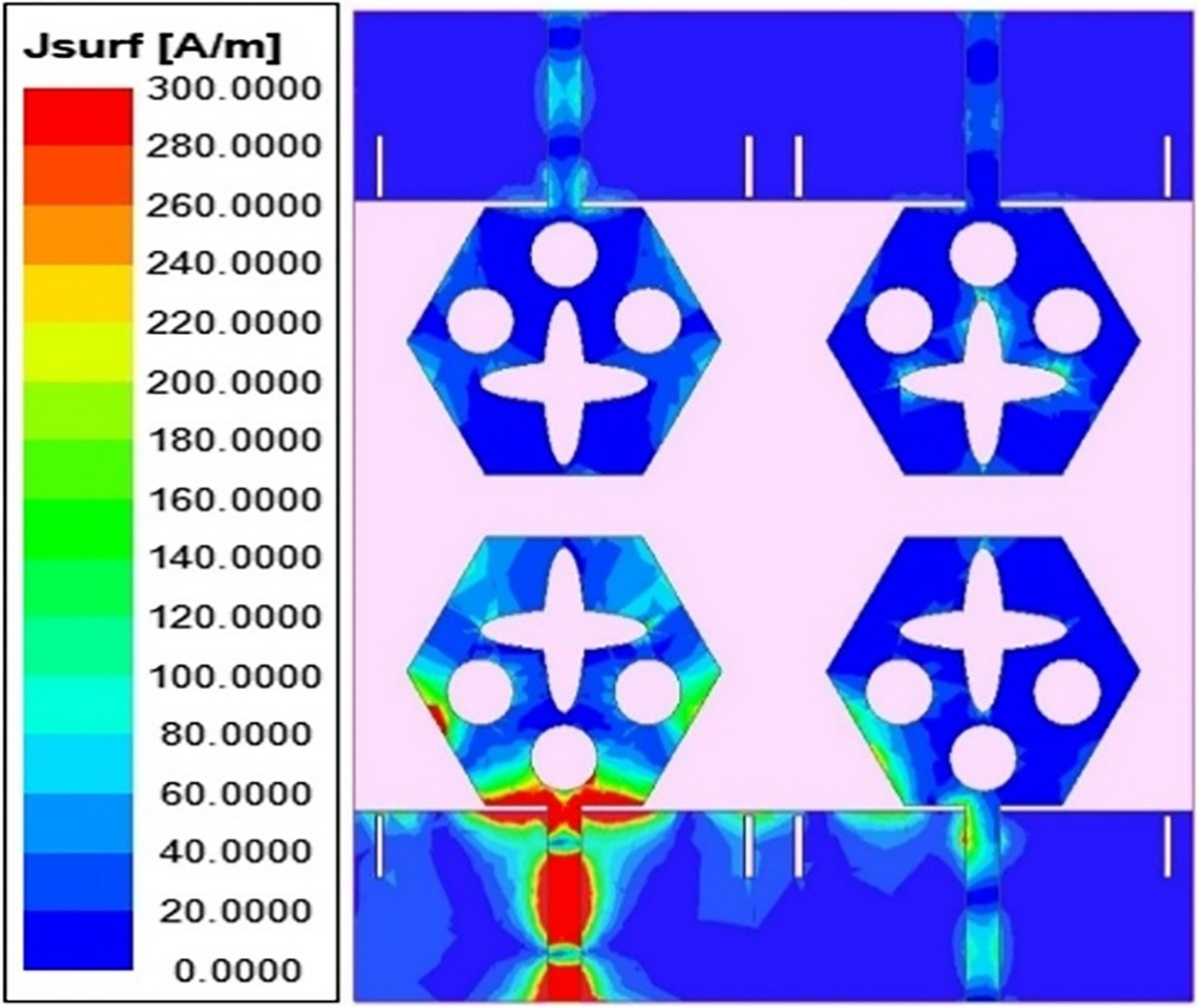
Surface-current-density_DWMB_ distribution without de-coupling structure.

**Fig 51:**
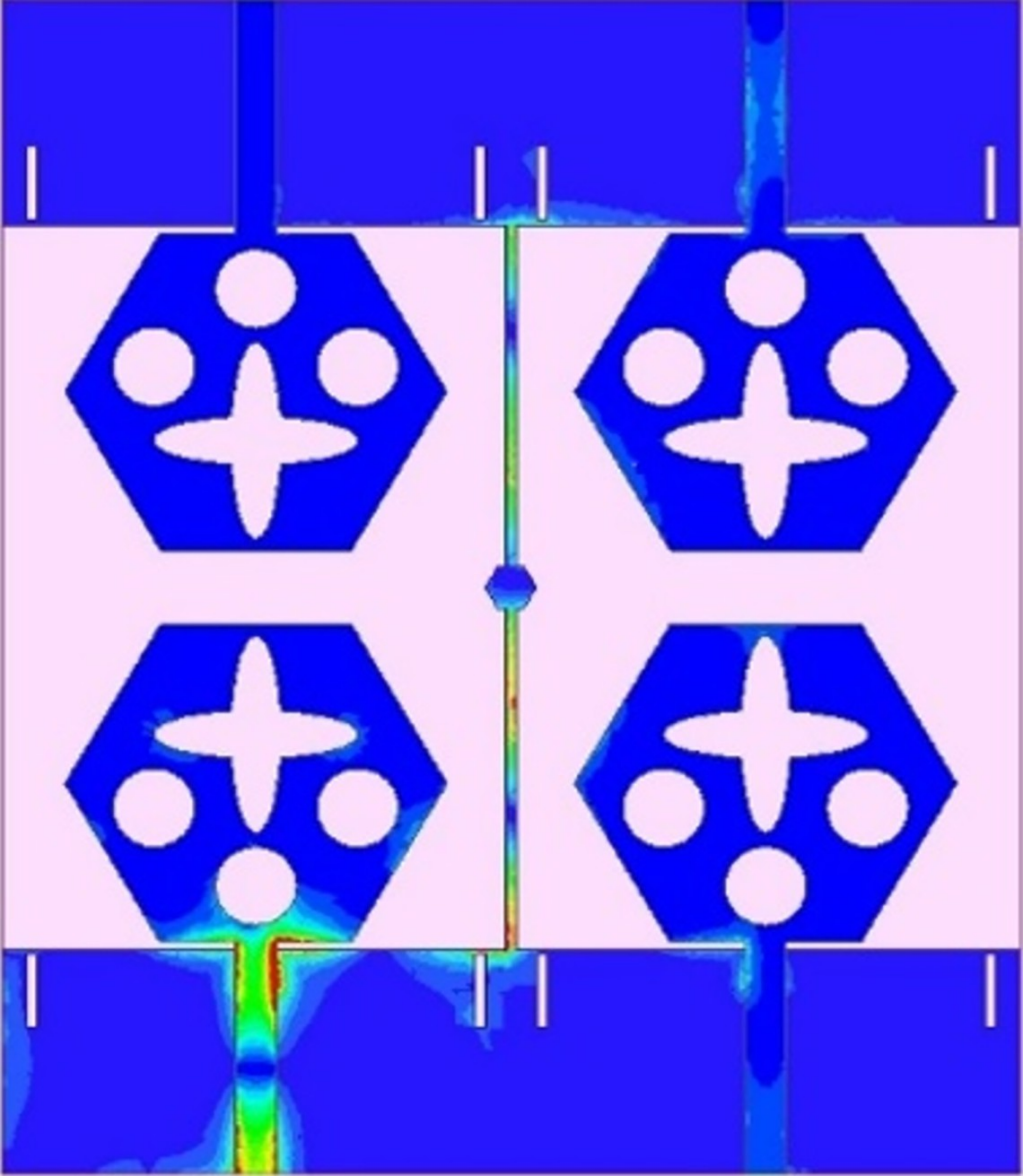
Surface-current-density_DWMB_ distribution with de-coupling structure.

The transmission coefficients for Band 1 in Fig 47 are below -10.0dB and for Band 2, these values are below -15.0 dB. For measured S-parameters, transmission coefficients are less than -20.0dB and -25.0dB in Band 1 and Band 2 noted in Fig 49. To better understand the concept of de-coupling, the surface-current-density distribution is studied as shown in Figs 50 and 51. The signal input to Antenna A (Fig 43) does interfere with neighboring radiating antennas having more impact on Antenna B which is placed adjacent to it. It can be also observed that Antenna C and Antenna D are also affected by radiations of Antenna A and this is due to mutual coupling occurring between them. Hence, the de-coupling structure attached to both the common ground for the pair of adjacent placed antennae shown in Fig 51 provides the solution to reduce the coupling and improve the isolation. This modification also eliminates the problem of un-connected ground and the proposed MIMO antenna can be easily integrated with MIC circuits. The de-coupling structure provides the path for the current flowing and hence prevents it from entering the adjacent radiating element & thereby reducing the effects of mutual coupling. The four-port MIMO antenna utilizes a spatial-diversity scheme and hence, diversity performance needs to be evaluated including ECC, DG, TARC, and CCL. The correlation coefficient (CC) is the degree of isolation between the communication channels. This is evaluated by using different methods such as radiation pattern characteristics formed by the radiating elements when the MIMO configuration is operating in the communication environment. The extracted S-parameters and the 3-D radiation patterns are utilized to calculate the Envelope-CC and the following formulas are used to evaluate [37]

Where $\gamma_{d}^{2}\gamma_{s}^{2}$ are the variance(s) of corresponding ports and are expressed as [37]

$$\rho_{e}={\left|\rho_{c}\right|}^{2}$$ (14)

$$ECC=\rho_{e}(m,s,N)=\frac{{\left|\sum_{n=1}^{N}S_{m,n}^{*}S_{n,s}\right|}^{2}}{\pi_{k=(m,s)}[1-\sum_{n=1}^{N}S_{m,n}^{*}S_{n,k}]}$$ (15)

Eq 12 shows the calculation of ECC_DWMB_ between the m^th^ and the s^th^ port and in general is calculated by following equation [37]

$$ECC=\frac{{\left|S_{mm}^{*}S_{ms}+S_{sm}^{*}S_{ss}\right|}^{2}}{\left(1-{\left|S_{ii}\right|}^{2}-{\left|S_{sm}\right|}^{2})(1-{\left|S_{ss}\right|}^{2}-{\left|S_{ms}\right|}^{2}\right)}$$ (16)

For two-port and four-port_DWMB_, ECC is given by

$${ECC}_{\left(Two\;Port\right)}=\frac{{\left|S_{11}^{*}S_{12}+S_{12}^{*}S_{22}\right|}^{2}}{\left(1-{\left|S_{11}\right|}^{2}-{\left|S_{21}\right|}^{2})(1-{\left|S_{12}\right|}^{2}-{\left|S_{22}\right|}^{2}\right)}$$ (17)

$${ECC}_{\left(Four\;Port\right)}=\frac{{\left|S_{11}^{*}S_{12}+S_{12}^{*}S_{22}{+S}_{13}^{*}S_{32}+S_{14}^{*}S_{42}\right|}^{2}}{\left(1-{\left|S_{11}\right|}^{2}-{\left|S_{21}\right|}^{2}-{\left|S_{31}\right|}^{2}-{\left|S_{41}\right|}^{2})(1-{\left|S_{12}\right|}^{2}-{\left|S_{22}\right|}^{2}-{\left|S_{32}\right|}^{2}-{\left|S_{42}\right|}^{2}\right)}$$ (18)

**Fig 52:**
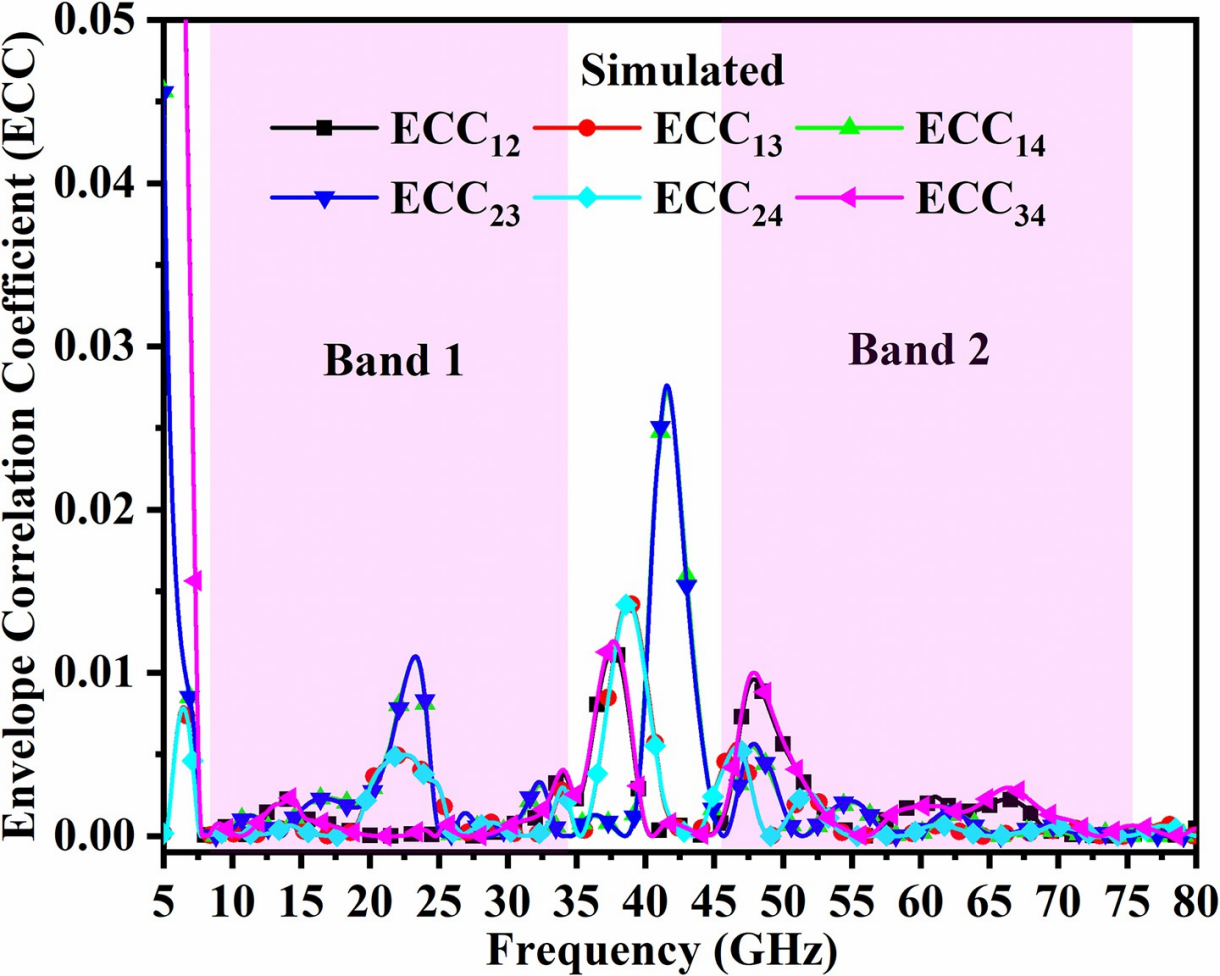
Simulated ECC_DWMB_.

**Fig 53:**
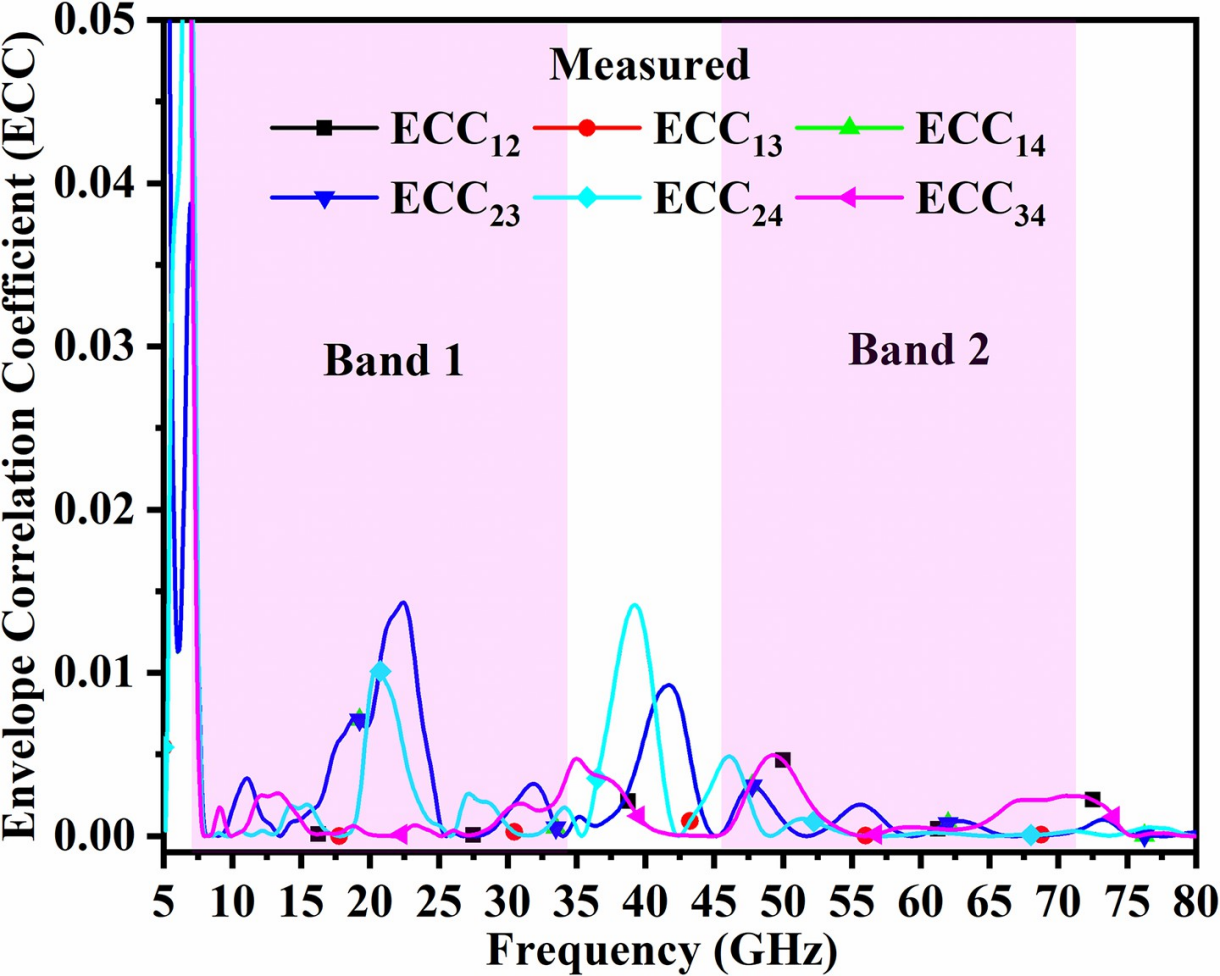
Measured ECC_DWMB_.

The variation of ECC lies between 0 to 1 indicating null interference and is the ideal case with perfect matching of the impedance and each radiation is well isolated from each other produced by each MIMO_DWMB_ radiating element. However, practically, this condition is difficult to achieve, and standard values below 0.50 need to be achieved on calculations from the above set of Equations. The simulated and measured ECC are calculated between Port 1-Port 2 (ECC_12_), Port 1-Port 3 (ECC_13_), Port 1-Port 4 (ECC_14_), Port 2-Port 3 (ECC_23_), Port 2- Port 4 (ECC_24_) and Port 3-Port 4 (ECC_34_). In the proposed work, the simulated and measured ECC in both the bands (Band 1 and Band 2) are 0.02 as noted from Figs 52 and 53 which indicates that the individual radiating patterns of each antenna have very little interaction with each other.

The Diversity-Gain_DWMB_ (DG) is calculated to quantify the performance characteristics of the diversity scheme used (spatial, polarization, or radiation diversity). The DG_DWMB_ is related to ECC_DWMB_ by the following formula [37]

$$DG=10\sqrt{1-{\left|\rho_{e(DWMB)}\right|}^{2}}$$ (19)

**Fig 54:**
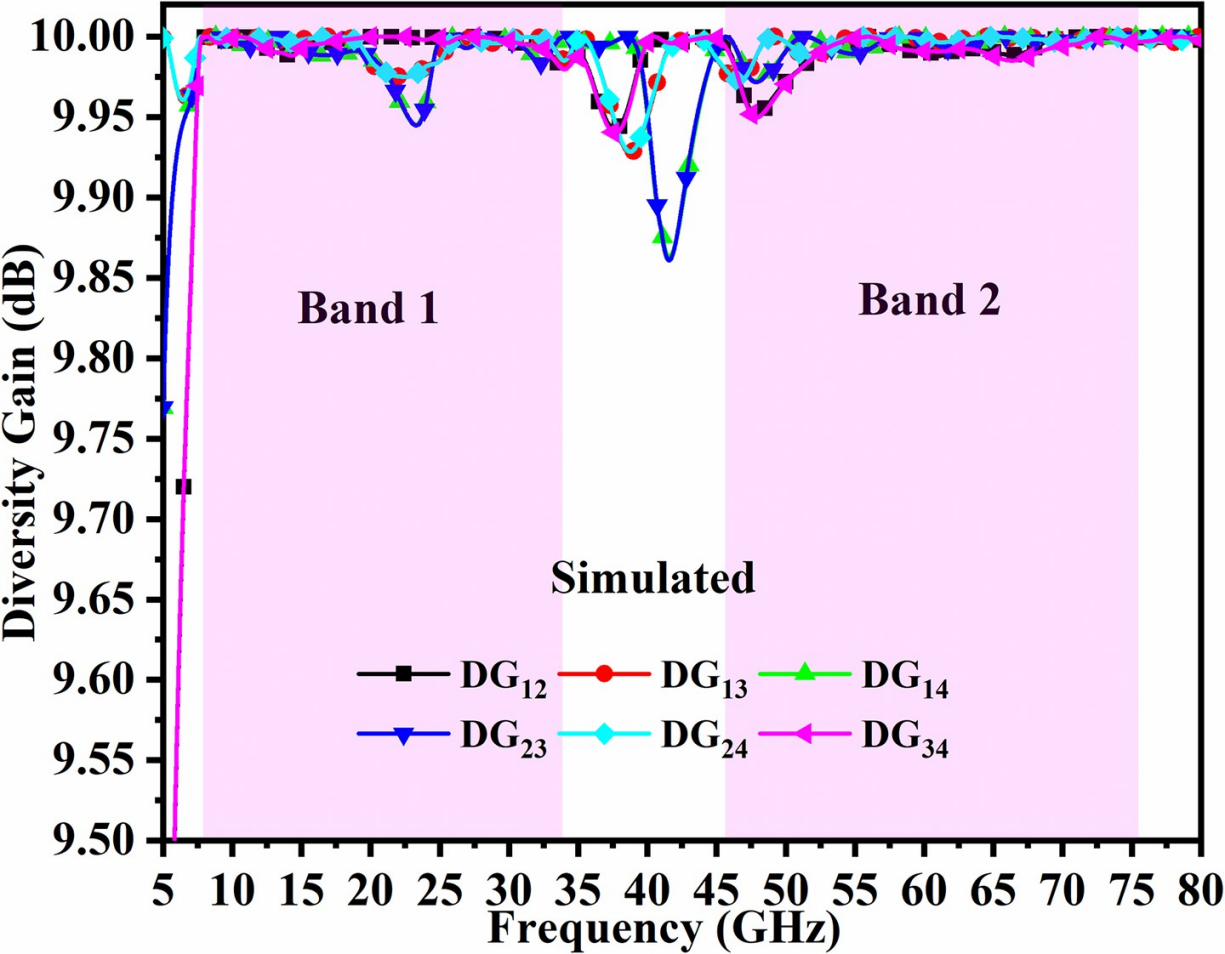
Simulated DG_DWMB_.

**Fig 55:**
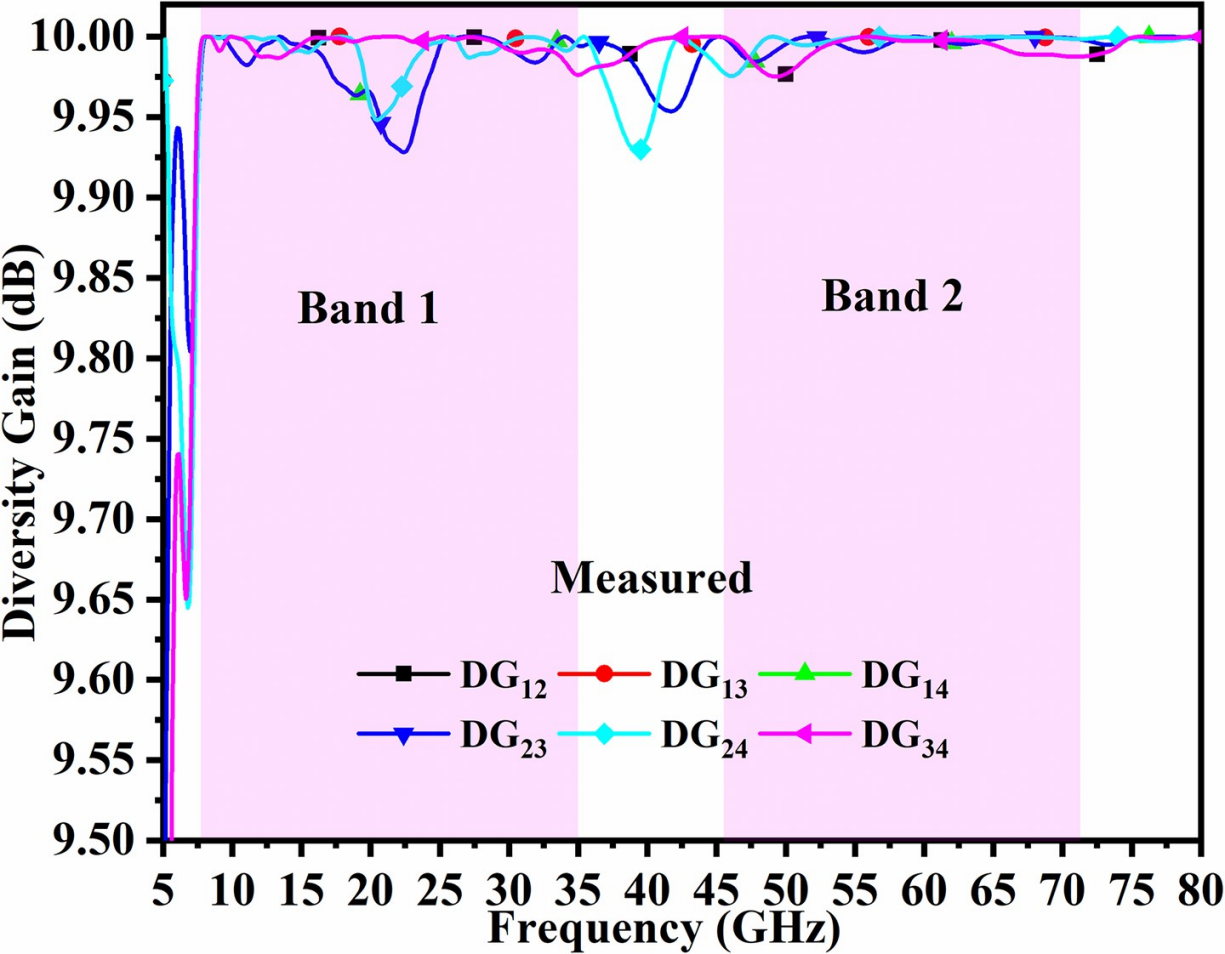
Measured DG_DWMB_.

The standardized values for Div. Gain is >9.950dB. For the proposed four-port MIMO_DWMB_ antenna configuration, the Band 1 values are more than 9.950dB and Band 2 corresponds to more than 9.999 dB. These values for wider impedance bandwidth are plotted in Figs 54 and 55.

The perfect matching of impedance is achieved in the proposed work for wider bandwidth in two operating bands (Band 1 & Band 2). This also results in highly matched S-parameters (reflection & transmission coefficients) but, only this parameter cannot alone judge the performance related to the diversity and hence, a new metric is introduced known as Total-Active-reflection-Coefficient_DWMB_ (TARC) which is evaluated by Equations given below

$$\Gamma_{a}^{t}=\frac{Available\;Power(AP)-Radiated\;Power(RP)}{Available\;Power(AP)}$$ (20)

**Fig 56:**
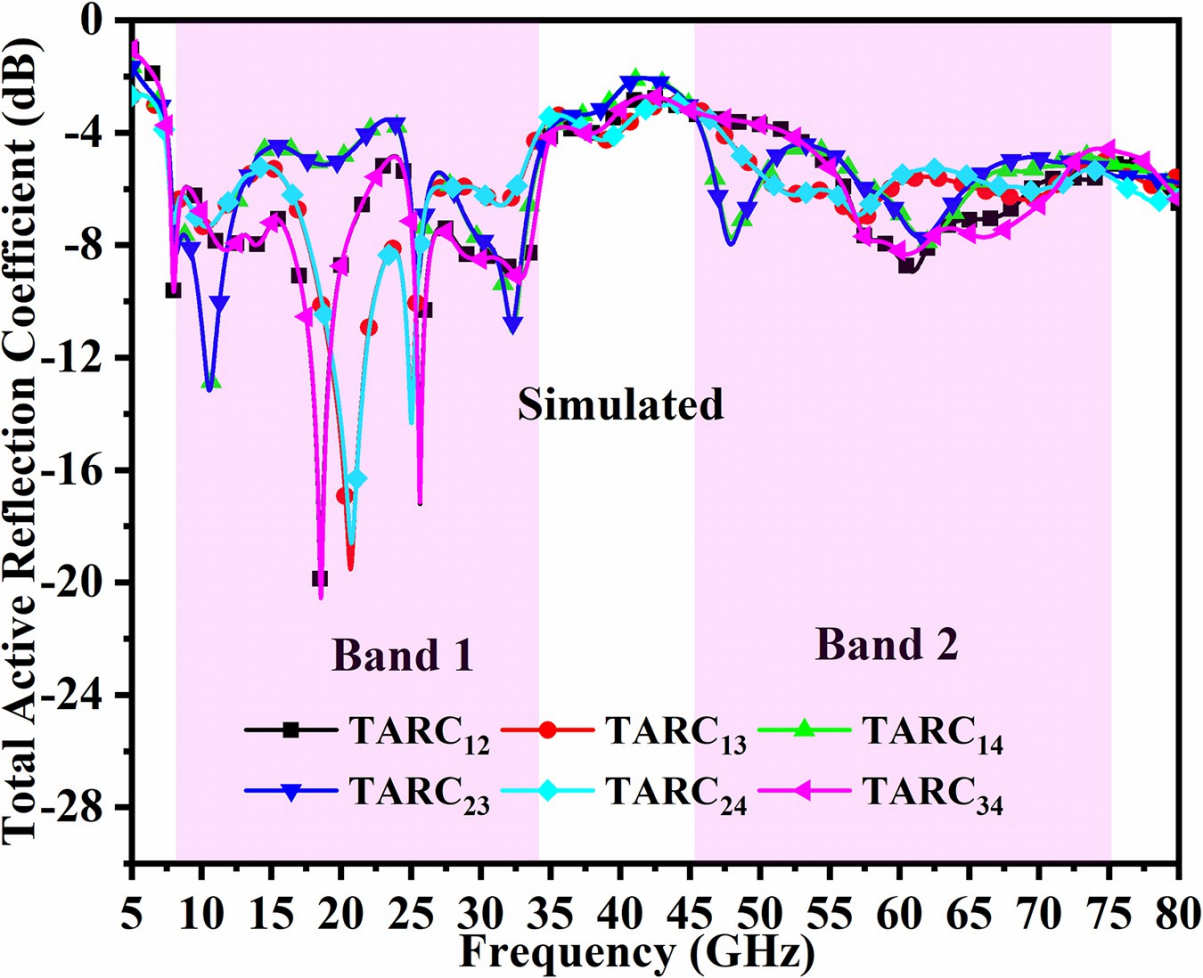
Simulated TARC_DWMB_.

**Fig 57:**
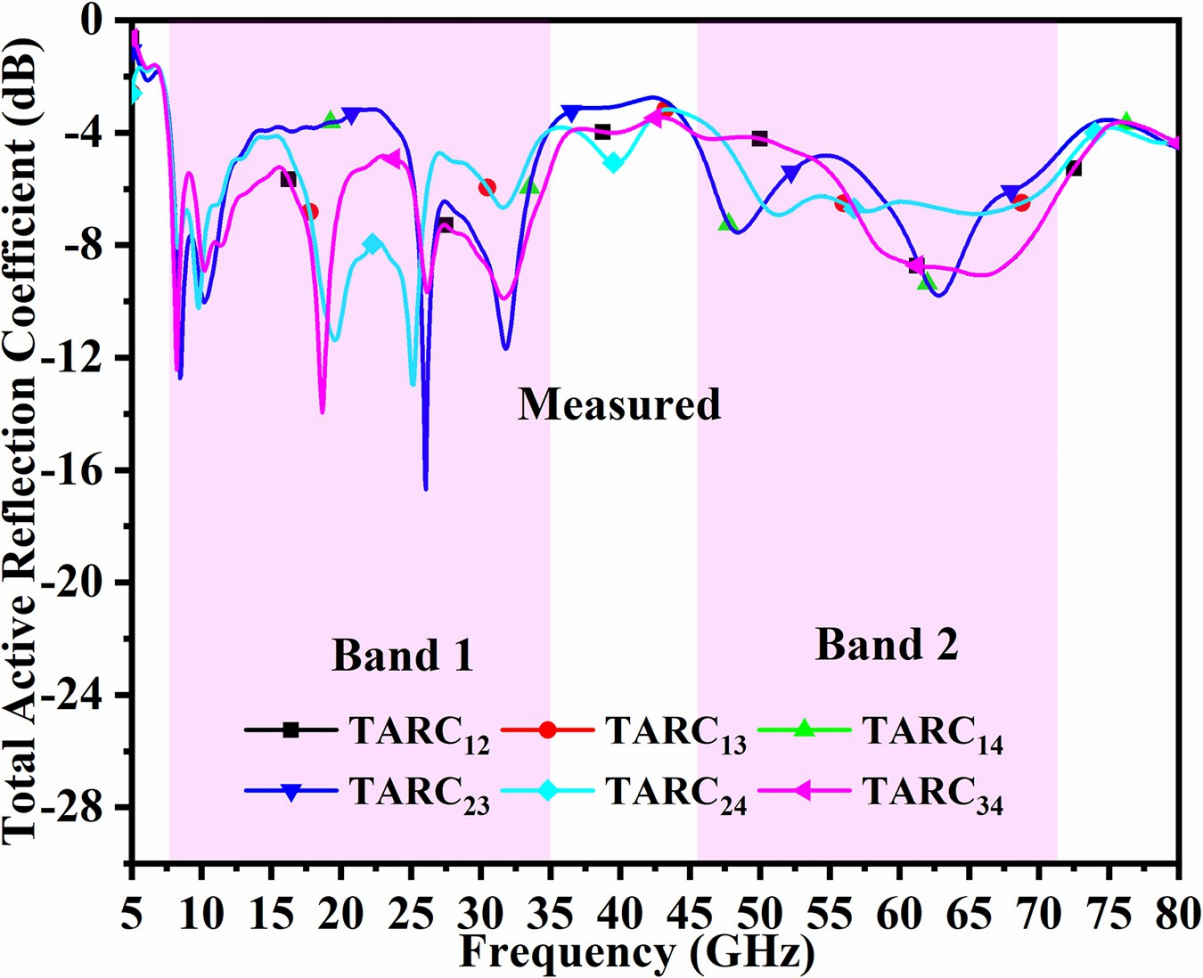
Measured TARC_DWMB_.

In general, for the ideal-case (loss-less) MIMO_DWMB_ system, the values for TARC_DWMB_ = 0.0dB are calculated for any combination of two ports. Figs 56 and 57 represents the simulated-measured TARC values with minimum value more than -4.0dB in Band1 and Band2 respectively.

$$TARC=\frac{\sqrt{\sum_{i=1}^{N}{\left|m_{i}\right|}^{2}}}{\sqrt{\sum_{i=1}^{N}{\left|s_{i}\right|}^{2}}}$$ (21)

[m] and [n] correspond to incident and reflected power in dB. The TARC_DWMB_ concerning phase values of S-parameters are given below

$$b_{1}=S_{11}a_{1}+S_{12}a_{2}=S_{11}a_{0}e^{j\theta_{1}}+S_{12}a_{0}e^{j\theta_{2}}=a_{1}(S_{11}+S_{12}e^{j\theta })$$ (22)

$$b_{2}=S_{21}a_{1}+S_{22}a_{2}=S_{21}a_{0}e^{j\theta_{1}}+S_{22}a_{0}e^{j\theta_{2}}=a_{1}(S_{21}+S_{22}e^{j\theta })$$ (23)

Combining Equations, TARC_DWMB_

$$TARC=\frac{\sqrt{{\left|S_{11}+S_{12}e^{j\theta }\right|}^{2}+{\left|S_{21}+S_{22}e^{j\theta }\right|}^{2}}}{\sqrt{2}}$$ (24)

Assuming phase for both incident and reflected wave to be θ = 0°, the TARC_DWMB_ are calculated for Port1-Port2, Port1-Port3, Port1-Port4, Port2-Port3, Port2-Port4, and Port3-Port4 which corresponds to -4.0dB (Band 1 & Band 2) for simulation environment and -6.0dB (Band 1 & Band 2) for measured values noted from Figs 56 and 57.

The signal transmitted between Tx to Rx does suffer some loss in the MIMO_DWMB_ system when the proposed antenna is deployed in the wireless communication environment and this is signified by the loss of bits in the channel which should be less than 0.40bits/second/Hz [37]. This is evaluated by

$$CCL=-{log}_{2}det(\alpha^{s})$$ (25)

where

$$\rho_{mm}=1-\sum_{n=1}^{4}{\left|S_{mn}\right|}^{2}$$ (26)

$$\rho_{ms}=-(S_{mm}^{*}S_{ms}+S_{sm}^{*}S_{ms})$$ (27)

**Fig 58:**
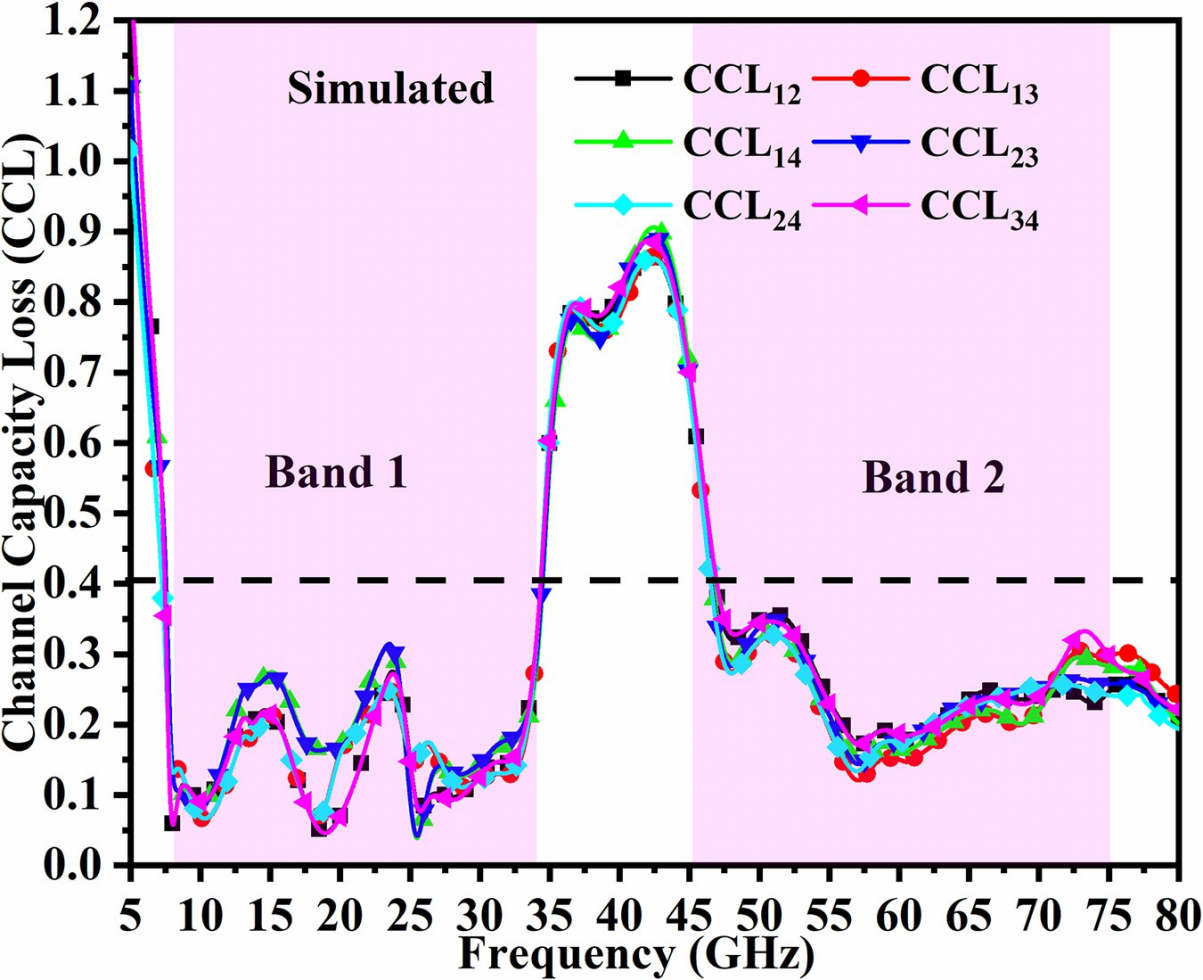
Simulated CCL_DWMB_.

**Fig 59:**
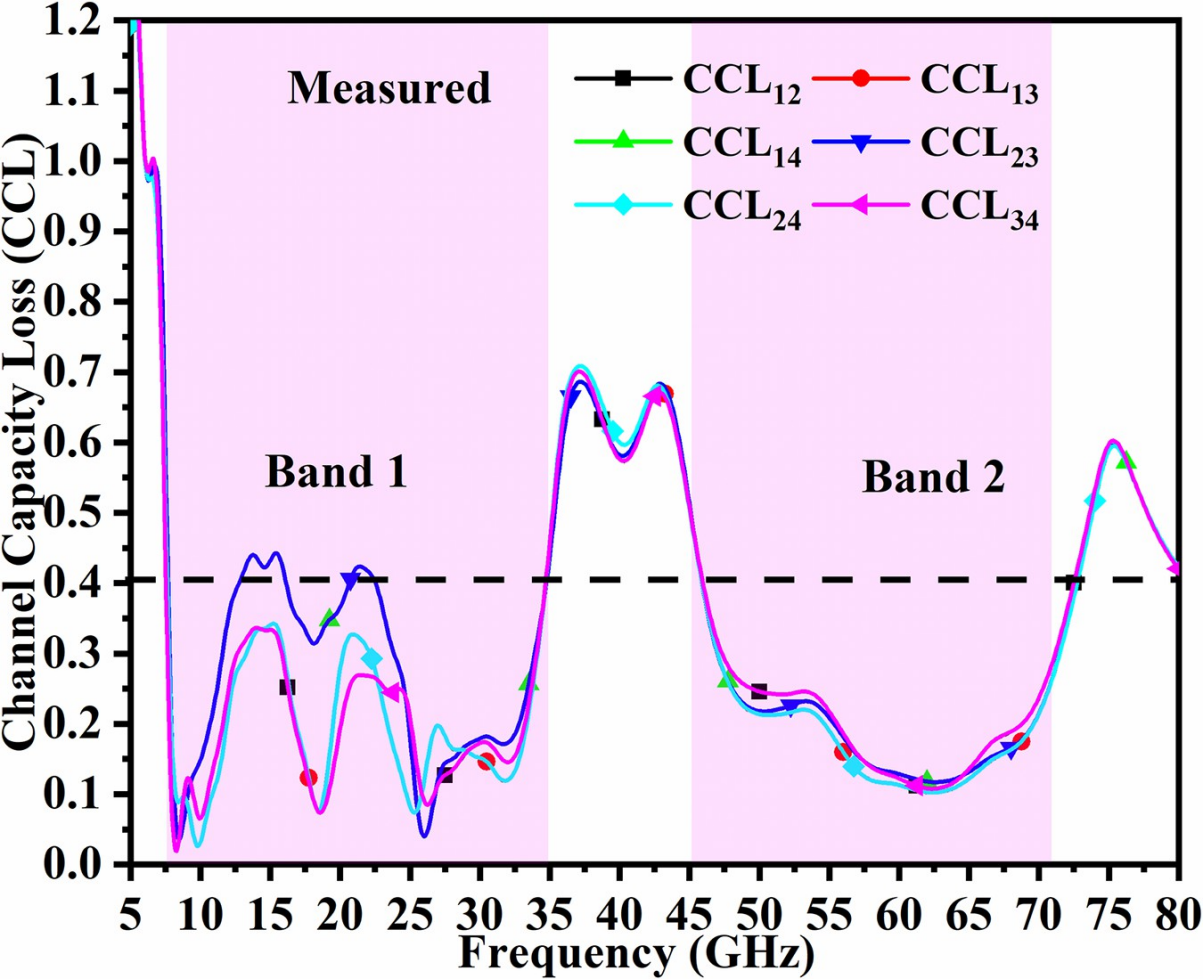
Measured CCL_DWMB_.

In the proposed work, the average values are CCL≤0.1b/s/Hz&CCL≤0.1b/s/Hz for simulation and measured results as depicted in Figs 58 and 59.

**Fig 60:**
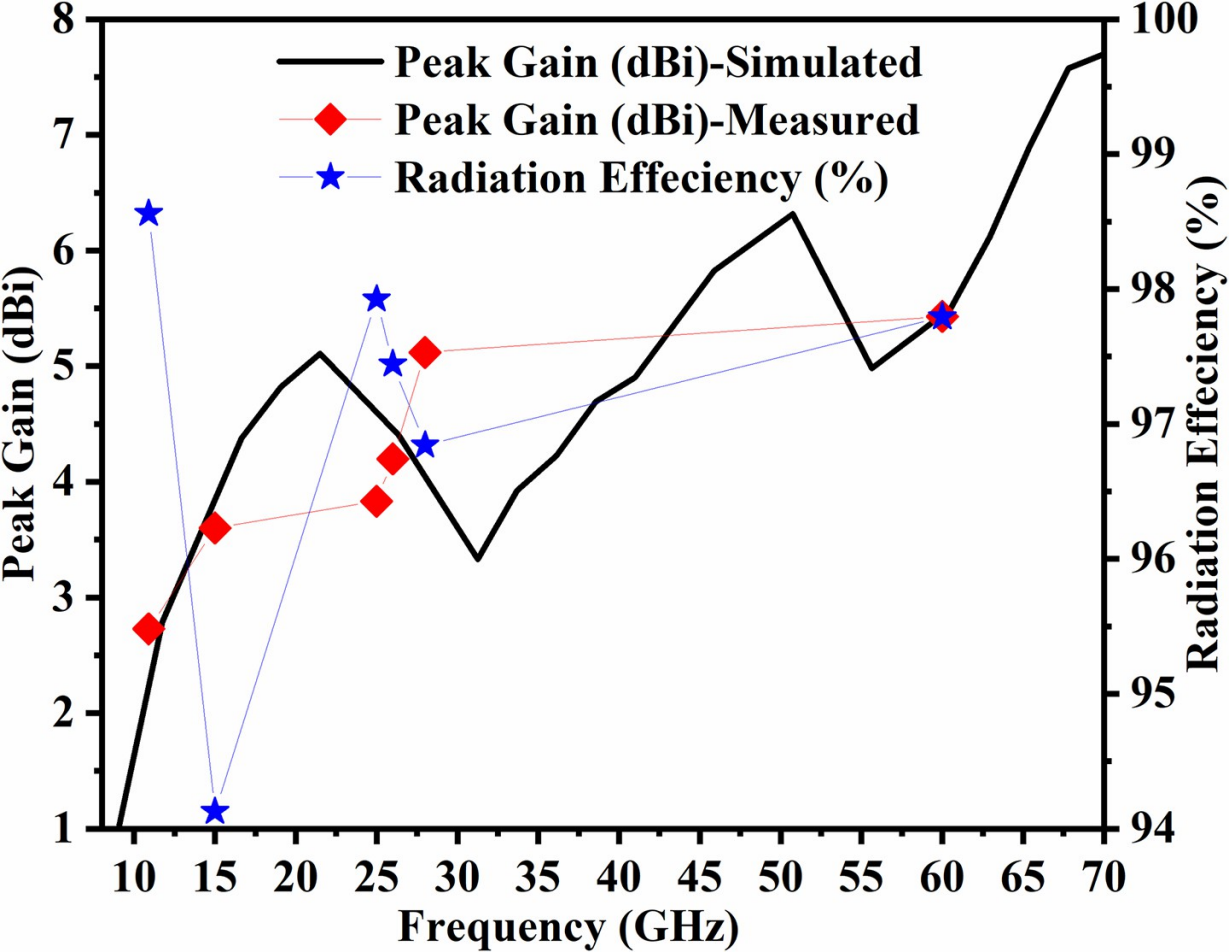
Simulated-measured peak gain & radiation efficiency.

**Fig 61:**
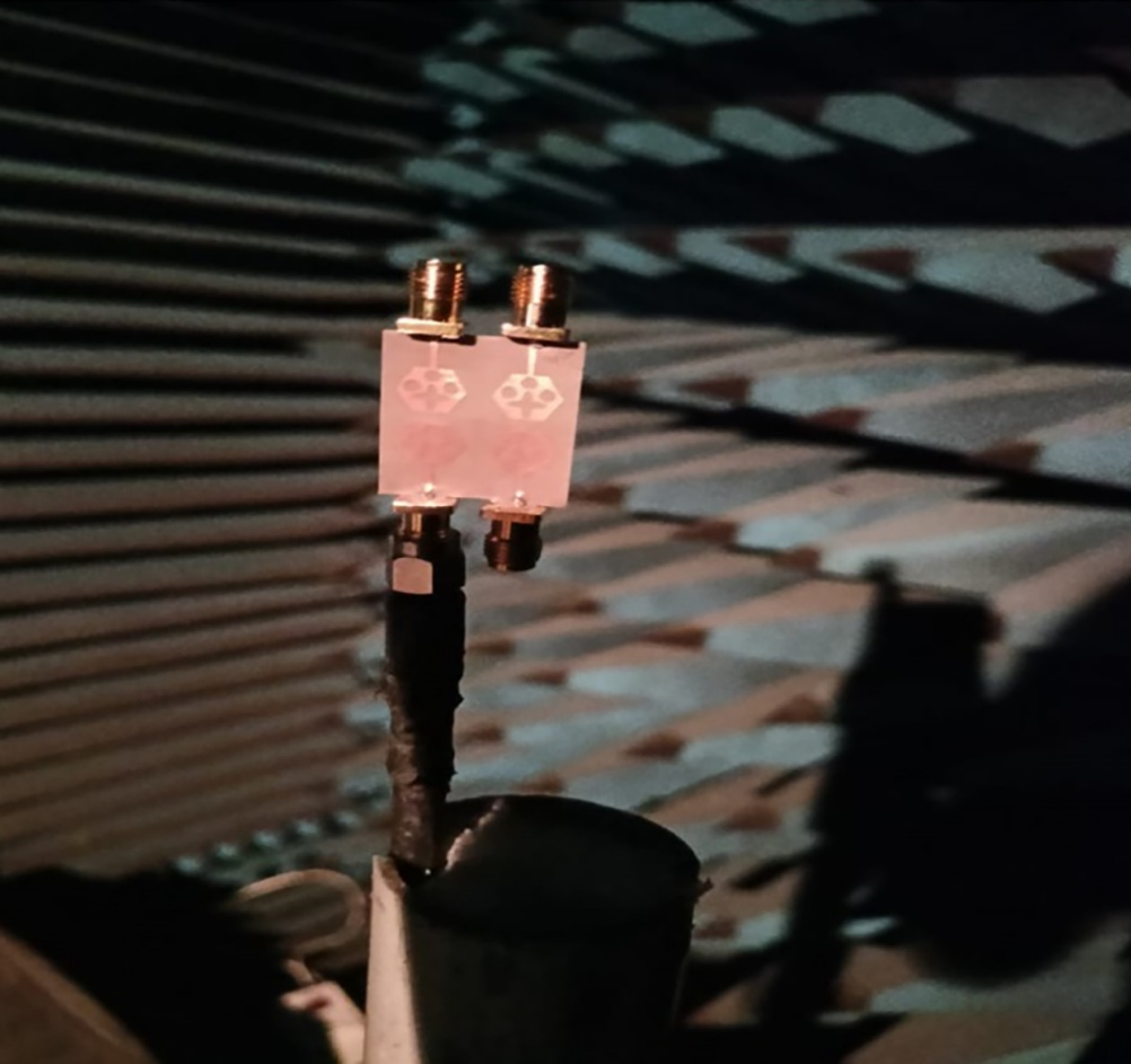
Photograph of the antenna within the anechoic-chamber.

**Fig 62:**
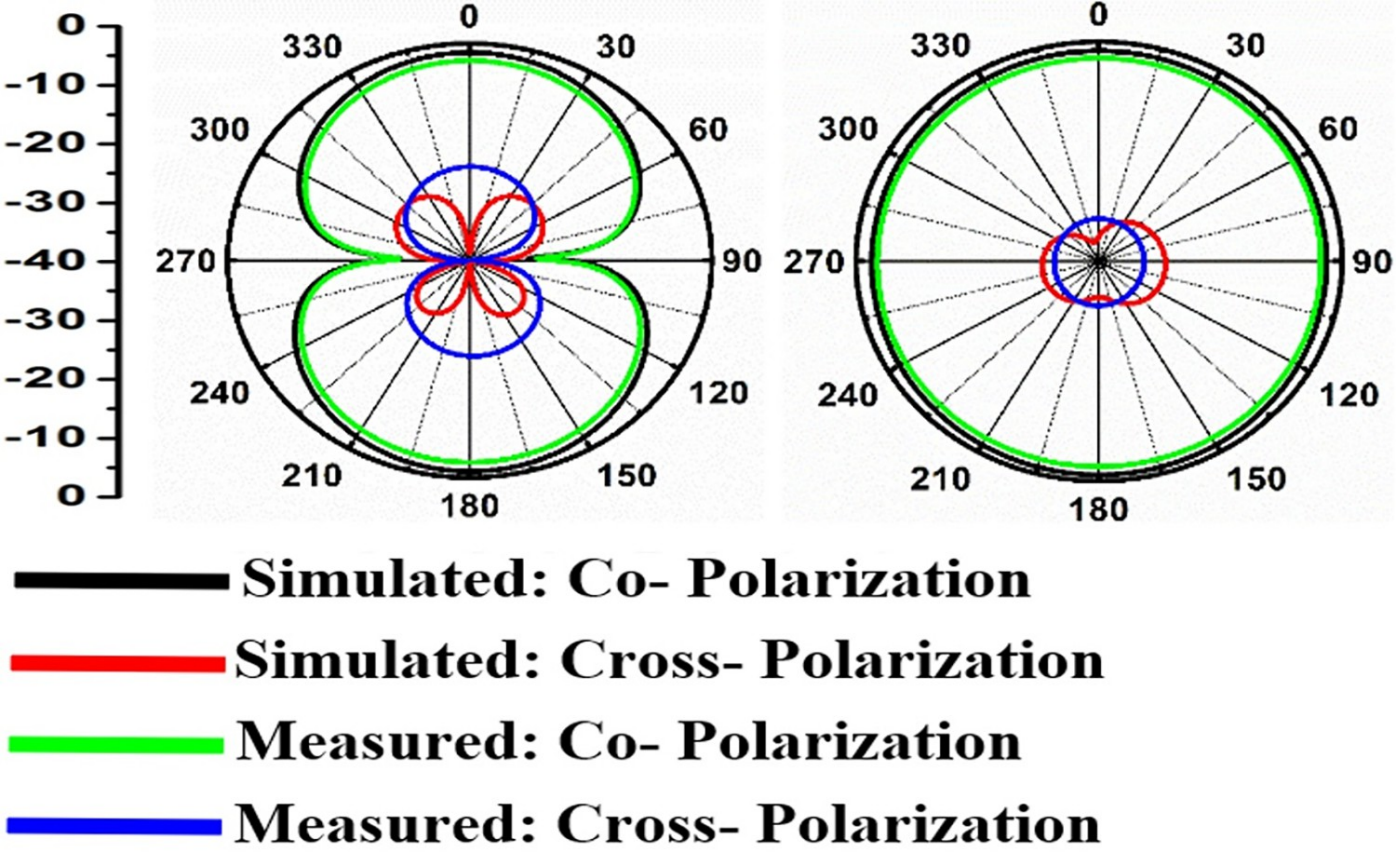
Simulated-measured 2-D radiation pattern at 10.0GHz.

**Fig 63:**
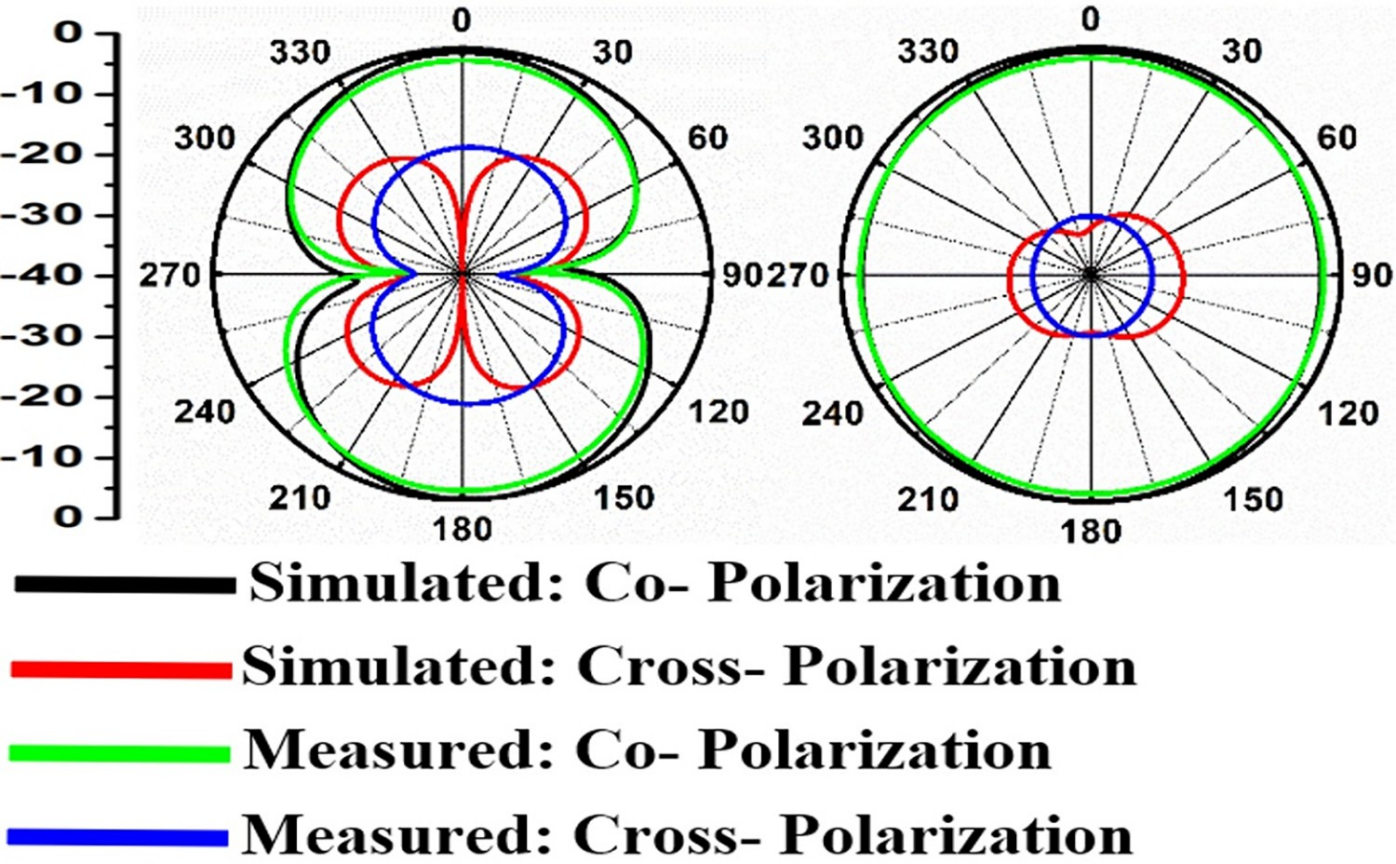
Simulated-measured 2-D radiation pattern at 15.0GHz.

**Fig 64:**
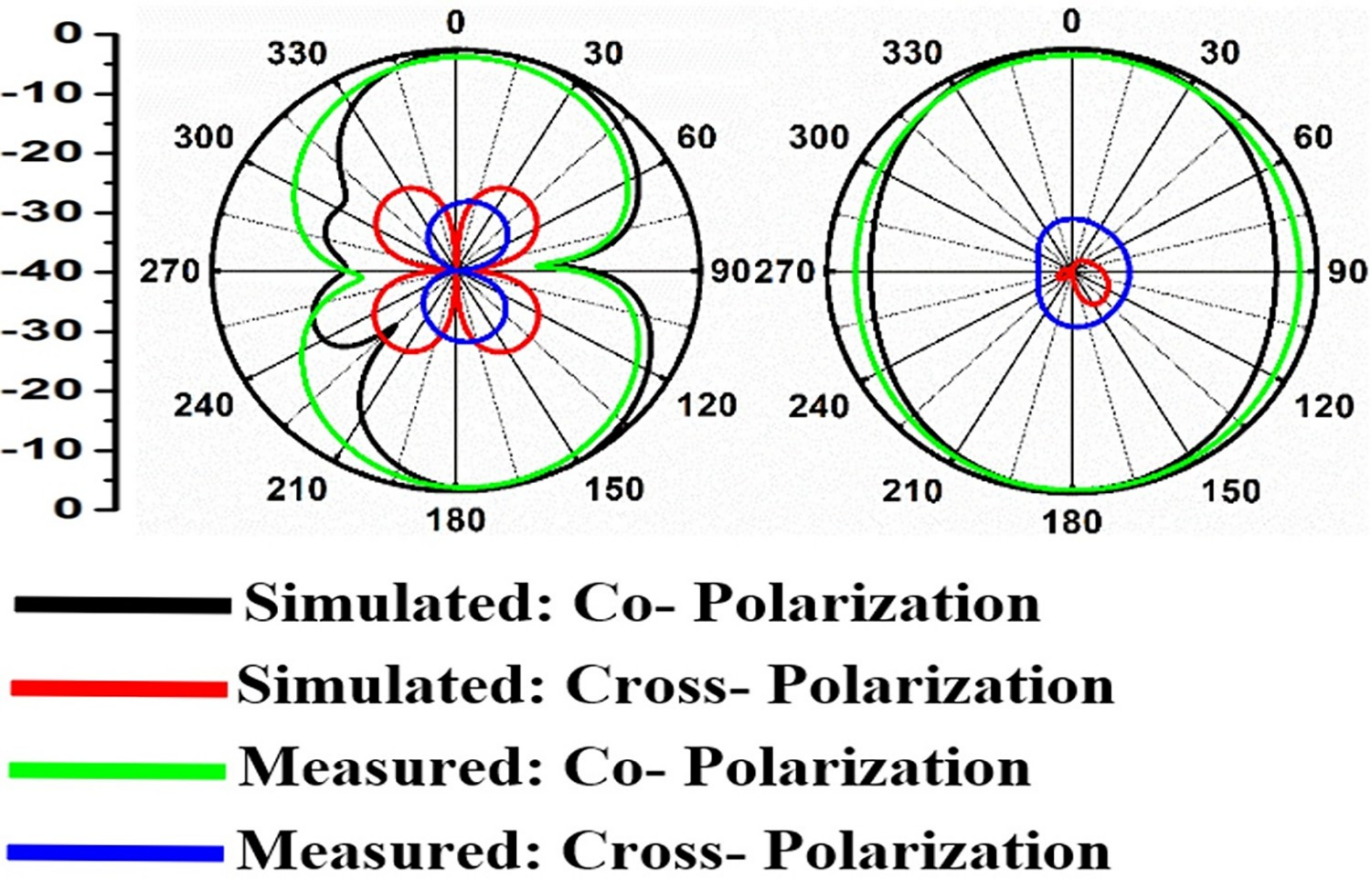
Simulated-measured 2-D radiation pattern at 24.0GHz.

**Fig 65:**
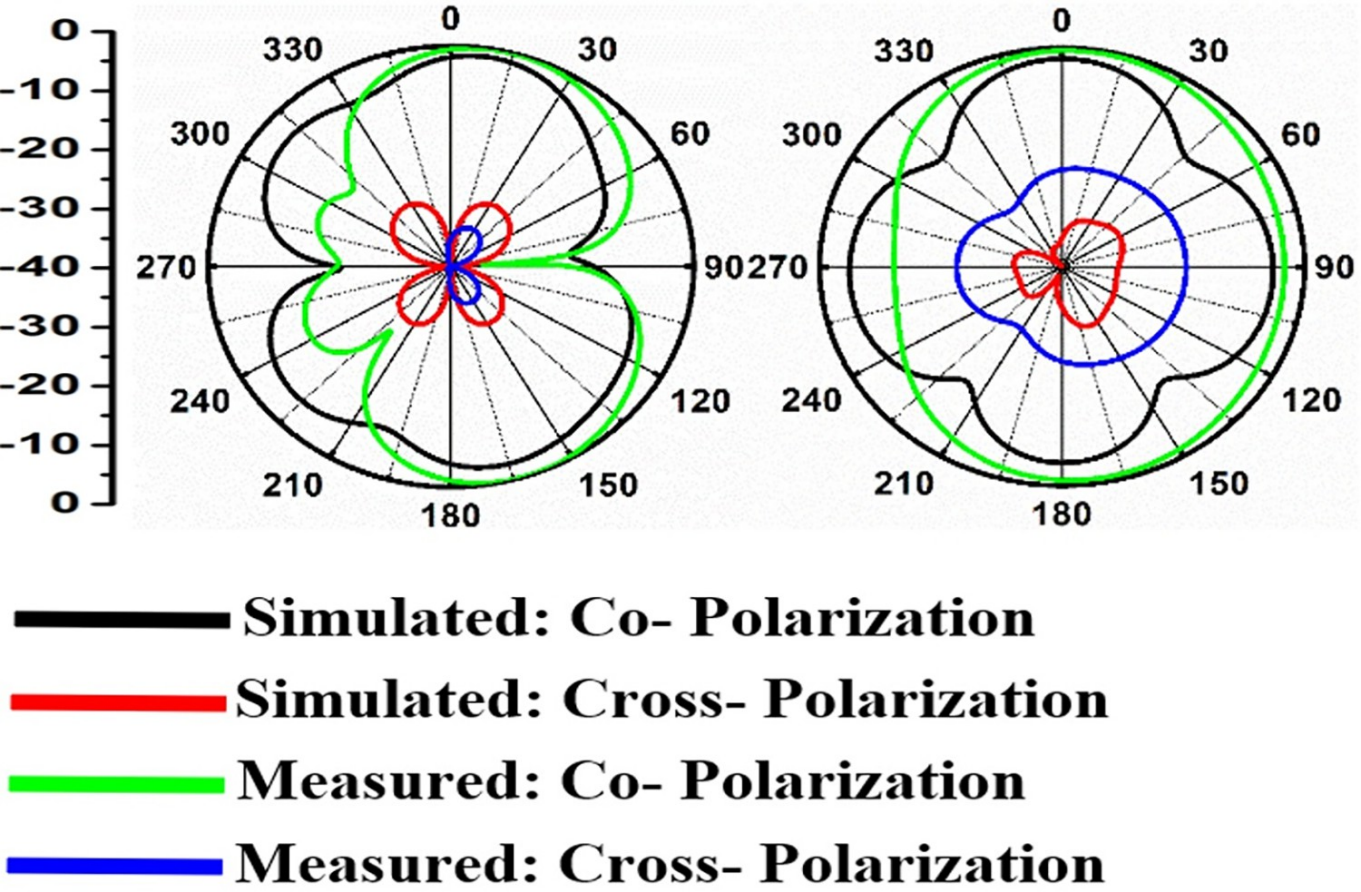
Simulated-measured 2-D radiation pattern at 26.0GHz.

**Fig 66:**
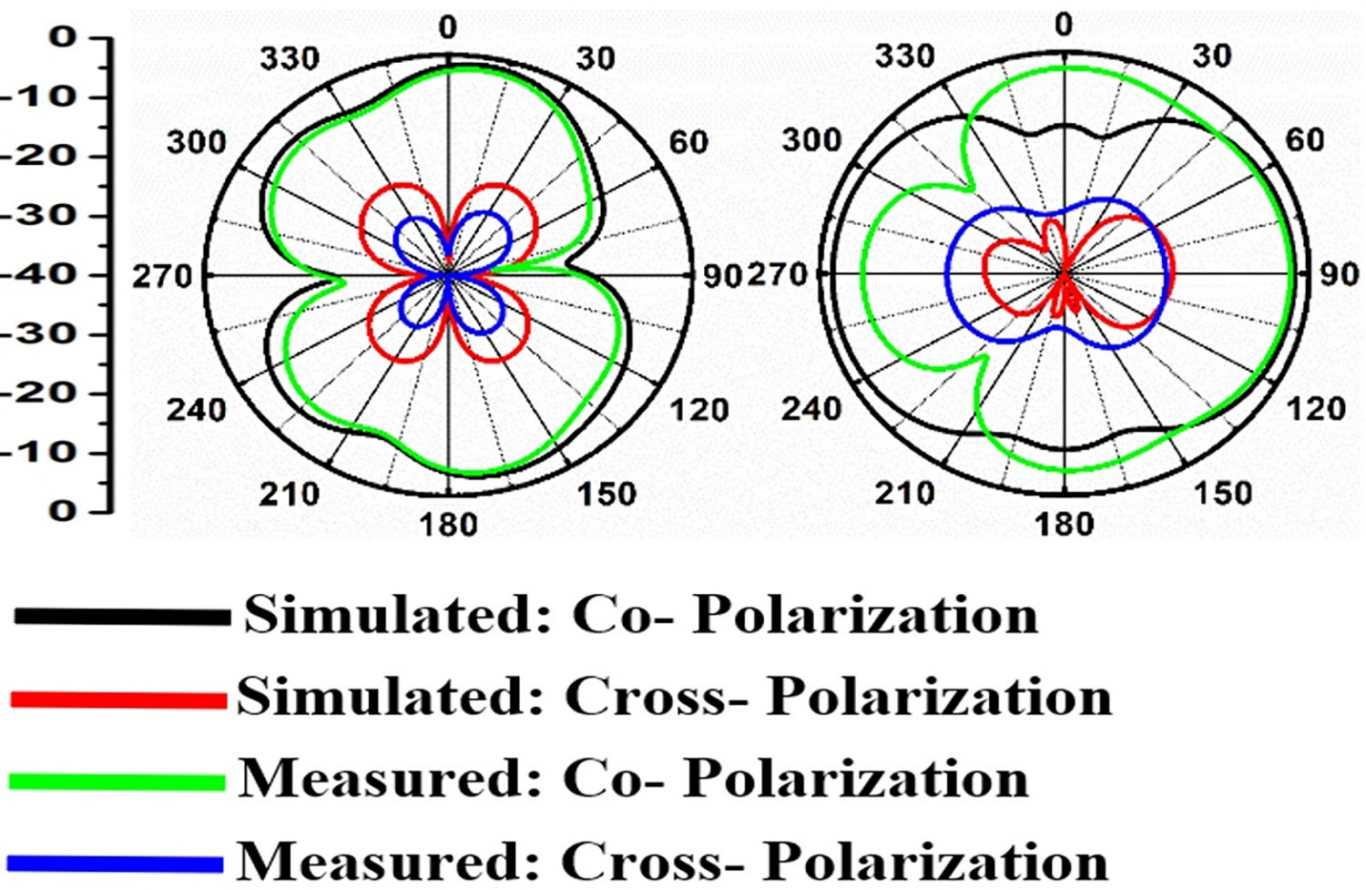
Simulated-measured 2-D radiation pattern at 28.0GHz.

**Fig 67:**
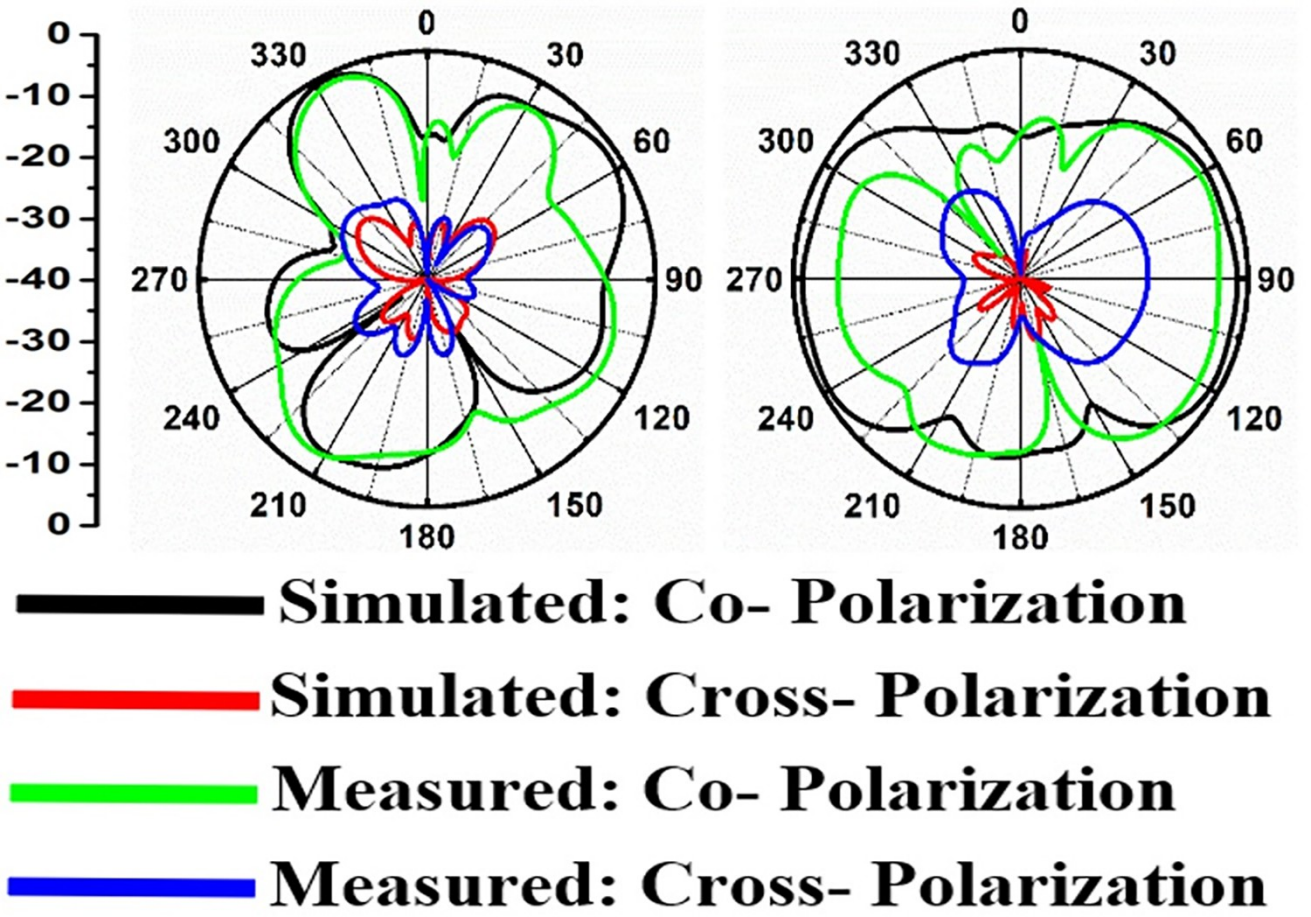
Simulated-measured 2-D radiation pattern at 60.0GHz.

Figs 60–67 records the simulated and measured results achieved for the Far-Field region with a comparison of peak-realized gain and 2-D radiation patterns for key-wireless applications in Band 1 and Band 2. Fig 61 also shows the prototype of the proposed four-port MIMO antenna placed within the anechoic chamber for gain and radiation measurements. Fig 60 records the plot of peak gain concerning frequency and simulated gain varies between 2.79dBi-7.71dBi. The measured peak gain corresponds to 2.73dBi at 10.0GHz, 3.60dBi at 15.0GHz, 3.83dBi at 24.0GHz, 4.20dBi at 26.0GHz, 5.12dBi at 28.0GHz and 5.43dBi at 60.0GHz respectively. Fig 60 also illustrates radiation efficiency with maximum value corresponding to 98.55% at 10.90GHz. Fig 61 corresponds to the proposed MIMO_DWMB_ within the anechoic-chamber for far-field measurements including gain and 2-D radiation patterns. The other values correspond to 94.12% at 15.0GHz, 97.92% at 25.0GHz, 97.44% at 26.0GHz, 96.84% at 28.0GHz and 97.79% at 60.0GHz. Figs 62–67 shows the plot of 2-D radiation patterns in xz and yz-planes. For frequency points corresponding to 10.0GHz, 15.0GHz, 24.0GHz, 26.0GHz and 28.0GHz, the

**Fig 68:**
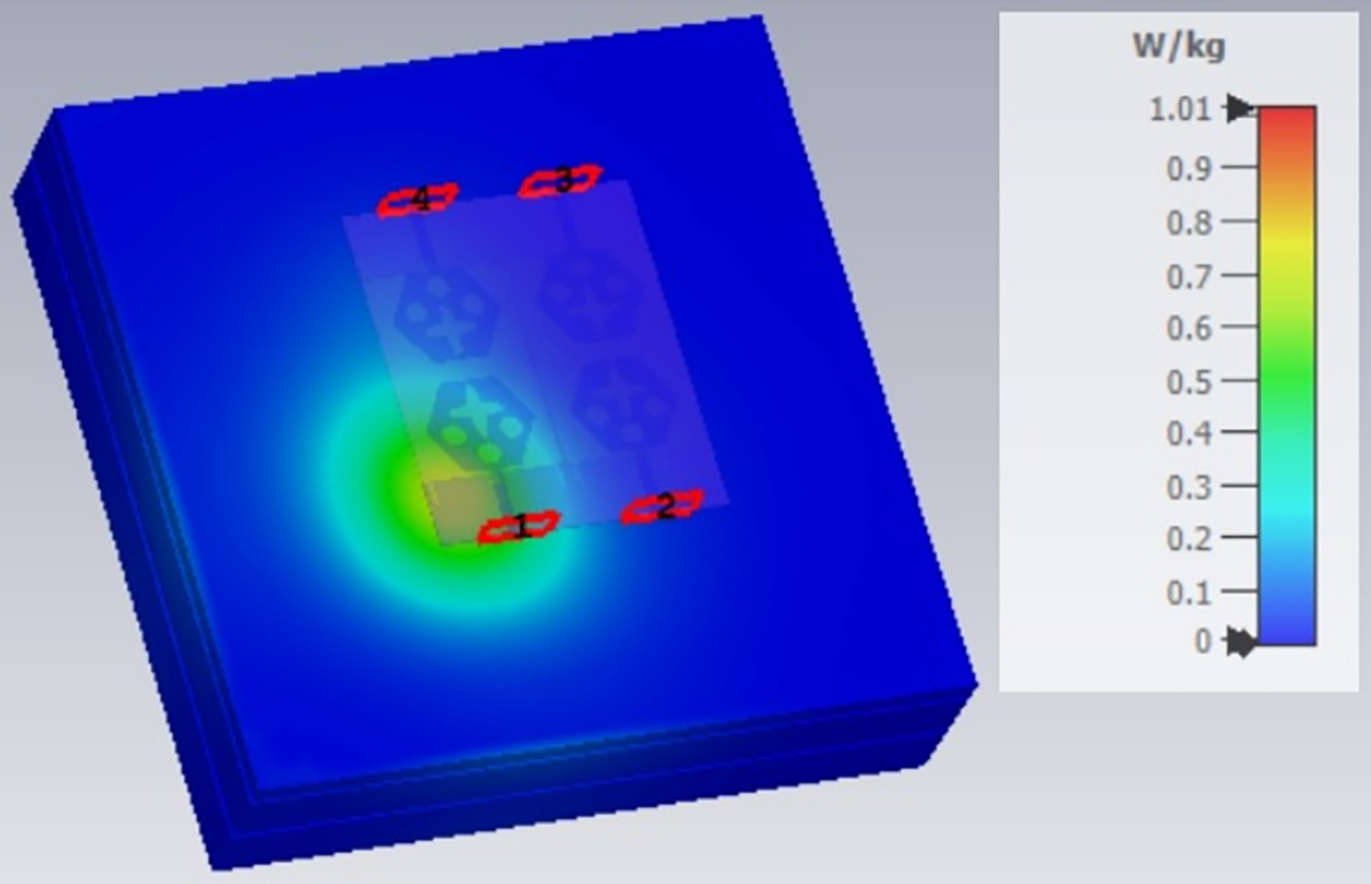
SAR analysis at 10.0GHz.

**Fig 69:**
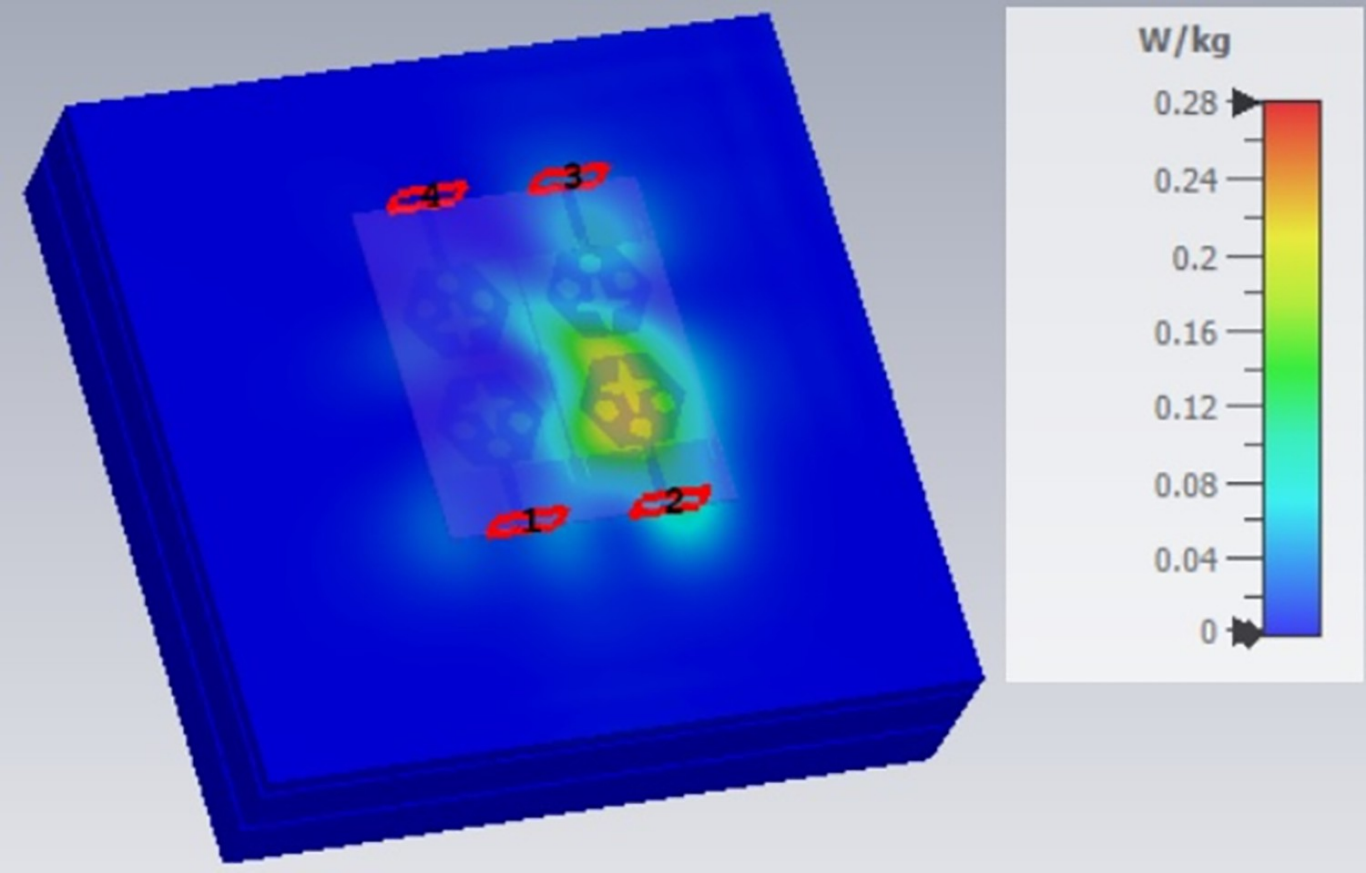
SAR analysis at 15.0GHz.

**Fig 70:**
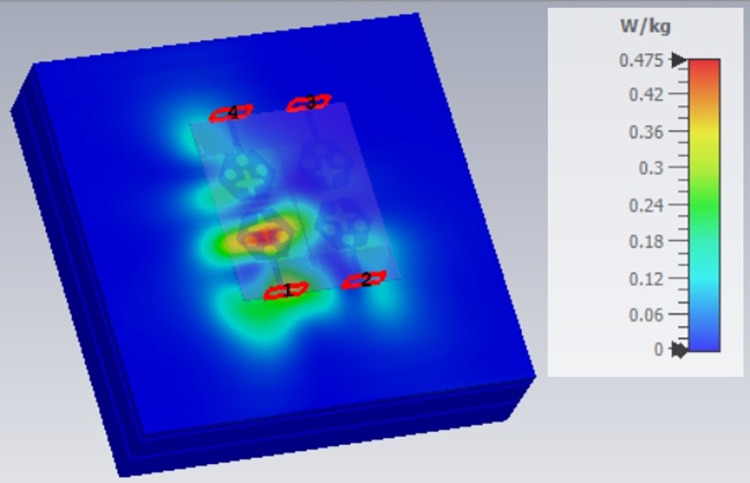
SAR analysis at 24.0GHz.

**Fig 71:**
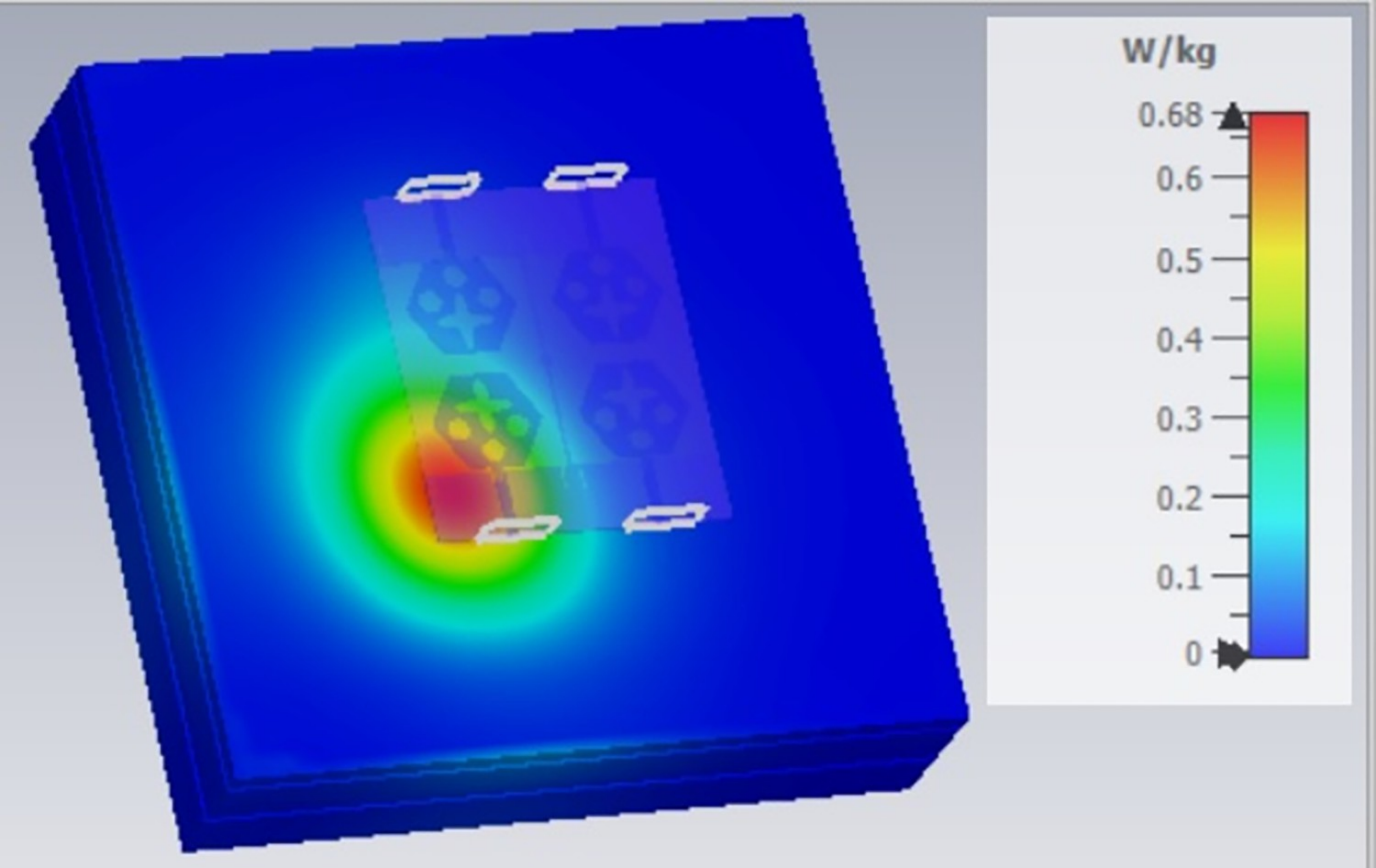
SAR analysis at 26.0GHz.

**Fig 72:**
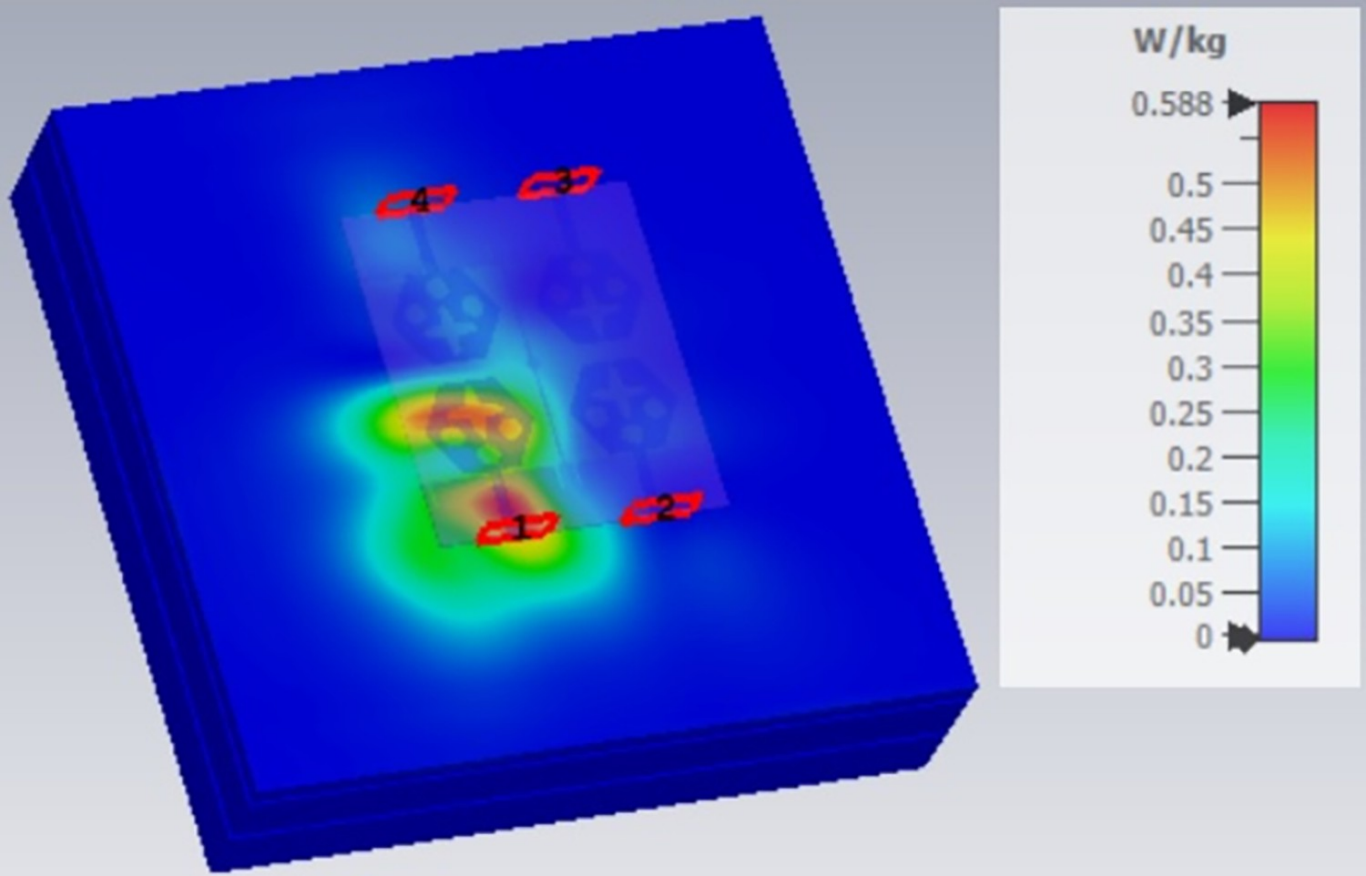
SAR analysis at 28.0GHz.

**Fig 73:**
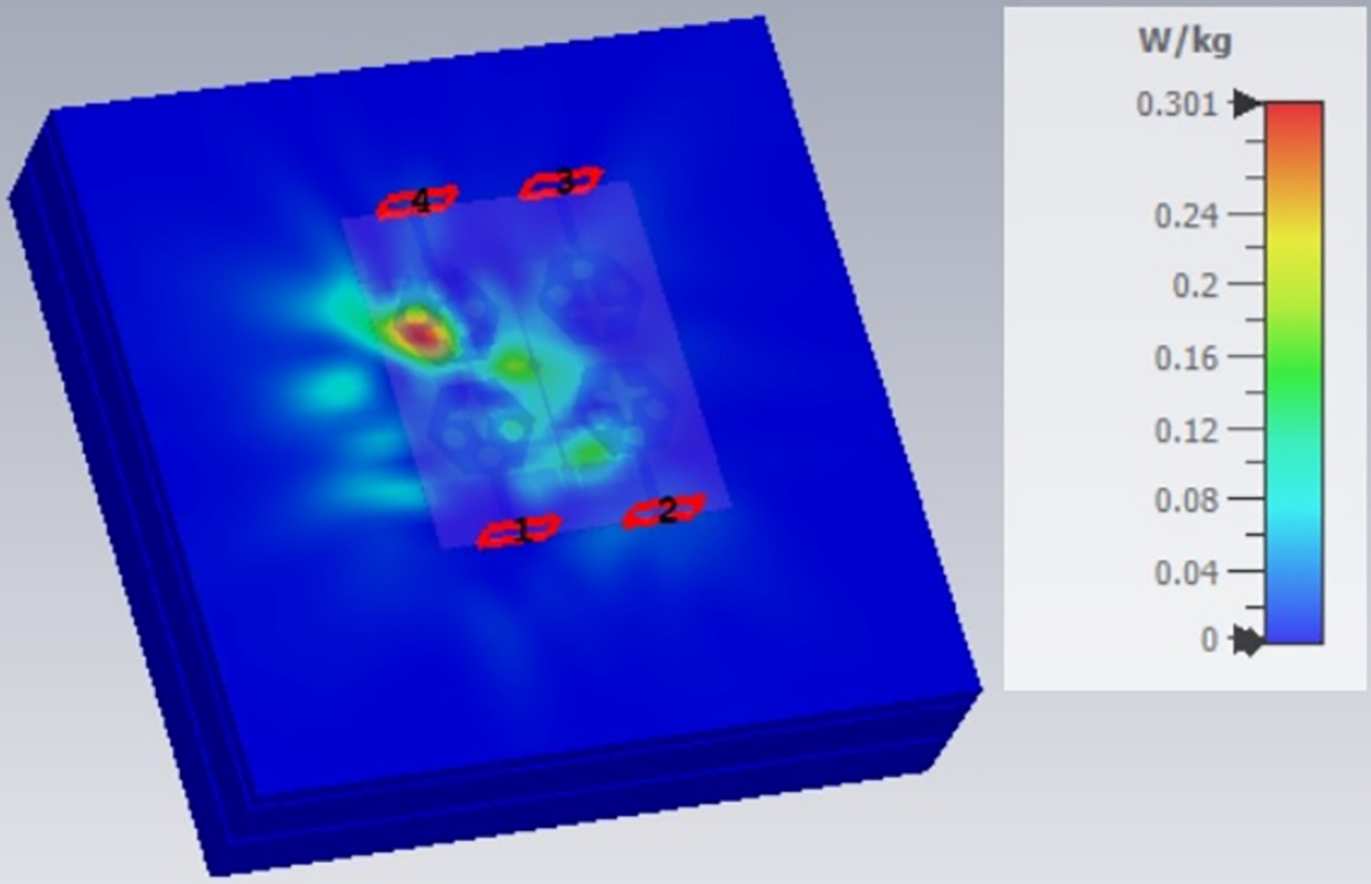
SAR analysis at 60.0GHz.

**Fig 74:**
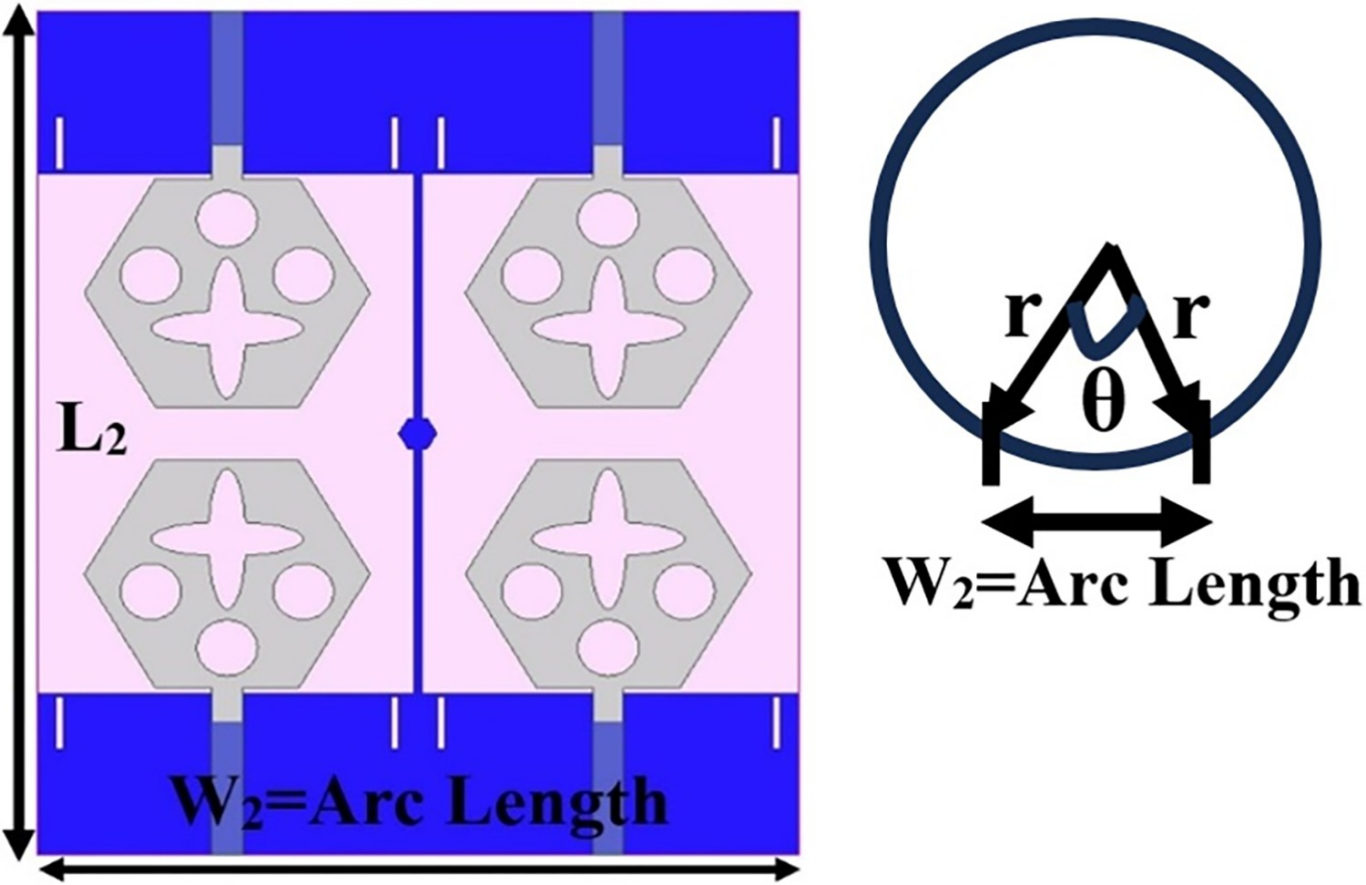
Radius Calculation.

E-plane and H-plane patterns do almost trace the omnidirectional and di-pole-like patterns with lower values of cross-polarizations. However, at the n263-60.0GHz band, these patterns do deteriorate but are useful for applications in both the principal planes. In Band 1, the applications include X-band, Ku-band, and FR2 bands. The four-port MIMO antenna is placed above the tissue model with three layers corresponding to skin, fat, and muscle. The SAR values from Figs 68–73 corresponds to 1.01W/Kg at 10.0GHz in X-band, 0.28W/Kg at 15.0GHz in Ku-band, 0.475W/Kg at 26.0GHz in FR-2, 0.588W/Kg at 28.0GHz in FR-2 and 0.301W/Kg at 60.0GHz in FR-2 band. The SAR values obtained after simulation at all the above frequency points are below 1.60W/Kg and ensure safer application for On-body application for higher-data rate transmission in future hand-held devices such as smartphones on 5G/6G platforms, tablets, laptops, smart watches, Satellite and RADAR applications. Fig 74 shows the calculation of the radius of the bending cylinder for angles of θ = 30° and 45°. Eq (28) shows the calculation of the radius which is given below:

$$W_{2}=r\times \theta$$ (28)

Where W_2_ is the width of the proposed MIMO_DWMB_ antenna which is also the arc length of the bending cylinder. The radius at θ = 30° is 38.20mm and for θ = 45°, the value of the radius is 25.50mm.

**Fig 75:**
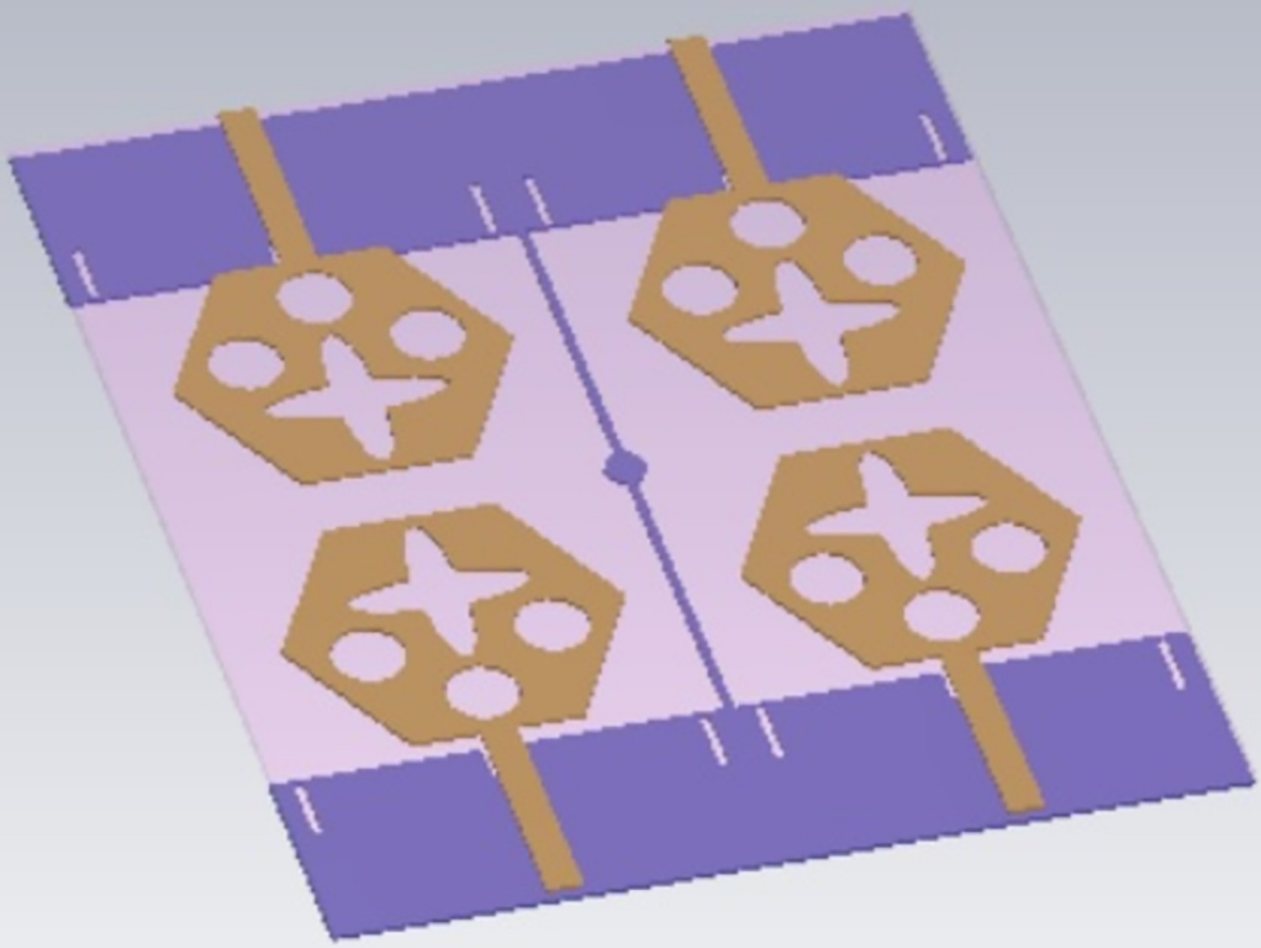
Conformal analysis at SAR analysis at 0°.

**Fig 76:**
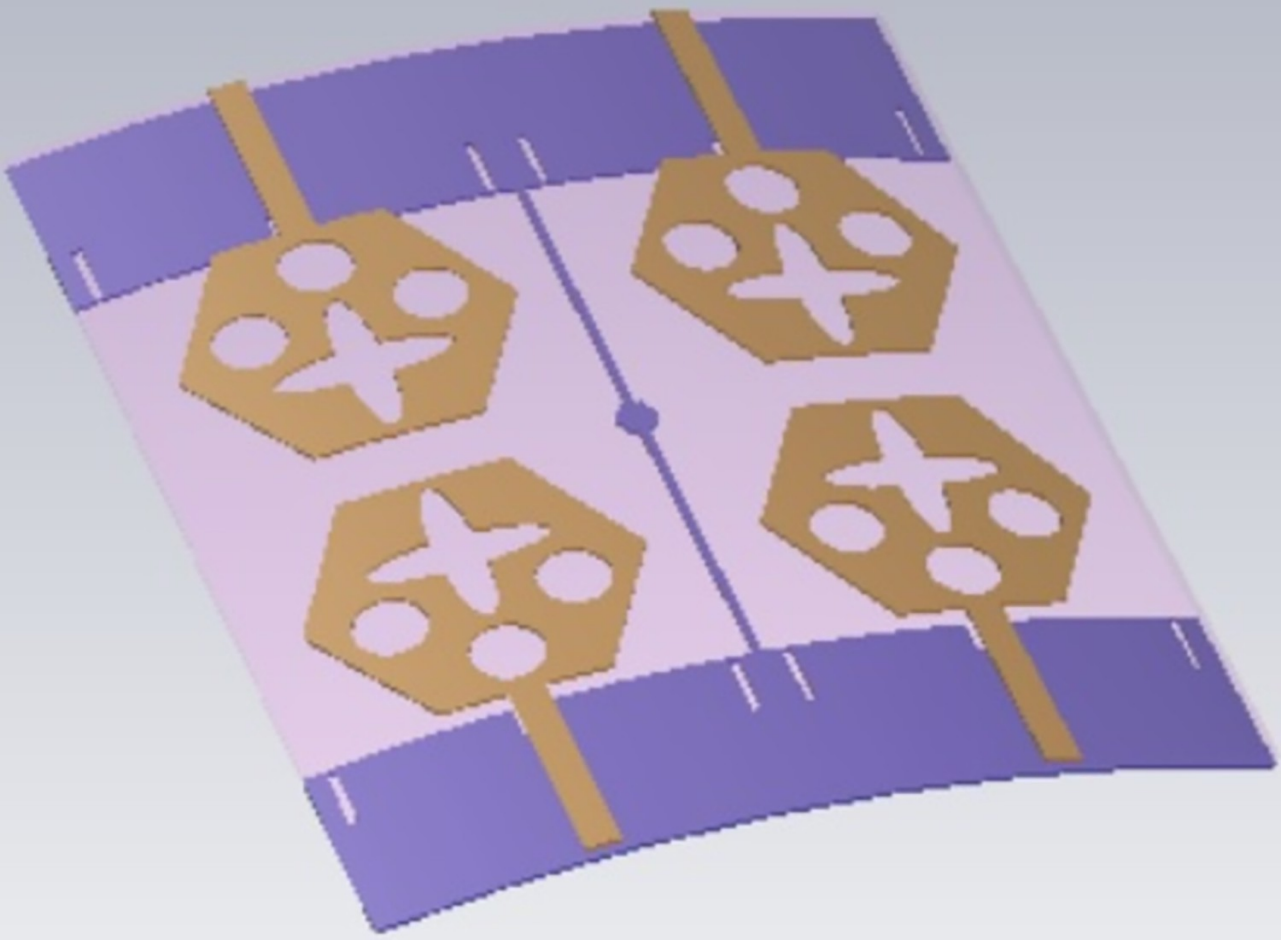
Conformal analysis at SAR analysis at 30°.

**Fig 77:**
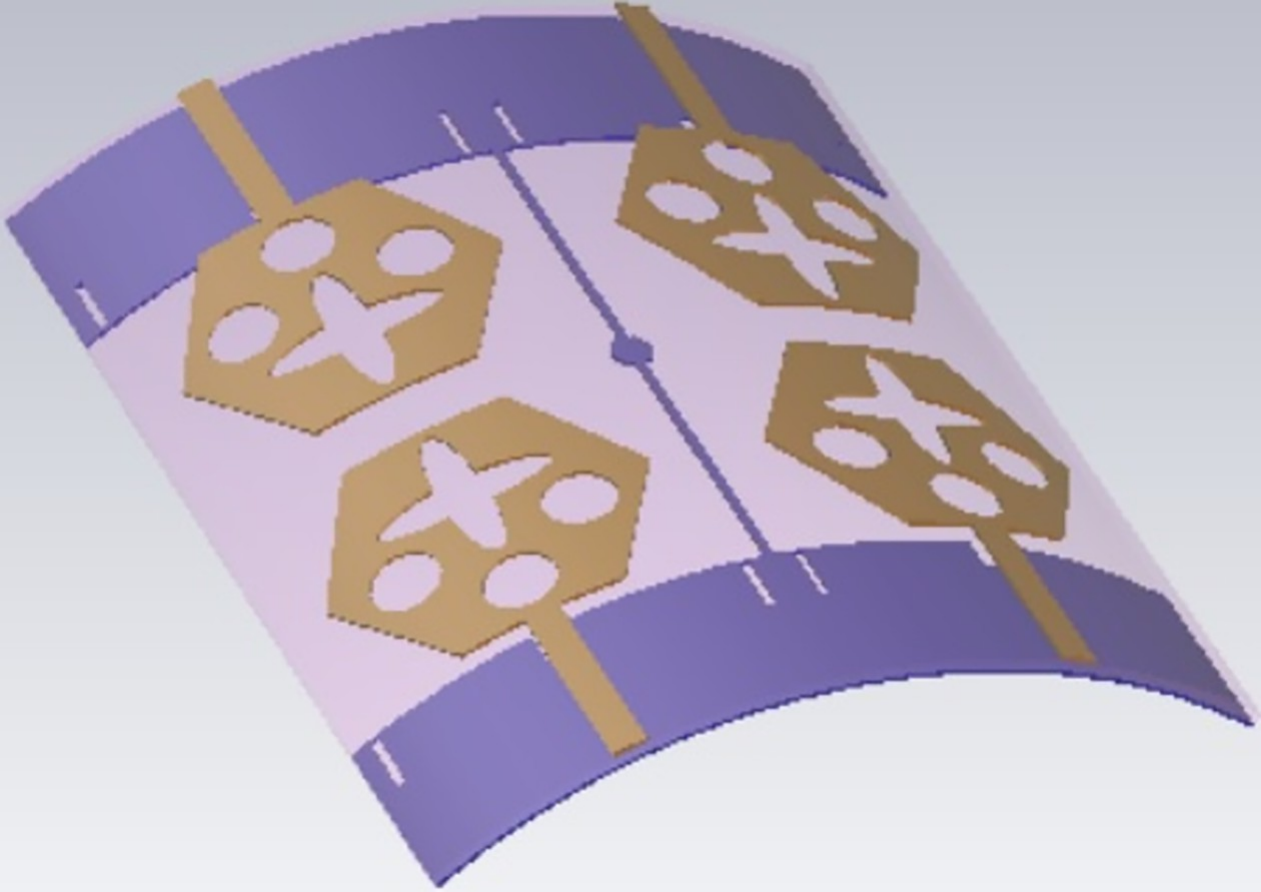
Conformal analysis at SAR analysis at 45°.

Figs 74–77 also shows the flexible capability of the proposed four-port MIMO antenna. The bending analysis is subjected at 0° (no-bending), 30°, and 45° shown in Figs 75–77.

**Fig 78:**
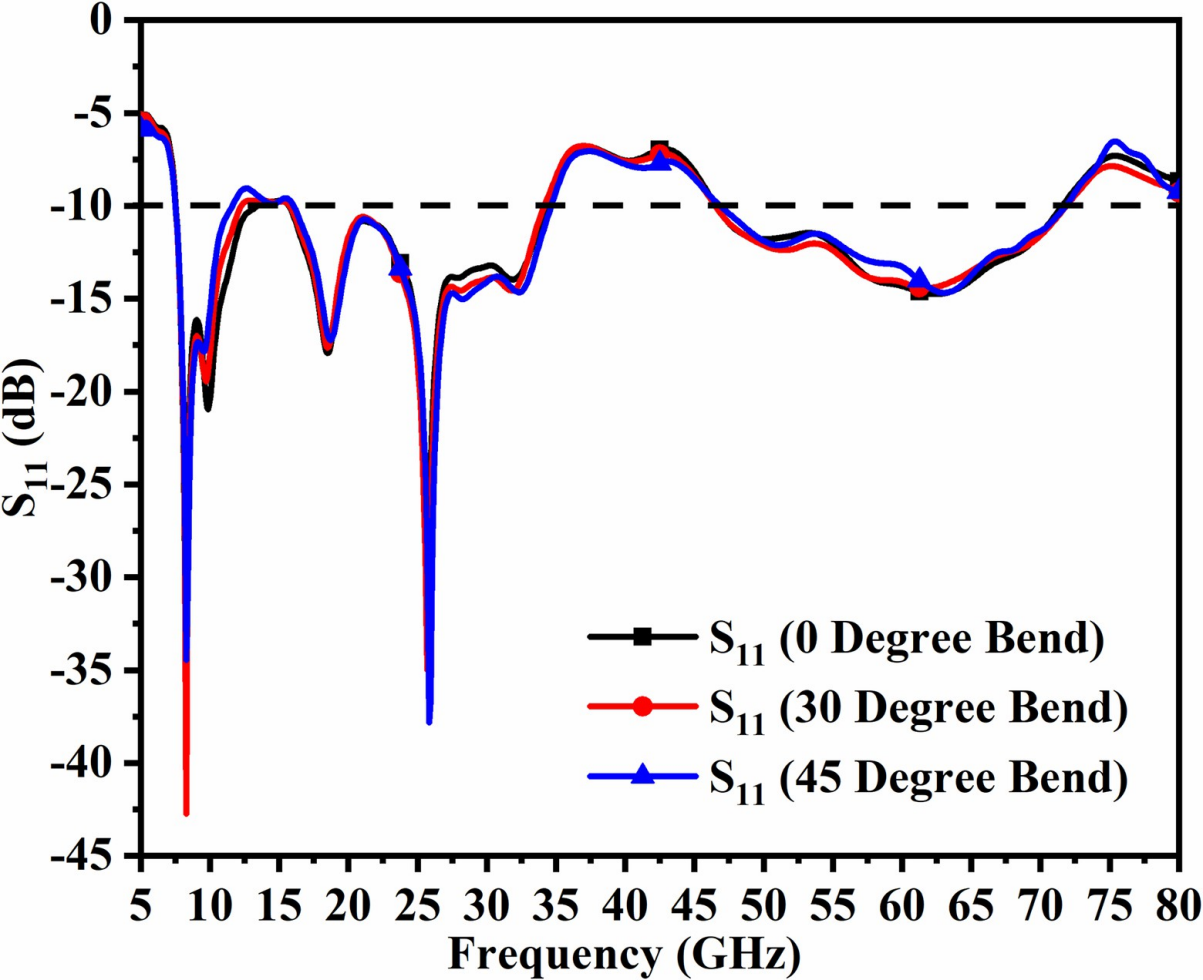
S_11_-parameter (conformal).

**Fig 79:**
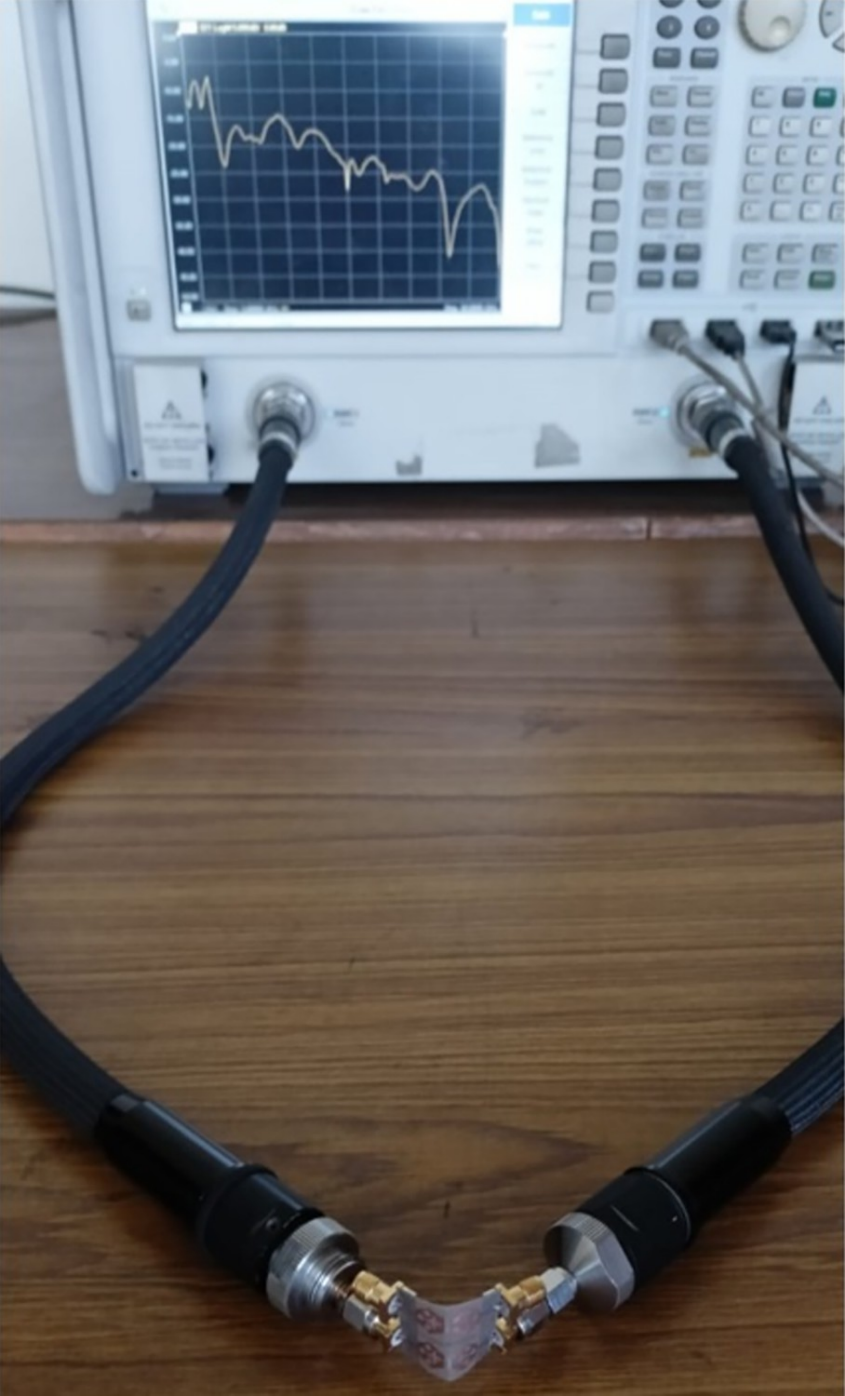
VNA screenshot with bending of the MIMO antenna.

The bending of the antenna has not much effect on the S_11_ parameter extracted in each case and the plot shown in Fig 78 overlaps with one another in Band 1 and Band 2. However, there is deviation observed in Band 2 but within the desired bandwidth. The corresponding bandwidths with different angles are tabulated in Table 5 given above. Fig 79 shows the Four-Port MIMO_DWMB_ antenna attached with VNA and shows the bending capability of the designed antenna with high values of transmission coefficient. Table 6 shows the comparison of the proposed four-port MIMO antenna with present state-of-the-art published research papers. The proposed antenna out-classes the other reported work in many ways such as the capability of the antenna generating two wider-impedance bandwidths with higher bandwidth exclusively covering wide 60.0GHz bandwidth. The proposed antenna not only offers desirable diversity performance but also finds applications in nine-wireless bands. The proposed antenna is tested for conformal characteristics which can be easily integrated with applications in flexible-electronics and wireless body-area-networks. The proposed work including multi-band characteristics can be deployed for X-Band satellite communication, VSAT applications in Ku-band, License free ISM band at 24.0GHz (24.15GHz-24.25GHz), Vehicle-to-Infrastructure UWB band application in 24.0GHz, uplink/downlink 5G-NR bands in millimeter-FR2 bands and n263.

**Table 5:** Bandwidth for bending angles of the antenna.

Bending in degrees	Band 1 (GHz)	Band 2 (GHz)
0° (no-bending)	7.475GHz-34.325GHz	46.625GHz-71.525GHz
30° (r = 38.20mm)	7.483GHz-34.025GHz	46.550GHz-71.90GHz
45° (r = 25.50mm)	7.482GHz-34.625GHz	46.925GHz-71.825GHz

**Table 6:** Comparison of the proposed work with previously published work.

Ref. No.	Size (mm^2^)	Ports/Bandwidth	Maximum Efficiency (%) at Frequency (GHz)	Isolation (dB)	ECC	DG (dB)	TARC (dB)	CCL b/s/Hz	Gain	Conformal Capability	SAR Analysis/Values (W/Kg) w.r.t. Frequency (GHz)	Potential Applications
[29]	65.0×65.0	4	97% at 10.0GHz	>22.0	<0.01	>9.999	<-10.0	NA	4.00	YES	1.04 at 4.0GHz	UWB
	(0.88λ_0_×0.88λ_0_)	2.90–10.86									0.59 at 6.0GHz	
	
											0.89 at 8.0GHz	
[37]	20.0×20.0	4	94.3% at 27.10GHz	>10.0	<0.01	>9.996	<-4.0	<0.38	7.17	YES	0.366 at 11.0GHz	N257, n258, n261, K-Band, Ku-Band
											0.313 at 15.0GHz	
	(0.70λ_0_×0.70λ_0_)	8.31–36.14										
											0.424 at 20.0GHz	
											0.418 at 24.0GHz	
											0.377 at 28.0GHz	
											0.309 at 30.0GHz	
[45]	55×55	4	NC	>25.0	<0.17	NC	NC	NC	4.00	YES	NO	Sub-6 GHz 5G
	(0.612λ_0_×0.612λ_0_)	3.34–5.01										X-Band
		8.90–9.20										
[49]	65×65	4		>15.0	<0.05	>9.99	<-10.0	<0.40	5.08	YES	1.29 at 3.39GHz	ISM
											1.47 at 4.75GHz	
												WiFi
	(1.10λ_0_×1.10λ_0_)	3.10–9.60	81% at 7.10GHz									
												Sub-6 GHz 5G
												WLAN
		
											1.71 at 8.10GHz	
*P	20.0×24.0 (0.61λ_0_×0.74λ_0_)		98.55% at 10.90GHz	>15.0	<0.01	≈10.0	<-4.0	<0.12	5.12	YES	1.01 at 10.0GHz	Uplink/Downlink (7.25GHz-8.320GHz) X-Band, Ku-Band, ISM Band (24.0GHz), 24.0GHz UWB Band, n258, n257/n261, n262, n263
											0.280 at 15.0GHz	
											0.475 at 24.0GHz	
		4										
		7.27–34.32										
		46.54–71.52									0.680 at 26.0GHz	

											0.588 at 28.0GHz	
											0.301 at 60.0GHz	

## 5. Conclusions and future scope

In this work, a four-port_DWMB_ MIMO-antenna was investigated for diversity performance, conformal capabilities, and on-body applications. The hexagonal-geometrical patch with four-etched slots and ground with one-rectangular slot with pair of etched slits resulted in two bands of operations (band 1: 7.27GHz-34.32GHz & Band 2: 46.54GHz-71.52GHz). The orientations of the radiating patch and connected partial-ground offered high diversity performance with ECC_DWMB_<0.02, DGDWMB>9.998dB, TARCDWMB<-4.0dB, and CCLDWMB<0.40 b/s/Hz. The time-domain analysis for a two-port MIMODWMB antenna with a transmitted short-sinusoidal pulse was received without distortion when a pair of identical MIMODWMB-systems were separated by distance satisfying far-field condition and variation of group delay within ±0.30ns. The average peak gain of 4.52dBi and stable-radiation patterns with acceptable SAR within 1.60W/Kg at different resonance frequencies within the two bands suggest the proposed work is capable of deployment in the wireless environment or for on-body applications. The proposed MIMO work can be further extended to array-configuration which can be executed by designing unique corporate feeding network to achieve high gain. This will also provide narrow beam which can be highly directive ensuring more power received by the receiver. Additionally, FSS (frequency-selective-surface) can be designed for more absorption of the minor lobe which can further reduce the Sar values. The unnecessary transmission of undesired bands can be stopped by reconfiguring the antenna using active switching devices like diodes.

## Supporting information

S1 Data (XLSX)
